# Genomic and palaeoclimatic data reveal Pleistocene adaptation and diversification of non-model bluegrasses (*Poa* sect. *Stenopoa*) in cold, arid environments

**DOI:** 10.1038/s42003-026-10395-6

**Published:** 2026-06-05

**Authors:** Evgenii Baiakhmetov, Sirilak Radbouchoom, Ekaterina Noskova, Kyle W. Tomlinson, Roman S. Romanets, Marina V. Olonova

**Affiliations:** 1 Center for Integrative Conservation & Yunnan Key Laboratory for Conservation of Tropical Rainforests and Asian Elephants, Xishuangbanna Tropical Botanical Garden, Menglun, China; 2 Project of Institute Establishment for Sireeruckhachati Nature Learning Park, Mahidol University, Nakhon Pathom, Thailand; 3 ETH Zurich, Institute of Integrative Biology, Zurich, Switzerland; 4 University of Edinburgh, Institute of Ecology and Evolution, Edinburgh, UK; 5 Tomsk State University, Biological Institute, Tomsk, Russia

**Keywords:** Speciation, Biogeography, Evolutionary ecology, Plant evolution, Population dynamics

## Abstract

Rapid plant radiations under climatic oscillations are widely recognised, yet our understanding of how taxonomically complex, polyploid-rich lineages diversify in extreme environments remains limited. Here, we investigated non-model Eurasian bluegrasses (*Poa* sect. *Stenopoa*), a challenging group inhabiting cold deserts, alpine grasslands, and semi-arid steppes. We utilised DArTseq genotyping of 373 individuals to infer phylogenetic relationships, identify key adaptive traits, test evolutionary scenarios, estimate demographic histories, and integrate palaeoclimatic niche reconstructions to trace the role of glacial–interglacial cycles in shaping diversification. Our findings reveal three major genetic groups that diverged during the Middle Pleistocene, partly uncoupled from morphological differentiation. Demographic analyses indicate divergence from a small ancestral population, followed by niche partitioning, secondary contact, and episodic gene flow promoted by glacial oscillations. Morphological traits associated with cold and arid tolerance further demonstrate ecological adaptation beyond genetic divergence. This research highlights how climate fluctuations and episodic hybridisation structure the diversification of this complex polyploid lineage. Moreover, our results illustrate a general framework for resolving evolutionary histories of challenging taxonomic groups, underscoring that integrative genomic and palaeoclimatic approaches can reveal mechanisms of rapid speciation in non-model taxa under changing environments.

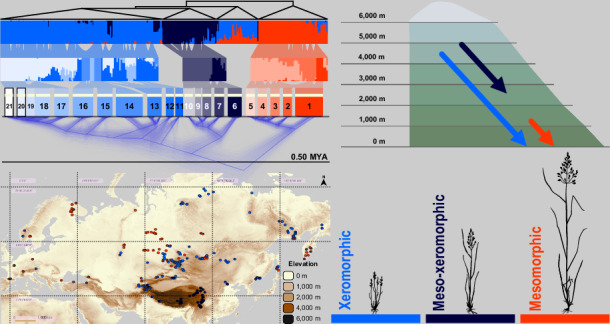

## Abstract (web summary)

Genomic and palaeoclimatic data reveal how climate fluctuations drove the rapid Pleistocene diversification and ecological adaptation of taxonomically complex Eurasian bluegrasses (*Poa* sect. *Stenopoa*) in cold and arid environments.

## Introduction

Mechanisms of adaptation to environmental changes are central to speciation in living organisms. In plants, climatic perturbations play a crucial role, often triggering episodes of whole-genome duplications (WGDs; i.e., polyploidy or genome doubling). Many WGDs have been associated with major climatic and ecological transitions, including the late Miocene–Pliocene climate shifts^1^ and the more recent Pleistocene–Holocene transition, when many domesticated grasses underwent genome doubling events^2,3^. Among the mechanisms of polyploid formation, WGD may follow after hybridisation (allopolyploidy), where genome doubling may restore chromosome pairing and fertility in otherwise sterile hybrids^4^. This process triggers widespread structural rearrangements and enhances genetic redundancy and novelty, providing allelic resources for adaptation and speciation^5,6^. These genomic rearrangements are especially prevalent in grasses (Poaceae), a family known for ecological dominance, rapid radiation, and widespread polyploidisation^7^. However, while the macroevolutionary impact of WGD has been widely studied, the microevolutionary processes that shape diversification in polyploid-rich grasses under episodic climatic stress remain insufficiently understood.

The Poaceae family has colonised a wide spectrum of environments, with C3 species thriving predominantly in cool, temperate regions, such as northern latitudes and higher altitudes, where moisture is more consistent and temperatures are moderate^8,9^. In contrast, C4 grasses excel in warm, dry tropics and subtropics, including savannas and arid plains, where their specialised photosynthetic pathways enable them to cope with high heat and water scarcity^8,9^. This adaptability has allowed grasses to flourish from arid steppes and alpine meadows to tropical regions, highlighting their evolutionary plasticity. However, the evolutionary mechanisms often underlying this success, such as frequent hybridisation and polyploidy, present a major scientific challenge. These processes generate reticulate histories and blur species boundaries, creating profound taxonomic complexity and leading to exceptional difficulty in resolving evolutionary relationships or uncovering the precise drivers of speciation using traditional methods. Untangling the history of such groups therefore requires an integrative approach that can simultaneously assess genomic divergence, phenotypic adaptation, and the environmental context in which they evolved.

The genus *Poa* L., one of the most widespread and taxonomically complex C3 grass groups, exemplifies these dynamics. Distributed globally^10–12^, the genus plays critical roles in ecosystem functioning, from stabilising soils in alpine regions to supporting herbivore communities in steppes. Its species exhibit substantial morphological plasticity attributed to pervasive polyploidy (occurring in ca. 91% of *Poa* species^13^), hybridisation^14^, and rapid radiation^15–18^. Molecular dating analyses suggest these grasses began diversifying around 11–15 million years ago (Mya)^18–20^, aligning with the global transition from forests to grasslands in the Miocene^21^. Despite advances in molecular phylogenetics^22–26^, the genus remains taxonomically challenging due to reticulate evolution and limited diagnostic morphological traits^23,27,28^. This complexity positions *Poa* as an ideal system for studying microevolutionary processes in grasses.

**Fig. 1:**
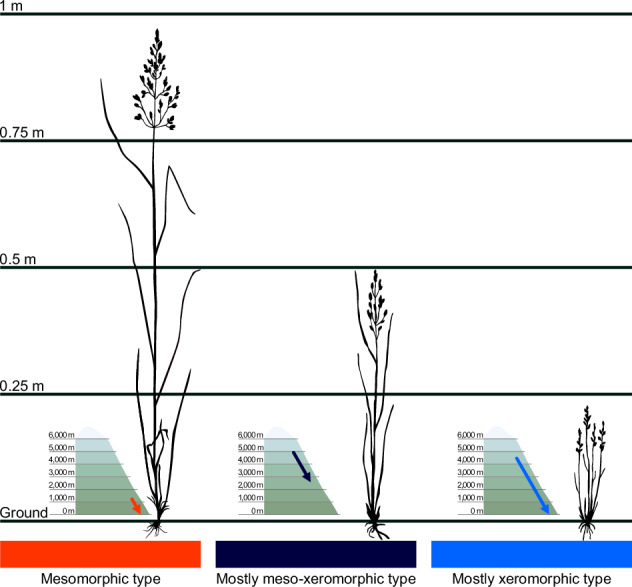
Visual overview of key morphotypes in the section *Stenopoa.* The figure provides a visual reference for the ecologically distinct morphotypes central to this study. While the taxa exhibit a general trend in height reduction (see Morphometric analysis; Fig. 4), significant size overlap exists in nature due to morphological variation. The red mesomorphic group (moderate moisture-adapted) comprises relatively tall plants (up to 150 cm) found in comparatively humid habitats such as meadows and forest glades and is common in the lower and middle mountain belts. The dark blue group (intermediate dry-adapted) includes mesomorphic and xeromorphic species adapted to various temperature and moisture conditions, ranging from tough dwarf plants of 5–10 cm with narrow and compressed panicles in the cold deserts of Tibet and Qinghai to soft mesomorphic specimens of 50–60 cm found in the humid coniferous forests of the Himalayas and the Hengduan mountains. The blue group (extreme dry-adapted) represents plants of semi-arid regions, exhibiting a moderately xeromorphic phenotype in steppes (reaching 50–60 cm in height) and an extremely xeromorphic phenotype in drier areas and high-mountain cold grasslands (dwarfs of 5–25 cm). The coloured single-sided arrows indicate the altitude range of the “pure” taxa (i.e., non-hybridised forms) identified in the study (Supplementary Data 1).

Within *Poa*, section *Stenopoa* Dumort. offers a compelling model for investigating adaptation to extreme environments with many species thriving in Asian cold deserts, high-altitude grasslands, and semi-arid steppes (Fig. 1). The section is primarily distributed across extratropical regions of Eurasia, reaching its highest morphological diversity in the mountains of Central and Middle Asia, where a distinct morphological trajectory of adaptation to cold and arid habitats is evident. This specialisation makes *Stenopoa* an ideal system for studying evolutionary mechanisms under climatic stress. Its remarkable cytotype diversity and phenotypic plasticity further underscore its utility as a model to dissect how polyploid lineages navigate climatic upheaval^14,29–31^.

Taxonomically, section *Stenopoa* represents one of the most challenging groups within the genus, characterised by profound disagreements regarding species delimitation. Estimates of diversity vary drastically depending on the taxonomic authority and the species concept applied, specifically the conflict between narrow and broad morphological definitions (i.e., *sensu stricto* and *sensu lato*, respectively). For instance, a critical revision of Eastern European taxa alone recognised 30 species and 8 varieties^32^, recent treatments of the broader Russian flora recognise 51 species^33^, while the Flora of China lists approximately 120 species attributable to this section^11^. By contrast, revisions favouring a broader morphological species concept accept only about 15 species for similar geographic ranges, treating many local variants as infraspecific taxa^10,34^. This taxonomic instability arises from the same complex evolutionary history characteristic of the genus, where shifts in breeding systems, including the transition to facultative apomixis^35^ and the evolution of dicliny^26,36^, interact with polyploidy^17^ and hybridisation^14^. Such processes, compounded by significant morphological variation and a lack of diagnostic characters, generate a continuum of “microspecies” defined by minor morphological traits where taxonomic boundaries remain poorly understood.

Consequently, resolving the evolutionary history of *Stenopoa* requires moving beyond traditional alpha-taxonomy to investigate the underlying genomic structure and phylogeographic history of these complex lineages. Divergence estimates for the section remain contentious, ranging from 0.61 Mya^18^ to 2.89 Mya^19^, reflecting unresolved evolutionary timelines. While recent genomic resources for *Poa*^37–39^ have clarified the role of divergent diploid ancestors in polyploid plasticity (e.g., *P. annua* L.^40^), integrative analyses linking genomic, morphological, and environmental data are lacking. This gap limits our understanding of how climatic fluctuations shaped polyploid diversification and niche expansion in grasses, offering insights into their resilience and conservation in the context of ongoing climate change.

Here, we reconstruct the evolutionary history of *Stenopoa* by integrating genomic, morphological data, and climatic niche modelling. We sought to understand the ecological circumstances under which Eurasian bluegrasses began to rise, how population dynamics were affected by environmental perturbations, and when they began diversifying and occupying new habitats. Specifically, we employed DArTseq genotyping and climatic niche modelling to (1) delineate evolutionary units supported by morphology and molecular evidence; (2) estimate divergence times between detected genetic clusters; (3) uncover past demographic history; (4) test evolutionary scenarios shaping adaptation and speciation in the section; (5) explain how climatic fluctuations could have facilitated the spread of the section across Eurasia; and (6) synthesise available cytological data to contextualise these genomic findings.

## Results

### Genetic structure and molecular dating

The final dataset comprised 6958 single nucleotide polymorphisms (SNPs) genotyped from 373 individuals representing 37 morphologically defined species distributed across Eurasia (Supplementary Data 1–3; External Supplementary Dataset 1^41^).

According to the clustering methods (unweighted paired group method with arithmetic mean, UPGMA; and Bayesian clustering method, STRUCTURE), the dataset can be optimally divided into two clusters (*K* = 2; delta *K* > 6000; Supplementary Fig. 1; External Supplementary Dataset 1^41^). However, we proceeded with *K* = 3 (delta *K* ≈ 2500), because it revealed an additional distinct genetic group consisting of pure specimens (proportions of membership, Q ≥ 0.99). Thus, hereafter we refer to these three main clusters as “red” (Cluster I), “dark blue” (Cluster II), and “blue” (Cluster III).

In terms of distribution, Cluster I largely inhabits lowlands and uplands in the Far East and Siberia (5–752 m), extending to the Urals (259–965 m), while single pure individuals were also detected in the Republic of Karelia (11 m) and Kurchumskiy Khrebet in Kazakhstan (1331 m). Cluster II represents a well-defined geographical area in the central and eastern part of the Tibetan Plateau, collected at altitudes ranging from 2612 to 5375 metres above sea level. Cluster III has a broad geographic range, ranging from mountainous regions in the northwestern Tibetan Plateau / Eastern Tajikistan at altitudes from 3027 to 4231 m through the Eastern Tian Shan mountains (northwestern China, 1938 m), western Mongolia (1568–3031 m) and reaching Russian territories in the Altai Mountains (1382–4495 m), the Sayan Mountains (932–2228 m), the Kolyma Mountains (1484 m) and the Kular Range (1183 m), as well as occupying adjoined uplands and lowlands, including those from nearby Lake Baikal (462–496 m) and the Urals (526 m).

The Neighbour-net phylogenetic network essentially supports the grouping into three pure clusters connected by reticulations (admixed groups), reflecting the presence of gene flow between Clusters I and III, and Clusters II and III, but not between Clusters I and II. Principal Component Analysis (PCA) combined with the STRUCTURE result confirmed a similar pattern, separating Cluster I from Clusters II and III by the first principal component (24.0% of inertia), while the second principal axis (6.6%) further differentiates Clusters II and III. In addition, PCA and STRUCTURE clearly demonstrate the extent of hybridisation between Clusters I and III, as well as between Clusters II and III, while only one admixed individual was detected between Clusters I and II (ID14801, Supplementary Data 1). However, samples incorporating all three genetic groups were also identified, indicating the presence of common contact zones or gene flow between Clusters II and III and subsequent hybridisation with Cluster I. The divergence between the three clusters representing only pure individuals was also supported by significant genetic distances (*F*_*ST*_) between Clusters I and II (*F*_*ST*_ = 0.655), Clusters I and III (*F*_*ST*_ = 0.642), as well as between Clusters II and III (*F*_*ST*_ = 0.436). An alternative metric, the Absolute allele Frequency Difference (*AFD*), exhibited weaker differentiation: Cluster I vs. Cluster II (*AFD* = 0.181), Cluster I vs. Cluster III (*AFD* = 0.137), and Cluster II vs. Cluster III (*AFD* = 0.082), consistent with observed hybridisation. The extent of shared ancestry between Clusters II and III is also supported by a lower number of fixed alleles (*n* = 24) in comparison with pairs Clusters I and II (*n* = 172), as well as Clusters I and III (*n* = 94). Furthermore, the presence of private alleles (alleles that are only found in one cluster) demonstrates that all three detected clusters keep unique genetic traits despite gene flow between them (Supplementary Data 4).

Since the initial analyses of the 373 genotyped individuals resolved only three main clusters supported by geographical distribution, we employed an additional hierarchical clustering approach (see Methods) using STRUCTURE, Neighbour-net network, and PCA to perform a targeted investigation at a finer scale within each of the three main clusters separately (Fig. 3 and Supplementary Fig. 1). From the initial set of 37 species, we identified 21 genetically cohesive groups represented by 52 pure individuals. Importantly, this division was largely supported by the multispecies coalescent model (MSC) implemented in SNAPP, which allowed us to estimate divergence times among and within Clusters I–III. The initial split in the section occurred around 0.59 million years ago (Mya; 95% Highest Posterior Density, HPD: 0.82–0.37 Mya) when Cluster I (*n* = 13) began to separate from Clusters II (*n* = 10) and III (*n *= 29). The subsequent segregation between the latter pair dated around 0.41 Mya (95% HPD: 0.58–0.24 Mya). Diversification in Cluster I started around 0.13 Mya (95% HPD: 0.20–0.08 Mya), in Cluster II ca. 0.22 Mya (95% HPD: 0.33–0.13 Mya), and in Cluster III about 0.30 Mya (95% HPD: 0.46–0.17 Mya).

**Fig. 2:**
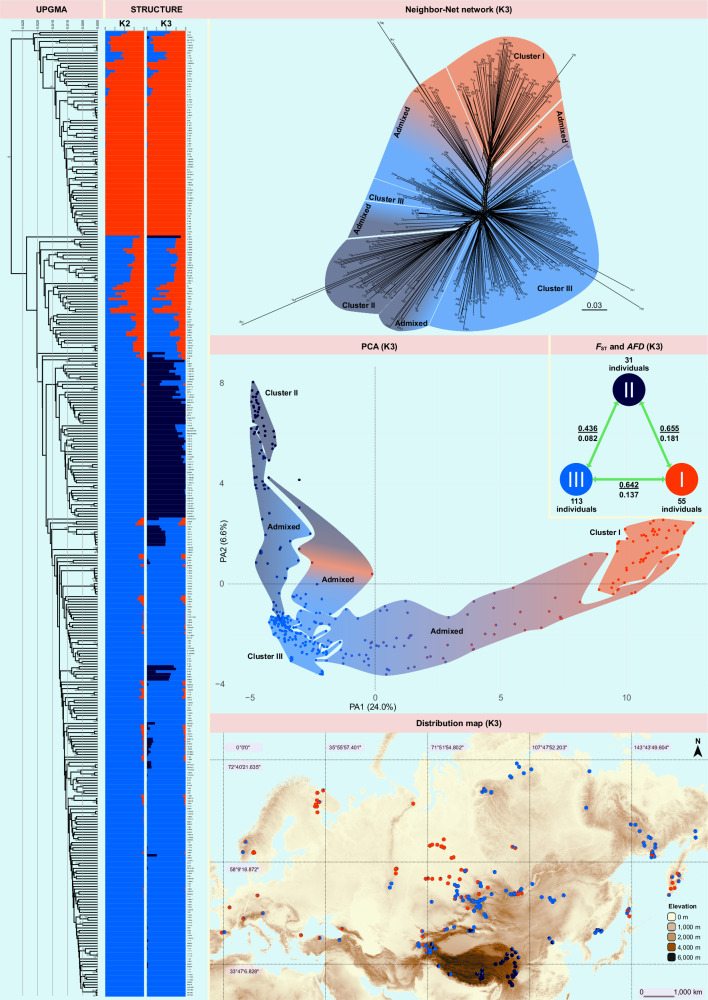
Geographic distribution and results of clustering analyses. The UPGMA illustrates the relationships among 373 bluegrass individuals; only bootstrap values  ≥  60% are shown; the x-axis represents the Hamming Distance. The STRUCTURE ancestry plot shows individual assignments at *K* = 2 and *K* = 3; the x-axis indicates the proportions of membership (Q) of each individual. The Neighbour-net network and PCA display relationships among the three genetic clusters defined based on STRUCTURE at *K* = 3: Cluster I (red), Cluster II (dark blue) and Cluster III (blue). In the PCA, the shaded areas specify pure clusters (Q ≥ 0.99), while individuals of admixed nature are shown by their position within gradients between the corresponding ancestral clusters (e.g., admixed individuals between Clusters I and III fall in the transition zone shaded from blue on the left to red on the right). The numbers on the tips in the Neighbour-net network plot represent the individual IDs (Supplementary Data 5); the scale bar represents nucleotide substitutions per position; the pie charts in the PCA plot represent genetic membership (*K* = 3). The fixation index (*F*_ST_, numbers on top) and absolute allele frequency difference (*AFD*, numbers on bottom) were calculated between the pure genetic clusters. The distribution map of the analysed specimens illustrates the geographic distribution of each cluster, with exact coordinates provided in Supplementary Data 1. The pie charts on the map represent genetic membership (*K* = 3).

**Fig. 3:**
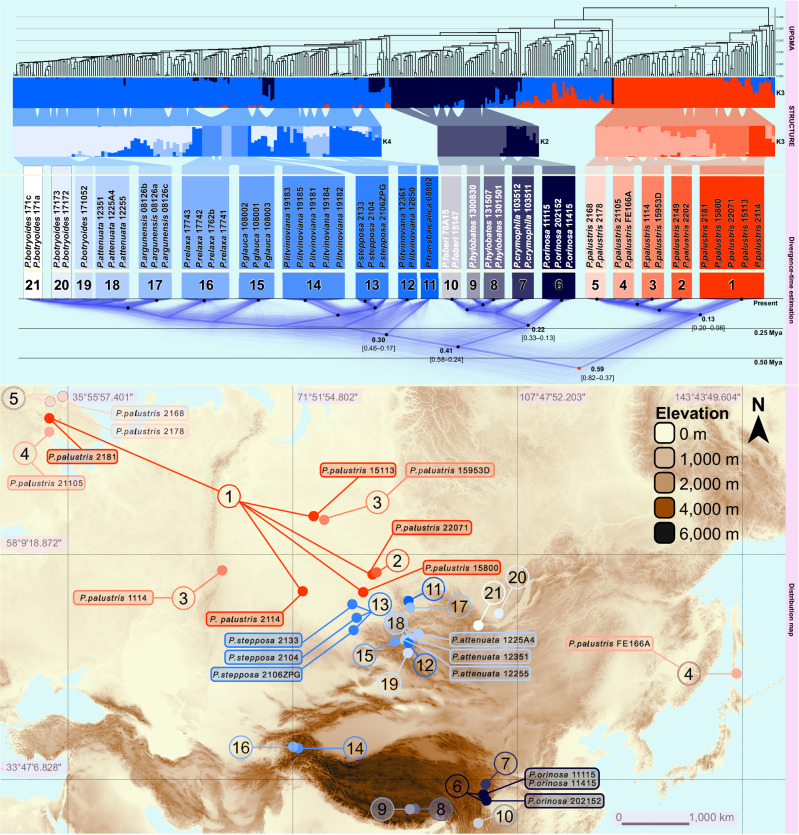
Geographic distribution and schematic division of pure genetic clusters into subgroups. The UPGMA and STRUCTURE results (*K* = 3) are from Fig. 2. The subsequent division into 21 groups is shown in full in Supplementary Fig. 1. Divergence date estimates inferred by SNAPP: blue coloured trees represent the most probable topology; the black circles denote nodes with Bayesian Posterior Probabilities (BPPs) ≥ 0.9; the red circle indicates the presumed divergence time split set as a reference (BPP = 1); numbers at the selected nodes represent mean divergence time estimates with 95% HPD intervals in the brackets; the scale shows divergence time in million years ago (Mya). The distribution map shows the locations of analysed specimens (the exact coordinates are provided in Supplementary Data 1); the coloured circles depict genetic groups defined based on STRUCTURE and validated with SNAPP.

The fine-scale distribution of the 21 genetically pure sub-clusters largely mirrors and refines the broader patterns described above (Figs. 2, 3). Groups 1–5, representing Cluster I, occupy the broadest geographic range, extending across Russian lowlands (7–228 m) from Sakhalin to Murmansk Oblast, consistent with the general description of Cluster I as a widespread lowland lineage (Fig. 2). Groups 6–10 (Cluster II) are confined to the central and eastern parts of Tibetan Plateau at high altitudes (3085–5375 m), corroborating the restricted montane distribution inferred for this cluster. Groups 11–21 (Cluster III) occupy both mountainous regions (above 3000 m in the northwestern Tibetan Plateau and Altai Mountains) and lowland areas (e.g., 134 m in Altai Krai), reflecting the ecological breadth of this cluster noted in the general description. Thus, the sub-cluster analysis confirms the major distributional patterns while revealing additional structure within each lineage.

Further taxonomic refinement is possible but will require additional investigation to properly define the status of these 21 genetic groups. For instance, among the genetically cohesive groups identified in our phylogenetic analysis, Cluster I is represented by a clade that includes *P. palustris* L. specimens from multiple geographic locations. Within Cluster III, groups 18–21 comprise two morphologically defined species (*P. attenuata* Trin. and *P. botryoides* (Trin. ex Griseb.) Kom.) with poorly-supported nodes (Bayesian Posterior Probabilities, BPPs < 0.9), and groups 12 and 14 together constitute a polyphyletic assemblage currently assigned to a single morphologically defined species, *P. litvinoviana* Ovcz.

### Morphometric analysis

**Fig. 4:**
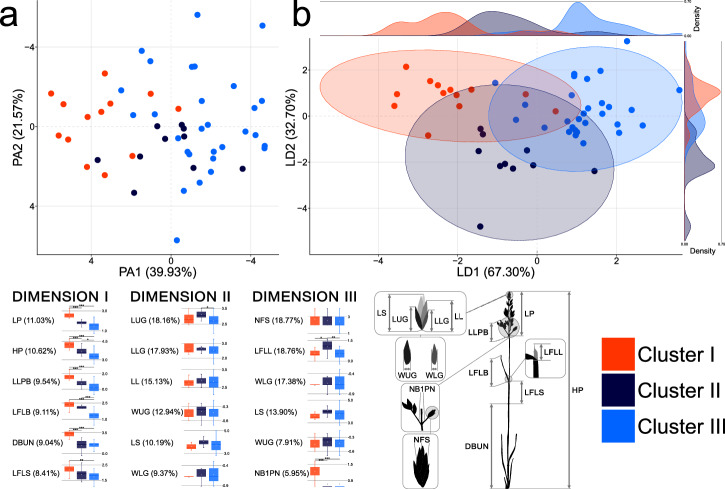
Morphological differentiation of genetically pure individuals revealed by PCA, LDA, and trait distributions. **a** PCA plot of the first two Principal Axes (PAs) and boxplots for 15 characters contributing the most to the first three PAs; length of the panicle (LP), height of the plant (HP), length of the longest panicle branch (LLPB), distance from the base to the uppermost node (DBUN), length of the flag leaf sheath (LFLS), width of the upper glume (WUG), width of the lower glume (WLG), length of the flag leaf ligule (LFLL), and number of branches at the first panicle node (NB1PN) are log-transformed; numbers in parentheses show percent contribution; asterisks denote statistically significant differences between clusters (*p < 0.05; **p < 0.01; ***p < 0.001). For the boxplots, the thick line represents the median, the white circle denotes the mean, the box limits represent the 25th and 75th percentiles (interquartile range), and the whiskers extend to the 5th and 95th percentiles; confidence intervals are provided in Supplementary Fig. 2. **b** LDA plot of the first two Linear Discriminants (LDs); shaded colour ellipses represent 95% probability contours for each cluster; marginal density plots for LD1 (top) and LD2 (right) illustrate cluster separation along each discriminant function. The illustrations depict the measured characters. Character abbreviations are defined in the main text and in Supplementary Data 6.

PCA of 21 morphological traits measured on the 52 genetically pure individuals (Supplementary Data 6; External Supplementary Dataset 2^41^) across the three genetic clusters revealed mainly two morphotypes, distinguishing Cluster I from Clusters II and III (Fig. 4a; interactive version at https://niwdoog.github.io/Fig4a). Dimension I (39.93% of the total inertia) was dominated by traits related to plant size and panicle structure (Supplementary Data 7), e.g., length of the panicle (LP), height of the plant (HP), length of the longest panicle branch (LLPB), length of the flag leaf blade (LFLB), distance from the base to the uppermost node (DBUN), and length of the flag leaf sheath (LFLS). Cluster I was characterised by positive scores along Dimension I, indicating larger values for these traits, while Cluster III scored negatively, reflecting smaller, more compact forms. These differences suggest Cluster I is more adapted to mesic, lowland habitats with its taller stature and larger panicles, while Cluster III’s compact morphology may be better suited to arid environments. Along Dimension I the clusters moderately overlap, reflecting morphological variation that may be a consequence of historical gene flow in zones where their ranges intersect, such as mid-altitude habitats.

Statistical analysis highlighted HP as the only trait significantly different across all clusters (*p* < 0.001 for Clusters I vs. II and I vs. III; *p* < 0.05 for Clusters II vs. III). Cluster I also displayed significantly greater values for LP, LLPB, and DBUN compared to Clusters II and III (*p* < 0.001 for all comparisons). LFLB differed significantly between Clusters I and III (*p* < 0.01), while LFLS showed differences between Clusters I and III (*p* < 0.001) and Clusters II and III (*p* < 0.001), but not between Clusters I and II (*p* > 0.05).

Dimension II, accounting for 21.57% of the total inertia, was influenced by floral and spikelet traits, including upper glume length (LUG), lower glume length (LLG), lemma length (LL), upper glume width (WUG), spikelet length (LS), and lower glume width (WLG). Cluster II exhibited positive scores along Dimension II, suggesting enhanced reproductive structures. However, significant differences were limited to LUG between Clusters II and III (*p* < 0.05).

Dimension III, contributing 7.55% to the total inertia, further distinguished the clusters (interactive version at https://niwdoog.github.io/Fig4a). Cluster II scored positively for traits such as the number of florets per spikelet (NFS), flag leaf ligule length (LFLL), lower glume width (WLG), spikelet length (LS), and upper glume width (WUG). Conversely, the number of branches at the first panicle node (NB1PN) separated Cluster I from Clusters II and III, with Cluster I showing higher values. Statistical analysis revealed significant differences for LFLL between Clusters I and II (*p* < 0.05) and Clusters II and III (*p* < 0.01), and for NB1PN between Clusters I and II (*p* < 0.001) and Clusters I and III (*p* < 0.001).

Further, to test whether a reduced set of morphological traits can accurately classify individuals into their pre-defined genetic Clusters I–III, we performed Linear Discriminant Analysis (LDA). The analysis used the same 52 genetically pure individuals from the PCA and employed seven key traits (DBUN, HP, LFLB, LP, NB1PN, WFLB, WP) selected by Recursive Feature Elimination (RFE) (Supplementary Data 8; External Supplementary Dataset 2^41^). The LDA model achieved an accuracy of 92.31% on the training (resubstitution) data (95% CI: 81.46–97.86%; Cohen’s Kappa = 0.87), significantly outperforming the No Information Rate (NIR) baseline (*p* < 0.001). Leave-One-Out Cross-Validation (LOOCV) yielded 84.62% accuracy (95% CI: 71.92–93.12%; Cohen’s Kappa = 0.74), also significantly exceeding the NIR (*p* < 0.001) and confirming robust generalisability. Cluster III was classified with the highest sensitivity (LOOCV: 93.10%; resubstitution: 96.60%), likely due to its higher prevalence in the dataset (*n* = 29 samples, 55.77% of the total). Cluster I was classified with moderate sensitivity (LOOCV: 76.92%; resubstitution: 84.62%; prevalence: *n *= 13, 25.00%), while Cluster II had the lowest sensitivity (LOOCV: 70.00%; resubstitution: 90.00%; prevalence: *n* = 10, 19.23%), suggesting greater morphological similarity with other clusters. To assess model stability, we conducted permutation-based cross-validation (100 iterations), applying RFE within each training fold. This yielded substantial agreement beyond chance (mean accuracy: 83.60% ± 10.00% SD; mean Cohen’s Kappa = 0.72 ± 0.17 SD), confirming robust generalisation across data partitions despite inherent variability.

The first two descriptive Linear Discriminant functions (LDs) explained 67.30% and 32.70% of the between-group variance, respectively (Fig. 4b). The LFLB trait was the primary contributor to both LDs, with negative coefficients (LD1: −1.61; LD2: −1.79), driving the separation of Clusters I and II from Cluster III along LD1 and Cluster II from Clusters I and III along LD2. The LP variable also had substantial influence, with positive coefficients (LD1: 1.24; LD2: 1.51), distinguishing Cluster III from Clusters I and II along LD1 and Clusters I and III from Cluster II along LD2. Additionally, DBUN (1.58) and HP ( − 1.27) significantly contributed to the differentiation between Clusters along LD2 (Supplementary Data 9). Thus, the LDA model highlights flag leaf blade length (LFLB) and panicle length (LP) as the most powerful discriminators between clusters. These traits are functionally linked to resource acquisition and reproductive investment. Their prominence as discriminators suggests that divergent selective pressures on these functional traits likely reflect trade-offs between competitive ability (e.g., in Cluster I) and stress tolerance (e.g., in Cluster III) and are central to the ecological differentiation of these lineages.

Integrating molecular data (Figs. 2, 3) with the morphological patterns identified in this study (Fig. 4), we updated the species determinations for 80 of 373 individuals (21.45%). Specifically, individuals whose initial morphology-based identifications conflicted with their robust genetic placement were reassigned to the species names that best corresponded to their respective genetic clusters. This was performed following an expert taxonomic re-evaluation that accounted for both the new molecular evidence and recent nomenclatural updates regarding species synonymy (Supplementary Data 3). However, as this study was not designed as a comprehensive taxonomic revision, a separate investigation is required to establish reliable identification keys (see limitations in the Discussion).

### Demographic reconstruction and evolutionary scenarios testing

To reconstruct the demographic history of the three genetic clusters, we employed two complementary approaches, GADMA2^42^ and Stairway Plot 2^43^, on two datasets with different SNP call rates (95% and 70%). Our strategy, detailed in the Methods, used the higher-quality 95% dataset to robustly select the best-fitting evolutionary model structure, and the larger 70% dataset to provide greater statistical power for parameter estimation within that chosen model.

**Fig. 5:**
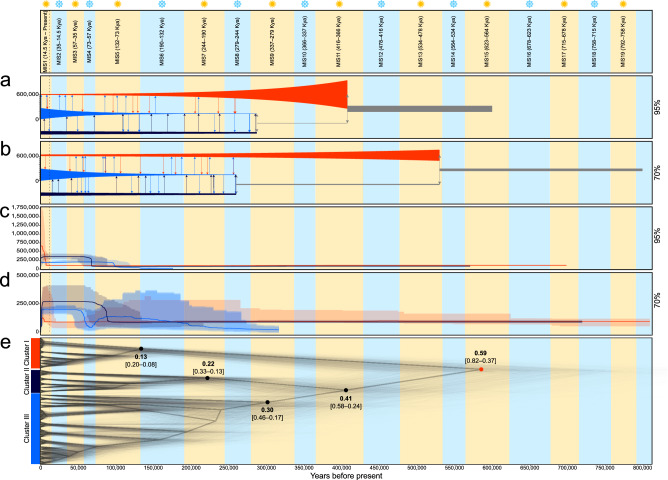
Reconstruction of population size changes from 800 Kya to the present. **a** The GADMA2 result for SNPs with a call rate of 95%. **b** The GADMA2 result for SNPs with a call rate of 70%. **c** The Stairway Plot 2 result for SNPs with a call rate of 95%. **d** The Stairway Plot 2 result for SNPs with a call rate of 70%. **e** The SNAPP result (Fig. 3) is provided for reference. At the top, sun symbols and light amber rectangles indicate warming (odd-numbered) Marine Isotope Stages (MISs), while snowflakes and light cyan rectangles depict cooling (even-numbered) stages, according to Railsback et al.^113^ (Supplementary Fig. 4). The X-axis (panel **e**) represents years; Y-axes (panels **a**–**d**) show estimates of historical effective population sizes. The red colour across all panels represents Cluster I, the dark blue – Cluster II, and the blue – Cluster III. The coloured single-sided arrows (panels **a**–**b**) indicate the direction of gene flow between the Clusters, while the grey two-sided arrows show ancestral splits. In panel **b**, the arrows were manually adjusted to indicate the exact timing of the onset of gene flow inferred by the model. The shaded areas (panels c–d) depict 95% confidence intervals. The dashed line marks the Pleistocene–Holocene transition (~11.7 Kya).

Across both datasets, the evolutionary scenarios tested with GADMA2 consistently supported a model where Cluster I represents an early-diverging lineage, while Clusters II and III share a more recent common ancestor before splitting from each other (Fig. 5a, b; Supplementary Fig. 3; External Supplementary Dataset 3^41^). This topology is fully congruent with the species tree inferred using SNAPP (Fig. 5e), confirming a robust underlying evolutionary history. All key divergence time estimates from GADMA2 fell within the 95% HPD intervals calculated with SNAPP.

The final, best-supported model, with parameters estimated from the 70% SNP dataset (Fig. 5b), indicates that the ancestral lineage of all three clusters had an effective population size (*N*e) of approximately 60,000 before the initial divergence of Cluster I from the ancestor of Clusters II and III around 530 Kya. This ancestral population of Clusters II and III (*N*e of ca. 25,000) subsequently diverged during the cooling of Marine Isotope Stage 8 (MIS8) around 260 Kya. The model revealed a complex history of bidirectional gene flow between Clusters II and III and between Clusters I and III, with no gene flow detected between Clusters I and II. Migration between the sister lineages of Cluster II and Cluster III likely began soon after their divergence (ca. 260 Kya), suggesting a history of continuous contact or sympatry. In contrast, gene flow between Clusters I and III began more recently (ca. 257 Kya), pointing to a secondary contact scenario, possibly following range expansion. The population size trajectories from this model show a long-term decline for Cluster I (from an ancestral *N*e of ca. 270,000 to 40,000), relative stability and slight growth for Cluster II (*N*e from 30,000 to 80,000), and pronounced exponential growth for Cluster III (*N*e from 8000 to 290,000).

Importantly, while the model of divergence with gene flow was robustly supported by the 95% dataset, the 70% dataset also provided credible support (lower AIC; Supplementary Data 10; Supplementary Fig. 3) for an alternative scenario where Cluster III formed via an admixture event between Clusters I and II around 260 Kya. Although this admixture model was not our final supported history due to the conflict with the higher-quality 95% dataset, it suggests that the evolutionary origin of Cluster III may be more complex than simple divergence and warrants further investigation with more extensive genomic data.

While GADMA2 identified the primary evolutionary scenario, Stairway Plot 2 analyses further provided a temporal framework for understanding the demographic history of *Stenopoa* populations, shaped by Pleistocene climatic fluctuations (Fig. 5c, d). These analyses showed that all clusters originated from relatively small ancestral populations and experienced complex demographic shifts, although the precise timing and magnitude of these events differed between the datasets. For Cluster I, the 95% dataset indicated an origin ca. 700 Kya with an effective population size (*N*e) of ca. 100,000, which remained stable until a post-glacial exponential rise to ca. 670,000 at ca. 11.7 Kya, whereas the 70% dataset suggested an earlier origin ca. 810 Kya (*N*e ca. 115,000), followed by growth between 300 and 73 Kya, a decline during MIS4 (73–57 Kya), a modest expansion to ca. 147,000 between 6 and 3 Kya, and a subsequent decline to ca. 114,000. For Cluster II, the 95% dataset showed an emergence ca. 570 Kya (*N*e ca. 80,000), a surge during MIS4 (ca. 70 Kya) to ca. 355,000, peaking at ca. 360,000, and then declining to ca. 310,000, while the 70% dataset indicated an earlier origin ca. 720 Kya (*N*e ca. 90,000), exponential growth during MIS5 (132–73 Kya) to ca. 280,000, followed by a steep reduction to ca. 50,000 over the last 3 Kya. For Cluster III, the 95% dataset suggested an origin ca. 175 Kya (*N*e ca. 13,000), with progressive expansion from ca. 132 Kya to ca. 200,000, stabilising at ca. 190,000, whereas the 70% dataset pointed to an earlier origin ca. 315 Kya (*N*e ca. 30,000), growth between 300 and 73 Kya, a decline during MIS4, a rebound to ca. 155,000 at ca. 57 Kya, and a sharp contraction to ca. 7000 in the last 300 years.

### Palaeodistribution prediction

**Fig. 6:**
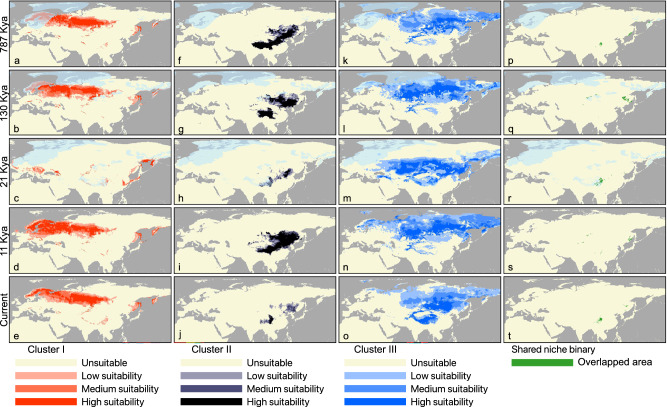
Ecological niche models built with MaxEnt based on WorldClim data. Ecological niche models show a sequence of palaeoclimatic projections for the three genetic clusters under present conditions, post-LGM warming (11 Kya; Marine Isotope Stage 1, MIS1), Last Glacial Maximum (LGM, 21 Kya; MIS2), Last Interglacial (LIG, 130 Kya; MIS5), and Chibanian age (Middle Pleistocene, 787 Kya; MIS19). Columns **a**–**e** represent Cluster I, **f**–**j** depict Cluster II, **k**–**o** show Cluster III. Panel **p** exhibits the overlapped areas between Clusters I and II, while **q**–**t** indicate the overlaps between three clusters. The light blue colour represents ice-sheet extent from Batchelor et al.^114^.

To investigate the potential environmental drivers of the detected demographic histories and to understand the geographical context of the divergence and gene flow patterns identified, we reconstructed climatic niches for the three genetic clusters over the last 787,000 years (Fig. 6; Supplementary Data 11, 12; External Supplementary Dataset 4^41^). The consensus MaxEnt modelling^44^, which integrated multiple runs for accuracy, achieved robust predictive performance with a threshold-independent metric, Area Under the Curve^45^ (AUC ≥ 0.90), and a threshold-dependent metric, True Skill Statistic^46^ (TSS ≥ 0.70; Supplementary Data 13).

The three genetic clusters exhibit statistically significant differences in climatic niche occupancy, as evidenced by spatial autocorrelation-corrected statistical analyses (External Supplementary Dataset 4^41^). The modelling was based on six key climatic variables: annual mean temperature (Bio1), annual precipitation (Bio12), precipitation of the driest month (Bio14), precipitation seasonality (Bio15), precipitation of the warmest quarter (Bio18), and precipitation of the coldest quarter (Bio19). The most influential variables separating the clusters were Bio1, Bio12, Bio14, and Bio19 (Supplementary Data 14). Specifically, the xeromorphic Cluster III is adapted to the most arid environments; it is statistically distinct from Clusters I and II across all variables (Supplementary Fig. 5), consistently occupying habitats defined by low annual precipitation (Bio12). Its niche suitability peaks where precipitation in the driest month (Bio14) is below 20 mm (Supplementary Fig. 6), reinforcing its specialisation. In contrast, the mesomorphic Cluster I and montane Cluster II do not differ significantly in overall annual temperature or precipitation. Instead, their niches are partitioned by seasonality. Cluster I thrives in environments with the least seasonal precipitation, whereas Cluster II occupies a unique climatic niche restricted to monsoonal regimes with low winter precipitation (Bio19 < 100 mm; Supplementary Fig. 6). This specific environmental parameter strongly distinguishes the habitat of Cluster II from those of both other clusters (*p* < 0.001 for all pairwise comparisons; Supplementary Fig. 5).

Projections into interglacial periods (present and 11 Kya, MIS1; 130 Kya, MIS5e; 787 Kya, MIS19c) demonstrated similar spatial extents for Clusters I and III across most of the period modelled. However, climatic conditions during the Last Glacial Maximum (LGM, 21 Kya) largely affected Cluster I, leading to increasingly fragmented and restricted habitats across Eurasia (Fig. 6c). This aligns with its declining demographic trajectory during late MIS4 (ca. 57 Kya; Fig. 5d) and a relatively low *N*e of 109,000 during the LGM and subsequent habitat recovery post-LGM (Fig. 5d; 6d, e). Cluster III, on the other hand, exhibited only moderate contraction during the LGM (Fig. 6m), which is also reflected by its population dynamics (Fig. 5d) even as ice sheets advanced. Notably, current range overlaps between Clusters I and III in temperate zones, and Clusters II and III in the subtropics, likely represent recent competitive or complementary niche dynamics leading to secondary contacts, supporting the hybridisation and introgression events detected by genetic analyses (Figs. 2, 3, 5).

Cluster II, associated with strongly monsoonal climates, shrank its suitable range progressively from 11 Kya to the present (Fig. 6i, j). This recent decline in suitable areas parallels its effective population size reduction observed in the Stairway Plot 2 (Fig. 5d), highlighting climate-driven demographic stress. Despite this recent contraction, Cluster II maintained habitat connectivity during the LGM (Fig. 6h), possibly due to refugia in less ice-affected regions. Furthermore, range overlaps of Cluster II are more pronounced with Cluster III than Cluster I, even during the LGM. At that time, these overlaps were limited to small patches in the Tibetan Plateau and the Russian Far East (Fig. 6r). These historical overlaps provided opportunities for gene flow, consistent with the GADMA2 analysis, which inferred bidirectional gene flow between Clusters II and III commencing during mid-MIS8 (Fig. 5b).

Although the available time periods for palaeodistribution models did not allow us to detect niche trends explaining all demographical fluctuations within clusters in detail, integrating phylogenetic, demographic, and palaeodistribution data highlights key evolutionary events. Specifically, the projection of shared habitats across the studied timelines, particularly between the two oldest Clusters (I and II), reveals a consistent overlap centred on the Tibetan Plateau and the Russian Far East (Fig. 6p). This observation supports these regions as potential ancestral divergence zones and critical refugia, particularly during provisional warming periods within the 820–370 Kya timeframe suggested by SNAPP for the earliest divergence (Fig. 5e).

Synthesising these findings, the ancestral lineage of Clusters II and III subsequently diverged, leading to their separation approximately 410 Kya (95% HPD: 580–240 Kya, Fig. 5e). These ancient divergence events, occurring in regions including the Tibetan Plateau and the Russian Far East, underscore their roles as long-term refugia (Fig. 6q–t). These refugia would have facilitated persistence during glacial maxima, with subsequent post-glacial warming enabling range expansion and diversification for the resulting lineages, thereby shaping their distinct demographic trajectories (Fig. 5).

### Cytological variation

Based on the compilation of published data (Supplementary Data 15), section *Stenopoa* exhibits substantial cytotypic diversity that does not strictly correspond to the genetic clusters. Cluster I includes taxa with reported chromosome numbers ranging from 2n = 14 to 84; Cluster II ranges from 14 to 74; and Cluster III ranges from 18 to ca. 100. While exact multiples of the basic number (x = 7) are common (e.g., 2n = 28, 42, 56), counts deviating from these multiples are frequently recorded. For instance, Cluster I includes dysploid counts (e.g., 2n = 26, 33, 50) and B-chromosomes (2n = 42 + 0 − 3B). Cluster II includes mostly even dysploid numbers such as 2n = 46, 60, and 74; however, mixoploidy is also recorded (e.g., in *P. faberi* with 2n = 28 and 35). Cluster III displays highly variable counts, including 2n = 18, 38, and 50. Similarly, the taxa identified as genetically admixed display a comparable range (2n = 14 to 84), including aneuploids (e.g., 2n = 38, 49 ± 1). This widespread variation suggests a prevalence of dysploidy and/or chromosomal instability associated with facultative apomixis and hybridisation throughout the section.

## Discussion

In this study, we integrated genomic, morphological, and palaeoclimatic data to investigate the evolutionary history of bluegrasses in the section *Stenopoa*, revealing how Pleistocene climatic fluctuations shaped their diversification and expansion across Eurasia. Critically, our high-throughput sequencing approach advances prior work limited to morphology^34,47,48^ or small-scale dominant markers^49^, providing the first comprehensive reconstruction of evolutionary dynamics in this section. These findings highlight the complex evolutionary history of this polyploid clade, shaped by climate change and adaptive radiation in this ecologically significant grass lineage.

Our analyses revealed three well-supported genetic groups within *Stenopoa*, aligned with their ecological preferences (Fig. 1). Cluster I typically comprises taller, mesomorphic plants from humid lowland meadows and forests. Cluster II includes taxa adapted to a wider range of montane conditions within monsoonal regimes, from humid coniferous forests to cold alpine deserts, exhibiting considerable morphological variation from taller, softer forms to dwarf, hardy plants. Cluster III encompasses taxa ranging from moderately to extremely xeromorphic, characteristic of steppes, dry slopes, and high-mountain cold grasslands, generally exhibiting more rigid structures and morphological traits associated with aridity tolerance (e.g., reduced stature, narrow leaves; Figs. 1, 4). These distinctions underscore an ecological differentiation that parallels the genetic clustering.

Notably, morphological data distinguished only two relatively distinct morphotypes (Cluster I vs. Clusters II and III; Fig. 4). This strictly aligns with the primary, objective genetic division supported by the Evanno test (*K* = 2), demonstrating that the deepest genomic split directly corroborates the primary morphological divergence. Furthermore, the subdivision into three genetic clusters, justified by distinct geographical and ecological niches, suggests that genetic differentiation between Clusters II and III may precede significant morphological divergence, a characteristic feature of early-stage ecological speciation. This discordance also aligns with the reticulate phylogeny observed in the section (Fig. 2), where groups differentiated by ecological factors remain interconnected, forming a species continuum rather than distinct groups with strong reproductive barriers^50^. The extensive morphological variation within clusters, likely amplified by hybridisation and mixed-ploidy levels, may have favoured the adaptive radiation documented in bluegrasses^18^. Our findings align with previous studies across several plant families, including Asteraceae^51^, Rosaceae^52^, and Gentianaceae^53^, suggesting that hybridisation and subsequent polyploidy generate genetic diversity, leading to species diversification in changing environments. In such contexts, diverse ecological niches favour new genetic combinations, thereby facilitating adaptive radiation. In *Stenopoa*, this process appears to have been particularly important for adaptation to the varied environmental conditions across Eurasia, allowing colonisation of habitats ranging from humid lowlands to harsh alpine zones.

Importantly, our findings demonstrate the direct link between the morphological traits driving phenotypic divergence and the climatic variables defining the clusters’ ecological niches. The LDA identified panicle length and flag leaf blade length as the most critical traits separating the lineages. These traits are functionally significant: larger panicles and leaves, as seen in the mesomorphic Cluster I, maximise photosynthetic capacity and reproductive output in resource-rich, humid environments where competition for light is high. Conversely, the more compact stature and smaller leaf surfaces that characterise the xeromorphic Cluster III are classic adaptations to conserve water in arid environments^54^, which our niche modelling identified as its core habitat (defined by low annual precipitation and low precipitation in the driest month). The intermediate morphology of Cluster II (Figs. 1, 4), resulting in its lower classification accuracy in the LDA, likely reflects adaptation to a much broader range of ecological conditions, from humid montane forests to cold alpine deserts, compared to the more specialised Clusters I and III. Such ecological generalism would select for a more plastic phenotype capable of thriving across a heterogeneous landscape, rather than a single, fixed morphotype. Furthermore, its subtle distinction may represent a more nuanced adaptive strategy. For instance, its longer upper glumes could enhance wind dispersal of seeds, prioritising reproductive efficiency in the shorter growing seasons typical of its cold, seasonal montane habitats, while its significantly longer flag leaf ligules may act as a physical barrier preventing water accumulation within the leaf sheath during heavy monsoonal rains. This tight coupling between form and environment provides strong evidence that morphological evolution in this group is a direct adaptive response to the distinct climatic selection pressures, particularly water availability, that have driven the diversification of the *Stenopoa* section across Eurasia.

While determining the precise ploidy of historical samples was precluded by the nature of the herbarium material, the synthesis of published chromosome counts suggests that the genetic clusters identified here represent distinct evolutionary lineages rather than simple cytotypes. For instance, Cluster I includes taxa with counts ranging from 2n = 14 to 84, Cluster II from 14 to 74, and Cluster III from 18 to ca. 100 (Supplementary Data 15). This substantial variation within genetic groups indicates that the genomic structure is driven primarily by phylogenetic divergence and ecological isolation, with polyploidisation occurring dynamically within these lineages. The prevalence of high even chromosome numbers (e.g., 84 in *P. palustris*) may suggest repeated allopolyploidisation events, similar to those in *Senecio*^55^, *Triticum*^56^, and *Festuca*^57^, or autopolyploidisation similar to *Saccharum*^58^. While distinguishing between allopolyploidy and autopolyploidy remains exceptionally difficult in taxonomically complex non-model groups such as *Stenopoa*, the presence of counts deviating from exact multiples of the basic number (x = 7^59,60^) indicates that other mechanisms, such as aneuploidy, dysploidy, and the formation of unreduced gametes, also play a significant role. These processes, frequently associated with facultative apomixis in *Poa*^61,62^, likely contribute to the high cytotypic complexity observed across the section. Such genomic plasticity provides the genetic redundancy required for these lineages to colonise and persist in the diverse and often harsh environments of Eurasia, while also maintaining a high potential for cross-ploidy hybridisation^35^. This diversity can arise in primary contact zones or through migration after allopatric divergence^63^. Our phylogeographic and molecular evidence points towards secondary contact as a key factor for Clusters I and III, while Clusters II and III likely maintained connectivity over time, suggesting a greater influence of sympatric processes. Future studies utilising direct chromosome counting are needed to verify these hypotheses. Specifically, this clade represents an ideal system to investigate the ratio of unreduced gamete formation in stressful environments and to explore the potential directionality of hybridisation (e.g., introgression towards higher ploidy parents), as recently highlighted by Brown et al.^64^.

The evolutionary scenarios tested with GADMA2 indicate that the common ancestor of *Stenopoa* was characterised by a relatively small effective population size that is consistent with a scenario where the lineage persisted in restricted refugia during the harsh glacial conditions of MIS14 or earlier (Figs. 5b, 6p). From this ancestral population, subsequent expansion during a warmer interglacial likely gave rise to the lowland-adapted Cluster I and the lineage that would later specialise in colder climates (Clusters II and III). As hypothesised, Cluster II then likely originated via niche partitioning. Our palaeoclimatic projections identify the Russian Far East and the Tibetan Plateau as plausible areas for these initial divergences, where the niches of Clusters I and II overlapped (Fig. 6p). These topographically complex regions with steep climatic gradients could have facilitated ecological speciation. The subsequent divergence of Clusters II and III (ca. 410 Kya, Fig. 5e) further underscores the role of these regions as long-term refugia (Fig. 6q–t), facilitating persistence during glacial maxima and enabling post-glacial expansion and diversification. Given the exceptional variation in chromosome numbers in this group (Supplementary Data 15) and a likely origin from small ancestral populations, WGDs, including allopolyploidy, would have been particularly advantageous, providing the genetic redundancy and novel variants needed to buffer against extreme environmental regimes. This aligns with the broader pattern observed in plants, where polyploids often exhibit superior colonisation abilities in formerly glaciated or unstable landscapes compared to their diploid progenitors^5,65^. Such ecological success is frequently attributed to enhanced heterozygosity and adaptive trait innovation, which stabilise populations under environmental stress^66^.

The ecological niche models revealed differential responses to glacial periods: Clusters II and III maintained better habitat connectivity, suggesting superior adaptation to cold, while the range of Cluster I became highly fragmented during the LGM (Fig. 6c, h, m). These climatic oscillations likely drove cycles of isolation and reconnection, fostering allopatric divergence followed by secondary contact and gene flow. This pattern mirrors the evolutionary history of New World and Australasian *Poa* lineages, where Giussani et al.^36^ identified habitat fragmentation, aridisation, and cooler climates during the Pleistocene as the primary drivers of speciation. Our findings confirm that these global climatic shifts acted as a universal catalyst for *Poa* diversification, shaping the *Stenopoa* section in Eurasia through the same mechanisms of vicariance and adaptation that drove radiations in the Southern Hemisphere. The differential climatic resilience observed, with Cluster III showing greater flexibility and Cluster II greater specialisation, also offers insights into potential responses to ongoing climate change. In addition, while our data point to the Russian Far East and Tibetan Plateau as plausible divergence hotspots (Fig. 6p), the precise origin of the section remains uncertain due to the lack of unambiguous fossils. Previous research suggests Southwest Asia as a more probable source for bluegrasses, while North Asia is considered less likely^19^. As our sampling included only three Southwest Asian individuals (Türkiye; Supplementary Data 1), expanding biogeographical sampling in future studies would help clarify the origin of ancestral populations in *Stenopoa*.

Importantly, the time-calibrated phylogeny inferred by SNAPP identified *P. palustris* (Cluster I) and *P. orinosa* Keng (Cluster II) as early-diverging lineages. Both species retain diploid cytotypes (Supplementary Data 15). The source populations for these published diploid counts, such as lowland Ukraine for *P. palustris* and montane northeastern China for *P. orinosa*, align with our ecological niche models but lie outside the core ancestral divergence zones identified in our study. Because ploidy data from these critical refugial areas are currently lacking, future botanical expeditions should target the Russian Far East and the Tibetan Plateau to search for persisting diploid populations. Confirming the presence of diploids in these regions would provide the strongest independent test of our refugial hypothesis and identify prime candidates for future genome sequencing projects. However, locating these diploid populations remains a significant challenge. As noted by Soreng et al.^13^, diploids are rare in the genus, comprising only ca. 13–15% of species. These potential diploid lineages could provide crucial insights into the genomic changes associated with polyploidisation and adaptation in *Stenopoa*. So far, several whole bluegrass genomes have been released to better disclose the evolutionary history of some species^37–40^. For instance, it has been evidenced that the extraordinary phenotypic plasticity of the allotetraploid *P. annua* is explained by the divergent evolution of its diploid ancestors^40^. Besides, the genomes of *P. pratensis* L. revealed substantial variation in genome size among different populations, reflecting a complex infraspecific structure where subspecies genetically and morphologically grade into one another^38^. As it was recently proposed for the genus *Senecio*, new molecular information, such as highly contiguous (ideally chromosome-level) annotated genomes alongside transcriptomics data, may shed light on the interaction between phenotypic plasticity and natural selection, and how ancestral populations specialised to new environments^67^.

Several limitations of our study should be acknowledged. First, while our sampling covered a substantial portion of *Stenopoa*’s range, additional sampling in underrepresented regions could refine our understanding of its evolutionary history. Furthermore, the inherent phenotypic ambiguity within *Stenopoa*, arising from hybridisation and observed mixed-ploidy, precluded a detailed taxonomic revision in this study. Such a revision would necessitate additional sample collection and further analyses, including morphological (e.g., scanning electron microscopy for microtrait differences), cytogenetic, and advanced molecular techniques. Consequently, our genomic analysis revealed significant mismatches with traditional taxonomic classifications (80 of the 373 individuals were reassigned; Supplementary Data 3). For instance, taxa such as *P. litvinoviana* were recovered as polyphyletic (appearing in multiple unrelated genetic groups; Fig. 3), suggesting that this taxon as currently defined may represent a morphological ecotype convergent across different lineages rather than a single evolutionary unit. Similarly, the robust grouping of taxonomically distinct species like *P. nemoralis* and *P. ×intricata* within the *P. palustris*-dominated Cluster I indicates a cohesive genomic background that is often obscured by morphological variation. These inconsistencies highlight the need for further investigation to elucidate their true nature and phylogenetic position within *Poa*. Second, the number of SNPs retrieved using DArTseq is relatively low (Supplementary Data 2) compared to projects utilising whole genomes and resequencing. Specifically, higher-density datasets are crucial for disentangling the confounding signals of recent introgression versus incomplete lineage sorting, a common challenge in reticulate complexes. Additionally, while the subsampling of markers to a biallelic format mitigates ploidy biases, access to phased haplotypes would be necessary to fully resolve fine-scale demographic parameters in this complex polyploid system. Consequently, our estimates of population dynamics and evolutionary scenarios should be treated with caution, representing broad evolutionary trends rather than precise quantitative values. Third, future research should focus on investigating the genomic and ecophysiological basis of adaptation in *Stenopoa*, particularly the genetic mechanisms underlying morphological and ecological differentiation between clusters. As formal testing of phenotypic plasticity is currently lacking for this section, experimental studies involving common garden cultivation would complement our ecological niche models and provide mechanistic insights into their adaptive capacities. Additionally, incorporating precise cytological data (distinguishing diploids, polyploids, and aneuploids) into this integrative framework is essential to fully disentangle the specific influence of genome duplication and dysploidy on evolutionary responses to climate change across diverse plant lineages. Lastly, the lack of reliable fossils introduces two distinct limitations. Our divergence‑time estimates are anchored by a single calibration point derived from a previous molecular study^18^ rather than direct fossil evidence. Future work incorporating fossil‑calibrated phylogenies of close relatives (e.g., within Pooideae) and employing independent mutation‑rate estimates would provide more robust temporal frameworks for understanding the evolutionary history of *Stenopoa*. Moreover, as fossil data are lacking, the resolution of palaeoclimatic data relies here solely on current distributions, potentially affecting the accuracy of niche projections for the oldest time periods.

In conclusion, our study demonstrates how the integration of genomic, morphological, and palaeoclimatic data can reveal the complex evolutionary history of a taxonomically challenging plant group. Our findings reveal that Pleistocene climatic fluctuations drove the adaptive radiation of *Stenopoa* across Eurasia, with lineages evolving distinct morphological adaptations (e.g., reduced stature and panicle size) in response to the specific pressures of cold and arid ecological niches. While our data highlights the incongruence between traditional taxonomy and genetic structure, it also reveals that this diversification occurred within a complex evolutionary background of hybridisation and widespread cytotypic variation. Consequently, determining the precise role of polyploidy in providing genetic redundancy under environmental stress, alongside identifying the genomic basis of these morphological adaptations, remain critical aims for future research.

By linking historical demographic responses to specific climatic variables (annual mean temperature, precipitation seasonality, and annual precipitation) and identifying the functional traits (flag leaf blade length and panicle length) that distinguish lineages with contrasting ecological tolerances, our study provides a framework for projecting how cold-adapted polyploid flora may respond to ongoing climate change. Specifically, the differential climate resilience documented here, with the xeromorphic Cluster III exhibiting broader niche flexibility and the monsoonal-adapted Cluster II showing greater specialisation and recent range contraction, offers empirical reference points for anticipating lineage-specific trajectories under projected warming and altered precipitation regimes. For instance, continued aridification in Central Asian steppes may favour the expansion of Cluster III lineages, while increasingly unpredictable monsoonal patterns could further stress the already declining Cluster II. Such trajectories are consistent with the extinction debts and delayed range shifts widely documented in other climate-threatened mountain floras^68,69^, including those recently identified in the same Central Asian high mountain systems^70^. These predictions, however, assume that the climatic niches inferred from present and palaeodistribution data remain stable under novel future conditions, a key assumption warranting experimental testing. Future research should evaluate these projections by integrating our framework with high-resolution future climate scenarios, experimental assessments of physiological thresholds, and long-term monitoring of population dynamics at range edges. More broadly, the integrative approach developed here, combining genomic structure, functional trait analysis, and palaeoclimatic niche reconstruction, offers a robust template for assessing climate change vulnerability in other taxonomically complex, polyploid-rich plant groups where traditional species-based assessments are impractical.

## Materials and methods

### Plant material and sampling strategy

Taxon sampling included 373 individuals of the section *Stenopoa* that had been collected in Eurasia over 47 years between 1975 and 2022. Given the reliance on historical herbarium vouchers to maximise geographic extent, sampling was not population-based in the traditional sense; most collection sites are represented by single individuals. Therefore, for downstream analyses, “populations” were defined *a posteriori* based on the robust genetic clusters resolved by the joint application of STRUCTURE, Principal Component Analysis (PCA), and Neighbor-Net analyses. All vouchers are deposited in the herbarium of Tomsk State University (TK) and listed in Supplementary Data 1.

### DNA extraction, amplification and DArT sequencing

The following procedures, including DArTseq genome complexity reduction and DNA genotyping^71^, were performed by Diversity Arrays Technology Pty Ltd (Canberra, Australia). Genomic DNA was isolated from desiccated leaf tissue utilising the NucleoMag kit (Macherey-Nagel, Germany). Lysis was initiated by adding 50 µL of T1 Buffer and 6.25 µL of proteinase K to each sample. To guarantee full submersion of the plant material, plates underwent a brief centrifugation (1000 rpm for 30–60 s). Following an overnight incubation at 60 °C, the digested samples were spun down at 3000 rpm for 10 min, allowing the clear supernatant to be collected into fresh deep-well plates. For DNA binding, 90 µL of MB2 buffer containing 6 µL of NucleoMag B-beads was added under constant agitation to maintain bead suspension. Subsequent purification phases, including all washing and elution procedures, were executed automatically using a 96-tip head on a Tecan T100 robotic platform (Tecan, Switzerland). For library preparation, the samples were ligated with adaptors possessing complementary overhangs for the restriction enzymes *PstI* and *HpaII*^72^. These adaptors were customised to contain a variable-length “staggered” barcode, a sequencing primer binding site, and the required Illumina flowcell attachment sequences, adopting an architecture analogous to that described by Elshire et al.^73^. The reverse adaptor was designed to incorporate both the necessary flowcell attachment sequence and an *HpaII*-compatible overhang. To selectively enrich for “mixed fragments”, a 30-cycle PCR amplification was performed. The thermocycling profile was initiated with a 1-minute denaturation at 94 °C, followed by 30 cycles of 94 °C for 20 seconds, 58 °C for 30 s, and 72 °C for 45 s, concluding with a final extension step at 72 °C for 7 min. This cycle number was selected to ensure sufficient library yield from historical herbarium specimens. Library preparation included a size selection step targeting fragments in the 100–300 bp range following DArT’s internal protocols. After PCR, equimolar amounts of amplified product were bulked and sequenced on Illumina NovaSeq 6000 or NovaSeq X platforms (Illumina, USA) via single-end reads (83 bp or 138 bp, respectively).

### Variant calling and filtering

Sequences generated from each lane were processed using proprietary DArT analytical pipelines developed by Diversity Arrays Technology Pty Ltd (Canberra, Australia). For marker calling, raw reads were uniformly truncated to approximately 69 bp. The primary pipeline eliminated sub-standard sequences; to guarantee accurate sample demultiplexing (“barcode split”), filtering parameters were intentionally stricter for the barcode regions than for the remaining read. Specifically, raw reads were filtered requiring a minimum Phred score of 30 across 75% of the bases in the barcode region, and a minimum Phred score of 10 across 50% of the entire read. Marker calling relied exclusively on unique sequences per individual. Identical reads were aggregated into collapsed files (“fastqcoll”), which subsequently underwent an algorithmic “grooming” process where high-coverage collapsed tags served as templates to correct low-quality bases within singleton tags. These corrected fastqcoll files were fed into the DArTsoft14 secondary pipeline to execute DArT PL’s proprietary algorithms for both SilicoDArT (restriction fragment presence/absence) and SNP marker extraction. Initially, a proprietary C + + algorithm (DArT PL) clustered the sequencing tags across all libraries using a distance threshold of 3 (equivalent to approximately 95% sequence identity). These clusters were subsequently parsed into distinct SNP loci by enforcing a minimum allelic balance of 5. To effectively distinguish genuine allelic variants from paralogous artifacts, the pipeline incorporated selection criteria empirically derived from evaluating Mendelian allele distributions across roughly 1000 controlled cross populations. Finally, marker quality and error rates were validated by processing multiple technical replicates (from the initial DNA stage through to allele scoring) with scoring consistency serving as the primary metric for marker retention. Given the mixed-ploidy nature of the dataset and the lack of a reference genome, SNPs were treated as “mosaic diploids” (biallelic format) to facilitate standard population genomic analyses, following established protocols for DArTseq data in complex polyploid systems^70^. For the downstream analyses, we applied co-dominant SNP markers processed in R v.4.3.1^74^ via the R packages dartR v.2.9.7, dartRverse v.1.0.2 and dartR.popgen v.1.0.0^75,76^ with the following parameters: (1) a scoring reproducibility of 100%, (2) SNP loci with read depth < 5 or > 50 were removed, (3) at least 70% loci called (the respective DNA fragment had been identified in greater than 70% of all individuals), (4) monomorphic loci were removed, (5) to ensure locus independence, SNPs that shared secondaries (had more than one sequence tag represented in the dataset) were randomly filtered out to keep only one random sequence tag.

### Genetic structure and molecular dating

Firstly, the genetic structure of all 373 specimens was analysed independently of morphological or ecological data using the dataset with a 70% call rate by applying: (1) Unweighted Pair Group Method with Arithmetic Mean (UPGMA), (2) PCA, (3) STRUCTURE v.2.3.4^77^, and (4) Neighbor-Net via SplitsTree v.6.3.32^78^. The UPGMA cluster analysis based on the Hamming Distance with 1000 bootstrap replicates was performed in the R package poppr v.2.9.4^79^, while the final tree was visualised via iTOL v.6.9.1^80^. The STRUCTURE analysis was implemented via StrAuto v.1.0^81^ using the admixture model with correlated allele frequencies and without a priori population information (popflag = False). We performed ten replicate runs for each number of clusters (*K*) from 1 to 15 with a burn-in of 10,000 iterations followed by 100,000 MCMC iterations. MCMC convergence was verified by visual inspection of parameter trace plots to ensure that the Alpha (*α*) parameter reached stationarity. The optimal *K*-value was identified based on Evanno’s method of delta *K* statistics^82^ and assessed via Structure Harvester^83^. The R package pophelperShiny v.2.1.1^84^ was used to align replicate runs and visualise the output matrices to assess cluster stability and confirm the consistency of individual assignments across independent runs. Following this validation, replicates were merged to generate the final consensus matrix.

Next, due to the extensive hybridisation detected, we constructed a phylogenetic network using the Neighbor-Net method^85^ based on the Hamming Distance and default parameters implemented in SplitsTree. Additionally, the PCA based on the Euclidean distance matrix was performed using dartR and visualised with ggplot2 v.3.4.3^86^. Further, to measure the differentiation between the pure genetic clusters (the membership coefficient ≥ 0.99) established with STRUCTURE we calculated the fixation index (*F*_*ST*_), the number of fixed and private alleles, as well as the absolute allele frequency difference (*AFD*^87^) via dartR. Subsequently, to define robust evolutionary units for downstream phylogenetic dating and morphological characterisation, we performed a fine-scale investigation of genomic structure by analysing the pure individuals within each cluster separately (Supplementary Fig. 1) using a combination of STRUCTURE, Neighbor-Net, and PCA with the above-mentioned parameters. This screening aimed to identify cohesive genetic sub-groups and exclude unstable lineages or potential cryptic hybrids that could confound phylogenetic inference or blur morphological differentiation. The final set of genetically pure taxa defined by this process was used to infer a time-calibrated species-tree under the multispecies coalescent model using the SNAPP package v.1.5.1^88^ through BEAST v.2.6.3^89^. We applied one time calibration, setting a log-normal distribution with a mean of 0.61 Mya and a standard deviation of 0.20 for the initial split in the section *Stenopoa*, following Hoffmann et al.^18^ (*Poa* IV, the basal node age of 20 Mya). The analysis was performed independently three times, with 1.25 million MCMC generations for each run. Sufficient sampling was assessed using Tracer v.1.7.1^90^, verifying the combined log file regarding Effective Sample Size (ESS) values. As all ESSs exceeded 200, we combined tree files, discarding the first 10% as burn-in from each run. The final maximum clade credibility tree was summarised in TreeAnnotator v.2.6.3 (a part of the BEAST package). A pattern across all posterior trees was visualised via DensiTree v.2.01^91^, while FigTree v.1.4.4^92^ was used to inspect the Bayesian posterior probabilities and the 95% credibility intervals of the final tree.

### Morphometric analysis

A total of 52 genetically pure individuals (Cluster I: *n* = 13; Cluster II: *n* = 10; Cluster III: *n* = 29) were examined under a light microscope MBS-10 (LOMO, Russia) across 21 of the most informative quantitative characters commonly used in taxonomic descriptions of *Poa* (Supplementary Data 6; External Supplementary Dataset 2^41^). Univariate and multivariate normality tests were performed using R package MVN v.5.9^93^, with log transformation of non-normal variables to achieve normality (Supplementary Fig. 7). A subsequent PCA with standardised data was conducted using R package FactoMineR v.2.8^94^ to characterise the variation within the taxa and to extract the variables that best identified them. R packages factoextra v.1.0.7^95^ and plotly v.4.10.2^96^ were used to visualise the first two and the first three principal components, respectively. To assess statistical significance of differences in individual traits among the three genetic clusters, we performed a one-way analysis of variance (ANOVA) for normally distributed variables. If the ANOVA indicated a significant overall difference (*p* < 0.05), we conducted post-hoc pairwise comparisons using Tukey’s Honestly Significant Difference (HSD) test to identify which specific cluster pairs differed significantly (adjusted *p* < 0.05). For variables that did not meet normality assumptions, we used the non-parametric Kruskal-Wallis rank-sum test. If a significant overall difference was detected (*p* < 0.05), pairwise comparisons were performed using Dunn’s test with Bonferroni correction for multiple comparisons to identify significantly different cluster pairs (adjusted *p* < 0.05). Additionally, to evaluate distributional relationships between each response variable and the studied taxa, notch plots and interactive box plots were created using R packages ggplot2 v.3.4.3 and plotly v.4.10.2, respectively.

To further evaluate morphological differentiation among the three genetic clusters, we conducted a Linear Discriminant Analysis (LDA; External Supplementary Dataset 2^41^). Prior to the LDA, traits were inspected for collinearity using Spearman’s rank correlation. Highly correlated variables (*r* > 0.90) were reviewed, and two traits (L2I and LLPB) were removed to reduce multicollinearity. This reduced dataset was then standardised (mean = 0, SD = 1) before applying Recursive Feature Elimination (RFE) with 10-fold cross-validation to select the most relevant class separation traits via R package caret v.6.0–94^97^. The final LDA model was constructed using the RFE-selected variables with R package MASS v.7.3-60^98^. Model performance was evaluated using resubstitution accuracy, leave-one-out cross-validation (LOOCV) accuracy, and a permutation-based cross-validation approach (100 iterations, 80% training and 20% testing split), where RFE was re-applied within each training fold to provide an unbiased estimate of predictive accuracy on unseen data.

### Demographic reconstruction and evolutionary scenarios testing

Two composite likelihood methods based on the coalescent model, Stairway Plot v.2.1.1^43^ and GADMA v.2.0.2^42^ with the moments engine^99^, were employed to estimate historical effective population sizes of the three genetic Clusters (I, II, and III) using folded site frequency spectra (SFS) derived from SNPs (Supplementary Data 2). Filtering criteria comprised the original dataset (70% of loci called) to maximise the number of SNPs and a more stringent protocol (95% of loci called) to maximise SNP quality.

Analyses with Stairway Plot used 200 bootstraps, a two-year generation time, and a mutation rate of 6 × 10^−9^ following De La Torre et al.^100^. The total length of the sampled sequences was estimated as the total number of loci multiplied by the trimmed sequence length (69 bp).

For the GADMA analyses, demographic models were defined through epochs representing periods during which populations maintained a constant size or underwent exponential/linear growth at a fixed rate, with migration rates constrained to remain constant within each epoch. The algorithm was configured to automatically select the most suitable population size dynamic for each epoch and population. Consistent with our dual-dataset rationale, the strategy for GADMA was to first select the best-fitting model for each analysis using the Akaike Information Criterion (AIC) on the 95% dataset. Subsequently, the parameters of that chosen model were re-estimated using the 70% dataset to obtain the final reported values. Confidence intervals for the parameters were derived from 100 bootstrapped datasets generated for both datasets (Supplementary Data 10) using the Godambe information matrix approach according to Coffman et al.^101^.

The demographic history of these three genetic clusters was inferred using a hierarchical approach. First, the history of each pair of Clusters was inferred by testing two models: (1) an isolation with migration model (epoch-based with flexible population size dynamics and continuous migration); and (2) a secondary contact model, in which population size dynamics were fixed to those inferred from the first model, and migration was constrained to begin after an initial period of isolation. Second, the joint history of all three Clusters was investigated by comparing three distinct models: (1) a flexible epoch-based model allowing for continuous gene flow between all cluster pairs; this model is similar to the pairwise isolation with migration model but allows for the joint inference of all three clusters’ dynamics; (2) a simpler model where population size dynamics were fixed based on the results of the first three-cluster model, and times of migration events were informed by the pairwise inferences; therefore, this model was a combination of isolation with migration and secondary contact models for different pairs; and (3) an admixture model designed to explicitly test the formation of Cluster III via an admixture event between Clusters I and II, with subsequent gene flow patterns mirroring those in the second model. In one case, where the AIC scores from the two datasets supported different models, the history supported by the 70% dataset was found to be a credible alternative (Supplementary Fig. 3; External Supplementary Dataset 3^41^). Demographic history visualisation was generated using Demes v.0.2.3^102^.

### Palaeodistribution prediction

The occurrence data was derived from the 199 individuals assigned to the three “pure” genetic Clusters (I, II, and III) identified in the study (Supplementary Data 1); genetically admixed individuals were excluded to ensure the models represented the distinct ecological niches of each lineage. Prior to modelling, records were cleaned to remove spatial duplicates within each genetic cluster separately. Subsequently, spatial thinning was performed to minimise autocorrelation in the occurrence data using the R package spThin v.0.2.0^103^, reducing redundant records within a 1 km² grid. This process removed 56 redundant entries. In total, the final dataset included 143 spatially unique occurrences: 51 for Cluster I, 21 for Cluster II, and 71 for Cluster III (Supplementary Data 11).

Climatic variables for the present were sourced from the WorldClim database v.2.1^104^ that provides data on average climatic conditions for the period between 1970 and 2000. To assess species distribution across historical climate events, the past climatic variables for the Last Glacial Maximum (LGM; ~22–18 Kya) and the Last Interglacial (LIG; ~130 Kya) were sourced from the WorldClim database v.1.4^105^, while data for the early Holocene (11.7–8.3 Kya) and the Marine Isotope Stage 19 (MIS19; ~787 Kya) were obtained from the PaleoClim database v.1.0^106,107^.

In addition to the primary data sources mentioned above, 14 bioclimatic variables (Supplementary Data 12) available for all time periods were standardised and resampled to a uniform spatial resolution of 2.5 arc minutes (~5 km) using the R package raster v.3.6-26^108^ to ensure consistency. To address multicollinearity among the variables, the variance inflation factor (VIF) and Pearson’s correlation coefficient (r) were computed. Potential multicollinearity issues were eliminated using the R package usdm v.2.1-7^109^. Climatic variables with a VIF greater than 10 and *r *> 0.8 were filtered out, resulting in the selection of six key variables (detailed in the Results section and Supplementary Data 14). Subsequent species distribution modelling (SDM) was performed using the R package dismo v.1.3-14^110^.

A maximum entropy (MaxEnt) algorithm v.3.4.4^44^ was employed for modelling using the prepared environmental and occurrence data. The dataset was divided into two subsets: 90% for training and 10% for testing, aimed at evaluating model performance. The accuracy of the models was measured using the Area Under the Curve (AUC) of the Receiver Operating Characteristic (ROC) curve^45^ and the True Skill Statistic^46^ (TSS). AUC values exceeding 0.8 and TSS values greater than 0.7 were deemed indicative of good model performance (Supplementary Data 13). The final models generated probabilistic maps indicating suitable habitats of the three genetic clusters. These results were evaluated in the context of the ecological preferences and environmental conditions documented in major regional floras, including the Flora of the USSR^10^, the Flora of China^11^, and taxonomic revisions of the section^28^. The modelled habitats aligned closely with the independent descriptions provided in these floras.

To test for significant differences in environmental variables among the three genetic clusters, we accounted for non-independence of samples due to spatial autocorrelation using generalised least squares models implemented in the R package nlme v.3.1.162^111^. For each environmental variable, we modelled an exponential spatial correlation structure based on sample geographic coordinates and allowed for heterogeneous variances among clusters using the varIdent function (External Supplementary Dataset 4^41^). We compared models with and without a nugget term (a parameter of the spatial covariance structure used to account for residual spatial autocorrelation) using the AIC to select the best-fitting structure. In the final model, the statistical significance of the fixed effect of the genetic cluster was assessed with ANOVA. Post-hoc pairwise comparisons between clusters were then conducted using estimated marginal means with the R package emmeans v.1.8.8^112^ and Tukey’s *p*-value adjustment. The final notch plots were plotted according to the pipeline described previously in the Methods (Morphological structure section).

### Cytological data compilation

As direct estimation of ploidy was precluded by the exclusive use of dried herbarium material (yielding degraded nuclei unsuitable for flow cytometry) and the insufficient read depth of sequencing data for in silico dose calling, we compiled published chromosome number data for *Poa* sect. *Stenopoa* from available literature. This involved cross-referencing records from historical databases, systematic revisions, and personal communications with the *Poa* expert Dr Robert J. Soreng (Supplementary Data 15).

### Statistics and reproducibility

Sample sizes were determined by the availability of viable historical herbarium specimens, resulting in a final dataset of 373 genotyped individuals. No statistical methods were used to predetermine sample size. To ensure robust genetic clustering, analyses (e.g., STRUCTURE, SNAPP) were performed with multiple independent replicate runs to verify MCMC convergence and cluster stability. For morphological analyses, data distributions were evaluated for normality. Subsequently, statistical significance among genetic clusters was assessed using either parametric (one-way ANOVA followed by Tukey’s HSD) or non-parametric (Kruskal-Wallis followed by Dunn’s test with Bonferroni correction) methods, with significance thresholds strictly defined at *p* < 0.05. Exact parameters used are detailed in the respective Results sections.

### Reporting summary

Further information on research design is available in the Nature Portfolio Reporting Summary linked to this article.

## Supplementary information

Supplementary Information

Description of Additional Supplementary Files

Supplementary Data 1-15

Reporting Summary

Transparent Peer Review file

## Data Availability

Data, software, and high-resolution vector figures from the main text used in this study are described within the Article, Supplementary Information, Supplementary Data, and External Supplementary Datasets available on Figshare^41^. Specifically, the complete DArTseq dataset is provided as External Supplementary Dataset 1^41^. The DArTseq project identifiers associated with this dataset are DPoa23-8026, DPoa23-7953, DPoa23-7951, and DPoa21-6177; these identifiers can be used to integrate future samples with the current dataset.

## Code availability

All custom computer code (R and Python scripts) used to process and analyse the datasets in this study are publicly available in the Figshare repository as External Supplementary Datasets 1–4^41^. Information regarding software versions and specific parameters used for the analyses is detailed within the respective scripts and the Methods section.
