# Supplementary material for: Genomic and palaeoclimatic data reveal Pleistocene adaptation and diversification of non-model bluegrasses (Poa sect. Stenopoa) in cold, arid environments
Source: Commun Biol. 2026 Jun 5;9:1020. doi: 10.1038/s42003-026-10395-6 (PMC13408850; doi:10.1038/s42003-026-10395-6)
Supplement: Supplementary file 1 — Supplementary Information [file 42003_2026_10395_MOESM1_ESM.pdf]

Genomic and palaeoclimatic data reveal Pleistocene adaptation and diversification of non-model bluegrasses (*Poa* sect. *Stenopoa*) in cold, arid environments

Evgenii Baiakhmetov[1*], Sirilak Radbouchoom[2], Ekaterina Noskova[3,4], Kyle W. Tomlinson[1], Roman S. Romanets[5], Marina V. Olonova[5*]

[1]Center for Integrative Conservation & Yunnan Key Laboratory for Conservation of Tropical Rainforests and Asian Elephants, Xishuangbanna Tropical Botanical Garden, Menglun, 666303, China

[2]Project of Institute Establishment for Sireeruckhachati Nature Learning Park, Mahidol University, Nakhon Pathom, 73170, Thailand

[3]ETH Zurich, Institute of Integrative Biology, Zurich, 8092, Switzerland

[4]University of Edinburgh, Institute of Ecology and Evolution, Edinburgh, EH9 3FL, United Kingdom

[5]Tomsk State University, Biological Institute, Tomsk, 634050, Russia

*Corresponding authors:

Evgenii Baiakhmetov

Email: evgenii.baiakhmetov@xtbg.ac.cn

Marina V. Olonova

Email: olonova@list.ru

Contents

Supplementary Figure 1: Identification of pure sub-clusters within each of the three Clusters 3

Supplementary Figure 2: Notched boxplots of all studied morphological variables 4

Supplementary Figure 3: Evolutionary models inferred by GADMA2 5

Supplementary Figure 4: Marine isotope stages (MISs) over the past 800,000 years 6

Supplementary Figure 5: Notched boxplots of key SDM climatic variables among clusters 7

Supplementary Figure 6: Response curves for the main climatic variables used in SDM 8

Supplementary Figure 7: Frequency distributions of all studied morphological variables 9

References 10

Supplementary Figure 1: Identification of pure sub-clusters within each of the three Clusters.

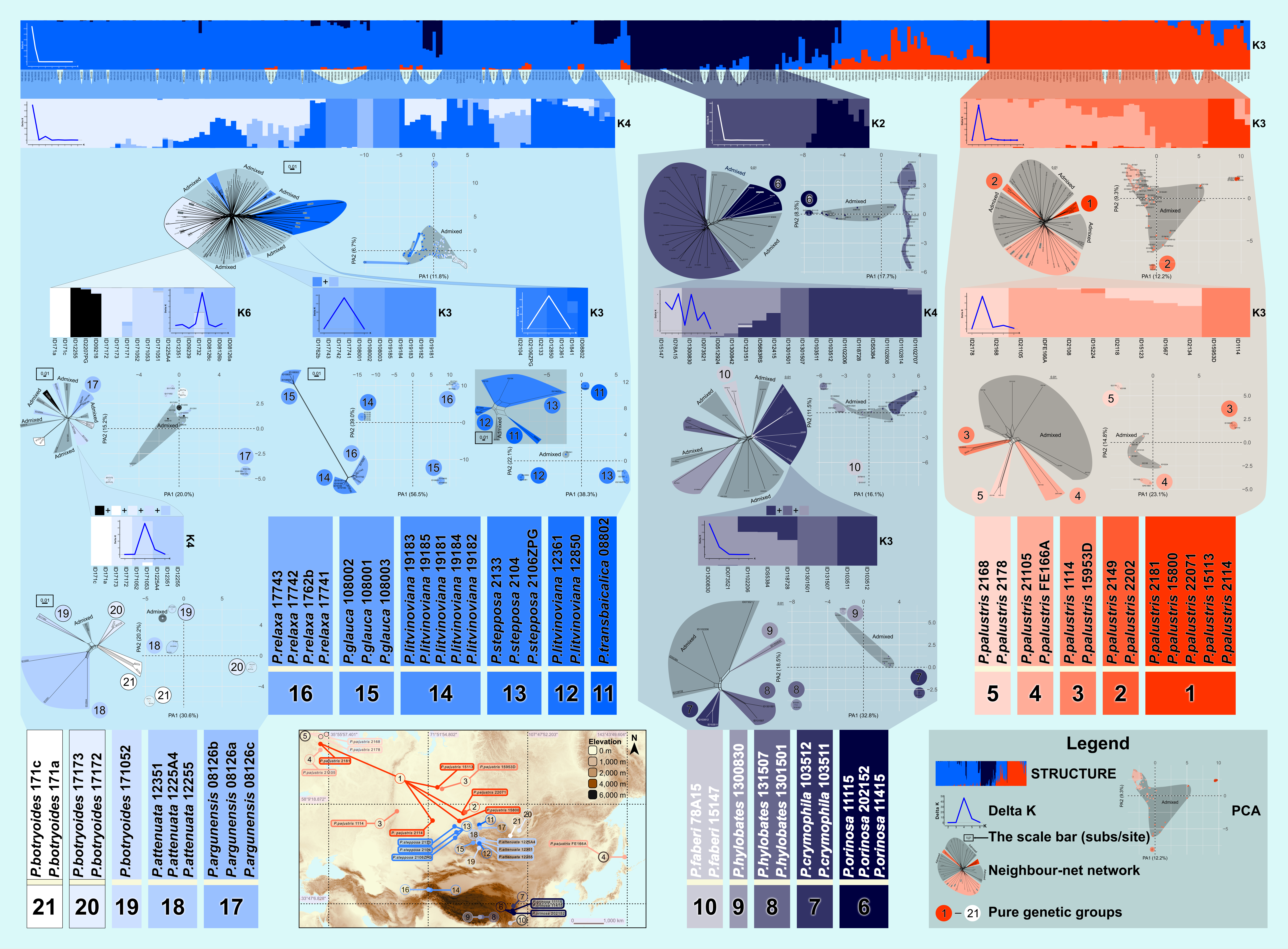

Identification of genetically pure individuals (Q ≥ 0.99) using STRUCTURE, Neighbour-Net, and PCA analyses. These analyses were used to identify pure individuals within the three initial genetic Clusters defined in Fig. 2. This step was necessary due to observed hybridisation and cytotype diversity, allowing for subsequent analyses of morphological differentiation and phylogeny inference using SNAPP (Fig. 3) by minimising violations of model assumptions (e.g., gene flow mimicking incomplete lineage sorting). Individual IDs correspond to Supplementary Data 1.

Supplementary Figure 2: Notched boxplots of all studied morphological variables.

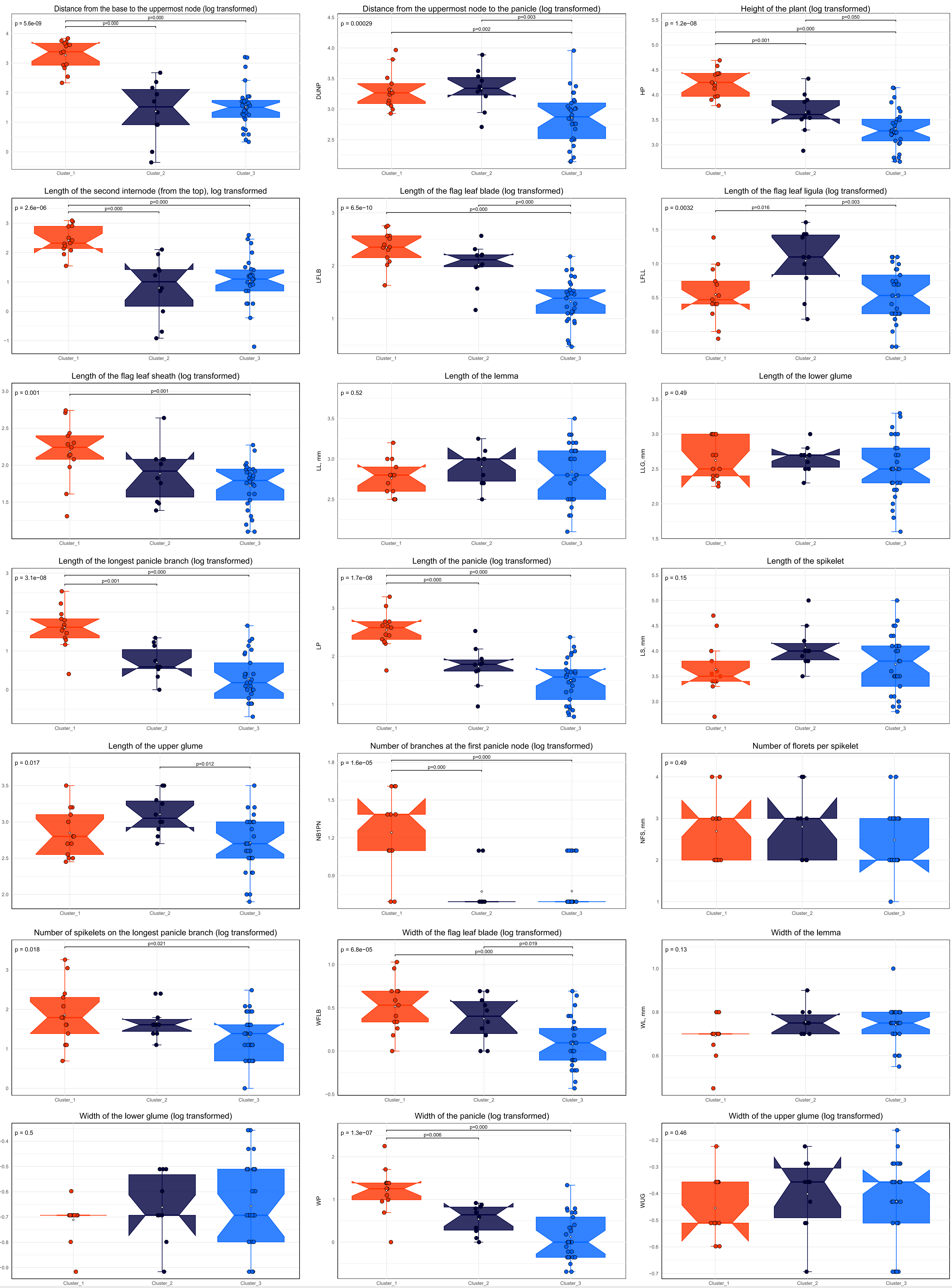

Notched boxplots demonstrating the mean (white circle), the median (thick line), the 95% confidence interval (CI) around the median (notch), the interquartile ranges (25% to 75%), the whiskers (5% and 95%), and all observations (dots). The notches display a confidence interval around the median, which is normally based on the median ± 1.57 × interquartile range / square root of n. If the notches of two boxes do not overlap, there is strong evidence (95% confidence) that their medians differ. In cases where the values of the CI are less than the lower quartile or greater than the upper quartile, the notches will extend beyond the box, giving it a distinctive 'flipped' appearance, which suggests a small sample size or high variability in the data. Statistically significant differences are denoted by numbers above solid black lines.

Supplementary Figure 3: Evolutionary models inferred by GADMA2.

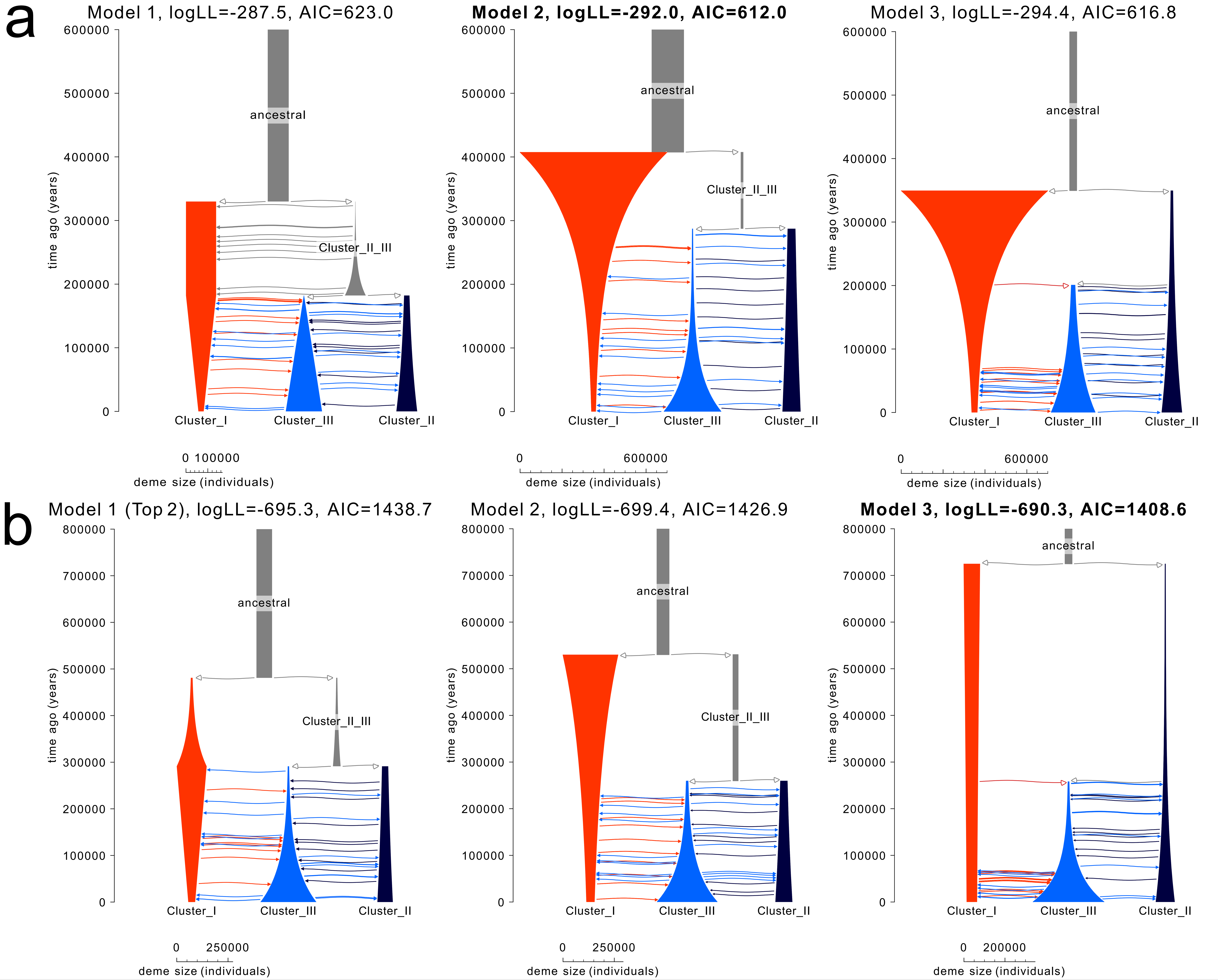

a Models inferred for SNPs with a call rate of 95%. **b** Models inferred for SNPs with a call rate of 70%. The best-supported models based on AIC scores are shown in bold. Model 1: Epoch-based with automatic population size dynamics; Model 2: Includes secondary contact between Cluster III and Cluster I (Cluster I post-split population size capped at 700,000 for illustration); Model 3: Includes an admixture event (Cluster I post-split population size capped at 700,000 for illustration).

Supplementary Figure 4: Marine isotope stages (MISs) over the past 800,000 years.

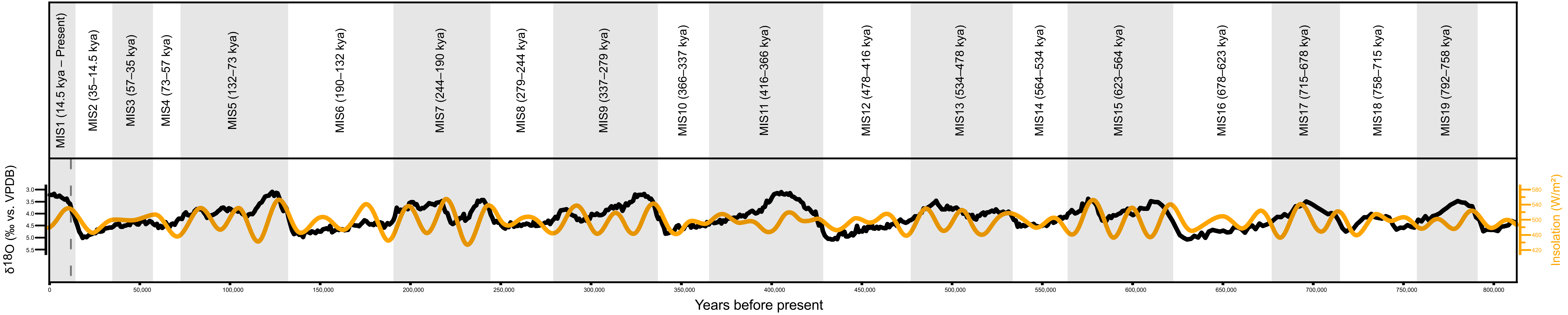

Cycles of warming (odd-numbered stages, e.g., MIS 1, 3, 5) and cooling (even-numbered stages, e.g., MIS 2, 4, 6) are shown according to Railsback et al. (2015). The black graph line depicts δ18O values (per mille) versus the Vienna Peedee Belemnite (VPDB) isotope (‰ vs. VPDB) from Lisiecki et al. (2005); the orange line shows insolation peaks associated with the interglacial periods according to Hu et al. (2024); the dashed line specifies the Pleistocene–Holocene transition (ca. 11.7 kya).

Supplementary Figure 5: Notched boxplots of key SDM climatic variables among clusters.

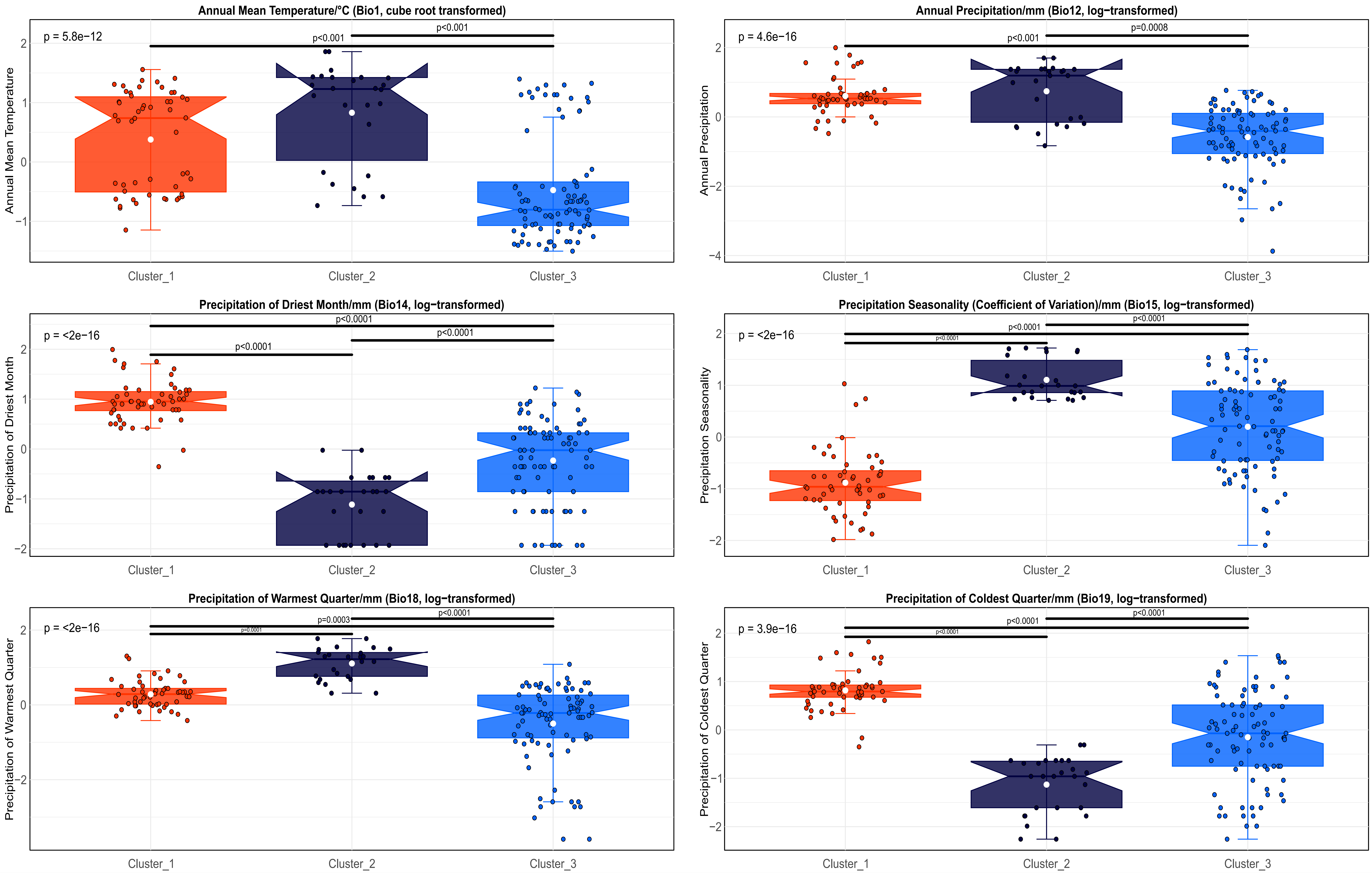

Notched boxplots demonstrating the mean (white circle), the median (thick line), the 95% confidence interval (CI) around the median (notch), the interquartile ranges (25% to 75%), the whiskers (5% and 95%), and all observations (dots). The notches display a confidence interval around the median, which is normally based on the median ± 1.57 × interquartile range / square root of n. If the notches of two boxes do not overlap, there is strong evidence (95% confidence) that their medians differ. In cases where the values of the CI are less than the lower quartile or greater than the upper quartile, the notches will extend beyond the box, giving it a distinctive 'flipped' appearance, which suggests a small sample size or high variability in the data. Statistically significant differences (derived from the analysis of pairwise distances corrected for spatial autocorrelation; External Supplementary Dataset 4) are denoted by numerical values above solid black lines.

Supplementary Figure 6: Response curves for the main climatic variables used in SDM.

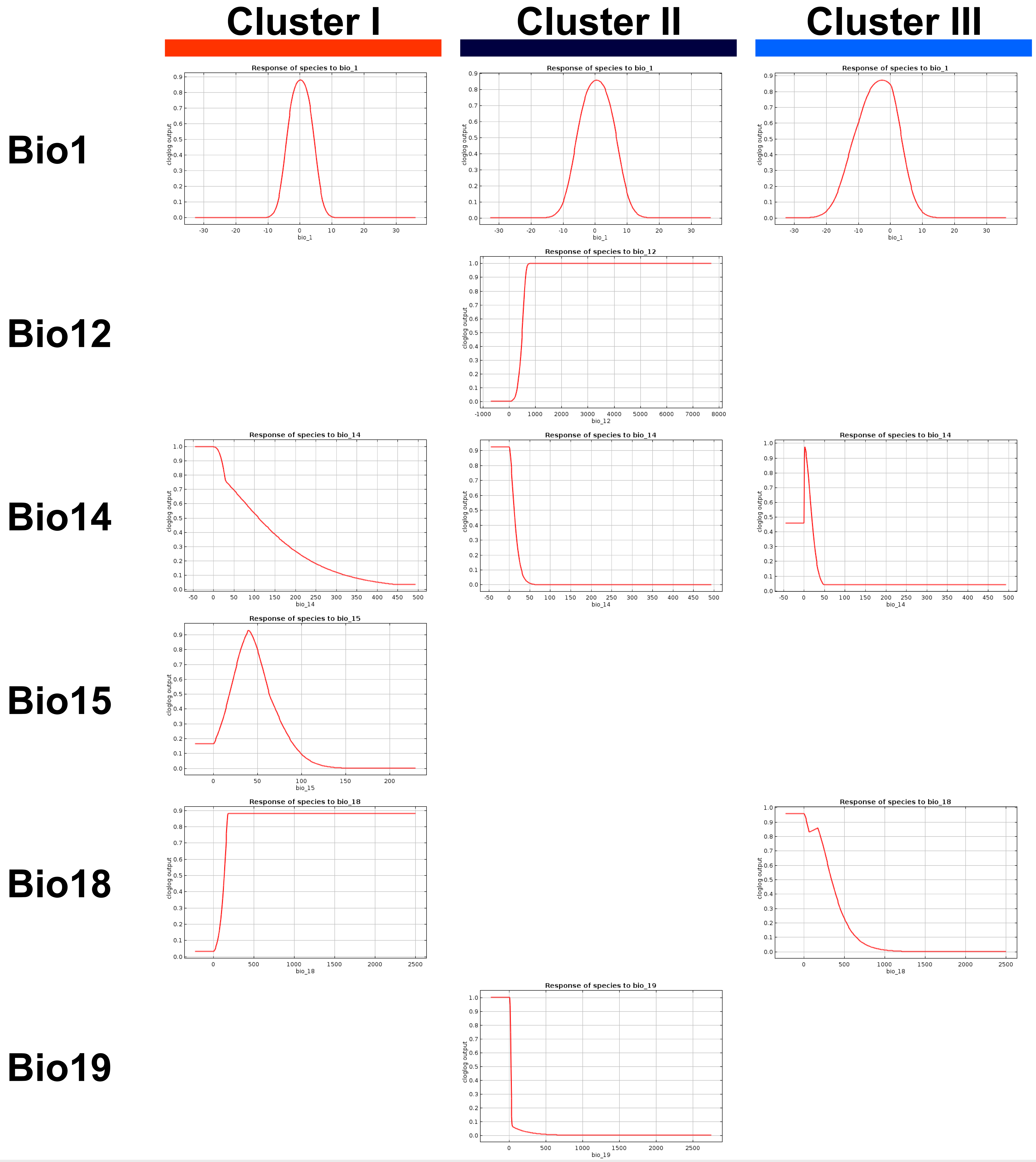

The curves show how the main environmental variables (see Supplementary Data 11) affect the MaxEnt prediction. Note that these curves can be hard to interpret when variables are strongly correlated, as the model may depend on correlations in ways not evident in the individual curves. Specifically, the curves show the marginal effect of changing only one variable, while the model may utilise interactions between variables changing together. Bio1: Annual Mean Temperature (°C); Bio12: Annual Precipitation (mm); Bio14: Precipitation of Driest Month (mm); Bio15: Precipitation Seasonality (Coefficient of Variation, mm); Bio18: Precipitation of Warmest Quarter (mm); Bio19: Precipitation of Coldest Quarter (mm).

Supplementary Figure 7: Frequency distributions of all studied morphological variables.

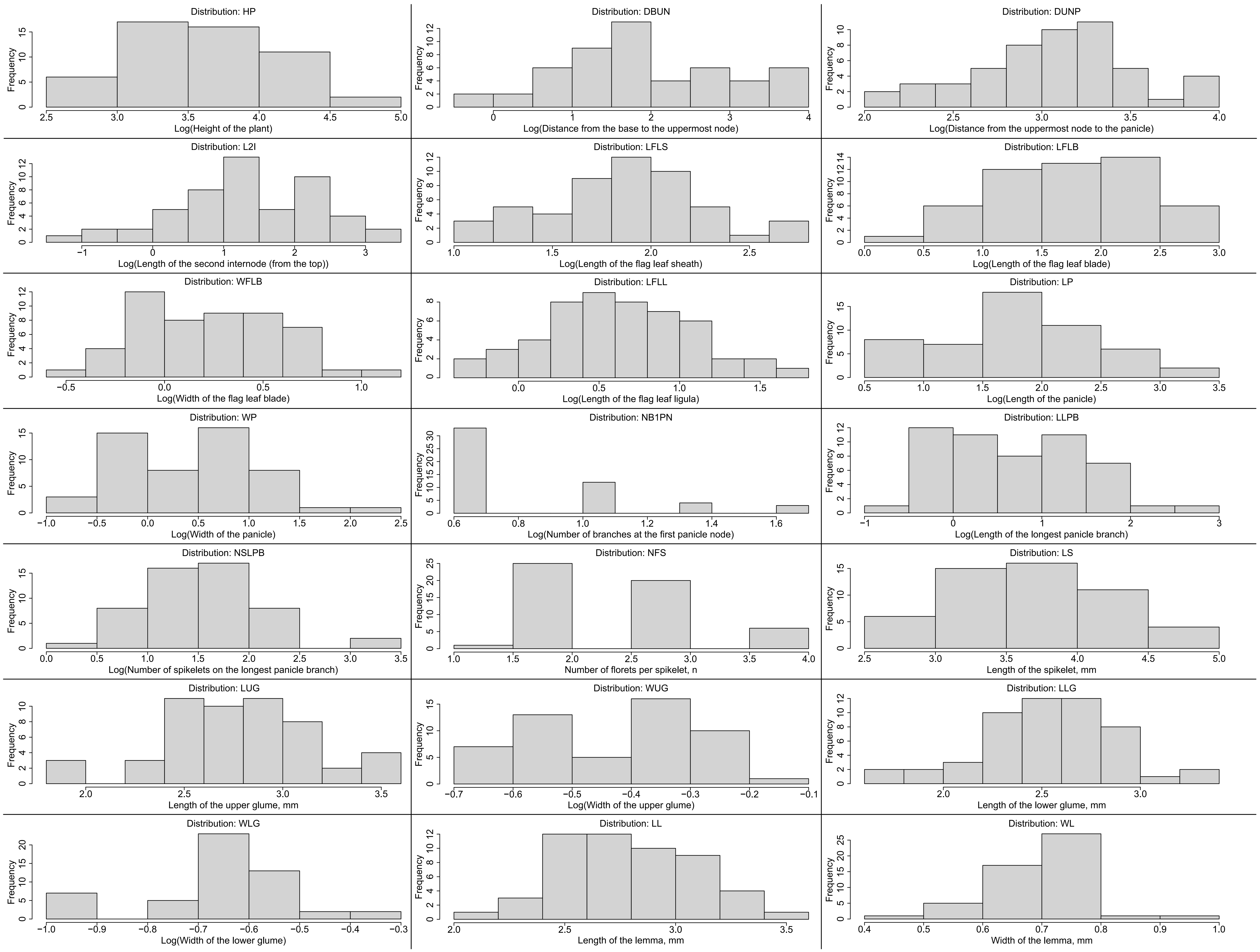

HP, DBUN, DUNP, L2I, LFLS, LFLB, WFLB, LFLL, LP, WP, NB1PN, LLPB, NSLPB, WUG, and WLG were log-transformed. Abbreviations are defined in Supplementary Data 5.

References

1. Railsback, L. B., Gibbard, P. L., Head, M. J., Voarintsoa, N. R. G. & Toucanne, S. An optimized scheme of lettered marine isotope substages for the last 1.0 million years, and the climatostratigraphic nature of isotope stages and substages. Quaternary Science Reviews 111, 94–106 (2015).
https://doi.org/10.1016/j.quascirev.2015.01.012
2. Lisiecki, L. E. & Raymo, M. E. A Pliocene-Pleistocene stack of 57 globally distributed benthic δ18O records. Paleoceanography 20, PA1003 (2005).
https://doi.org/10.1029/2004PA001071
3. Hu, H. M. et al. Sustained North Atlantic warming drove anomalously intense MIS 11c interglacial. Nature Communications 15, 5933 (2024).
https://doi.org/10.1038/s41467-024-50207-1
