# Supplementary material for: Genomic and palaeoclimatic data reveal Pleistocene adaptation and diversification of non-model bluegrasses (Poa sect. Stenopoa) in cold, arid environments
Source: Commun Biol. 2026 Jun 5;9:1020. doi: 10.1038/s42003-026-10395-6 (PMC13408850; doi:10.1038/s42003-026-10395-6)
Supplement: Supplementary file 2 — Description of Additional Supplementary Files [file 42003_2026_10395_MOESM2_ESM.docx]

**Description of Additional Supplementary Files**

**File name**: Supplementary Data 1

**Description**: List of study samples with cluster assignments.

**File name**: Supplementary Data 2

**Description**: Summary of genomic datasets, filtering parameters, and sample sizes used for population genetic analyses.

**File name**: Supplementary Data 3

**Description**: Species assignments from morphological and molecular data.

**File name**: Supplementary Data 4

**Description**: Genetic metrics for the pure Clusters.

**File name**: Supplementary Data 5

**Description**: Herbarium IDs corresponding to Figure 2 samples.

**File name**: Supplementary Data 6

**Description**: Morphological characters used in the study.

**File name**: Supplementary Data 7

**Description**: Traits contribution (%) to PCA dimensions.

**File name**: Supplementary Data 8

**Description**: Resampling performance of Recursive Feature Elimination (RFE) for variable selection.

**File name**: Supplementary Data 9

**Description**: Linear Discriminant Analysis (LDA) loadings for the RFE-selected model.

**File name**: Supplementary Data 10

**Description**: Demographic parameters for inferred cluster histories.

**File name**: Supplementary Data 11

**Description**: Palaeodistribution analysis dataset.

**File name**: Supplementary Data 12

**Description**: Bioclimatic variables used in the palaeodistribution analysis.

**File name**: Supplementary Data 13

**Description**: Species distribution model performance scores.

**File name**: Supplementary Data 14

**Description**: Percent contribution and permutation importance for the SDM.

**File name**: Supplementary Data 15

**Description**: Chromosome numbers in *Stenopoa* from literature survey.

**File name**: External Supplementary Dataset 1 | https://doi.org/10.6084/m9.figshare.29434373

**Description**: Molecular analyses: DArTseq dataset, R scripts, input and output files for SNAPP, STRUCTURE, Stairway2, and SplitsTree.

**File name**: External Supplementary Dataset 2 | https://doi.org/10.6084/m9.figshare.29434373

**Description**: Morphological dataset: input dataset, R scripts, PCA, LDA, and boxplots.

**File name**: External Supplementary Dataset 3 | https://doi.org/10.6084/m9.figshare.29434373

**Description**: GADMA analysis: input datasets, Python scripts, and output files.

**File name**: External Supplementary Dataset 4 | https://doi.org/10.6084/m9.figshare.29434373

**Description**: Species distribution modelling: SDM dataset, R scripts, spatial autocorrelation, and boxplots.
