# Supplementary material for: Genomic and palaeoclimatic data reveal Pleistocene adaptation and diversification of non-model bluegrasses (Poa sect. Stenopoa) in cold, arid environments
Source: Commun Biol. 2026 Jun 5;9:1020. doi: 10.1038/s42003-026-10395-6 (PMC13408850; doi:10.1038/s42003-026-10395-6)
Supplement: Supplementary file 5 — Transparent Peer Review file [file 42003_2026_10395_MOESM5_ESM.pdf]

nature portfolio

Peer Review File

Genomic and palaeoclimatic data reveal Pleistocene adaptation and diversification of non-model bluegrasses (Poa sect. Stenopoa) in cold, arid environments

Corresponding Author: Dr Evgenii Baiakhmetov

This file contains all reviewer reports in order by version, followed by all author rebuttals in order by version.

A version of this paper was originally rejected for publication by Communications Biology, however that decision was reconsidered after appeal by the authors.

Version 1:

Reviewer comments:

Reviewer #1

(Remarks to the Author)
I am reviewing a manuscript entitled: “Pleistocene adaptation and diversification in cold, arid environments: genomic and paleoclimate insights from non-model bluegrasses" by Dr Baiakhmetov and colleagues. The manuscript is a nice, comprehensive work describing the current distribution and within-clade genetic and morphological differentiation of the Stenopoa clade of Poa in the light of its reconstructed past areas.
While the work provides a solid foundation for future studies, some statements are overly bold, given the limited support presented in the work. For example, any discussion on adaptive mechanisms is very, very brief and insufficient, given the attention drawn to it. Similarly, polyploidy is not acknowledged, presented, analysed or discussed to the extent that could justify the emphasis on it in the abstract. In addition, the processes shaping the clade's paleoclimatic evolution are not discussed enough to provide any understanding of the effect of climate change on plant evolution.
While the study is too descriptive to fulfil the expectations from the abstract and far from demonstrating the statements in the conclusion, it has the potential to deliver a bigger picture than is presented. While the polyploidy and adaptation related parts of the manuscript should be revised in a way to provide at least some information (or in a way of removing the emphasis on them), the genetic, environmental and morphological part can be presented in a wider context of regional flora and possibly also fauna evolution during the studied glacial cycles, thus providing a valuable insight into past processes shaping observed variation. It can also be better reflected on the climate change impacts – past and future.
The manuscript Reference format is confusing – in the text, references are provided by the author's name and year; however, in the Reference section, articles are not ordered alphabetically but by numbers and appearance. Looking for a particular reference is thus not very comfortable.
Line 45: Please connect polyploidy to WGD and explain how polyploidy can be facilitated by hybridisation. Polyploidy is often viewed as an effective hybridisation barrier.
Line 47: Please explain what genetic rearrangements are meant by “These”
Line 60: The sentence suggests that the reticulate histories and blurred species boundaries are a result of the two metabolic types, C3 and C4. Please clarify.
Fig. 1: While nicely done, I found the presentation of the morphotype height misleading, given that both meso-xeromorphic and mostly xeromorphic types range is 5 to 60 cm. It is also difficult to read the numbers on the elevation slopes.
Line 124: How many populations? Was population-based sampling conducted? Please explain why not. How did the authors handle populations in the STRUCTURE program?
Line 161: No species were mentioned before. How many are there in the section, and how many were worked with? See Line 163, which is a bit uncomfortable. How was the determination made when it was not an aim of the study, and the authors claim that more work needs to be done? From the Supplementary material, which is largely species-based, it seems that there is no need for additional study.
Line 167: There were 373 individuals in Line 124. It is unclear how that became 52 in line 164 and is used for n in line 167. Please clarify. Note that the manuscript has the M&M section at the back, so the text should reflect that.
Line 173: It is not clear whether the groups 18-21 belong to Cluster I. Same for groups 12 and 14. Maybe switching the lines 174-179 with details about geographic distribution, with lines 170-173 containing some taxonomic details, would help.

Fig. 2: The figure is nice and illustrative, but so condensed that the text is very difficult to read. The bootstrap, Population codes and PCA pie charts became readable in 6x Zoom, the x-axis in UPGMA in 12x Zoom, and the tip numbers and map pie charts in 3x Zoom, which makes them all hard to find and redundant. I wonder if there is a way to enlarge the overall figure by putting, e.g. the PCA with the Fst inset on the side. It is not scaled with the map anyway. In addition, I wonder if it is possible to mark the admixed samples in the Neighbor Net pane in some shade of violet/pink/purple, similarly to the PCA plot.
Line 267: While the conclusion about the two most powerful discrimination traits is tempting, the wording suggests that the success of the adaptation does not depend on fitness but on trait size. In contrast to this, plants with short panicles may be well adapted to their environments if they have greater survival and fitness, and it may not depend on panicle length. Please clarify.
Line 272: Despite the taxonomic revision not being an aim of the study, it would be worth noting how many species are in the clade and how many species were successfully or unsuccessfully assigned. In addition, while gene flow is acknowledged between the clades, the possibility of some species being of a hybrid origin should also be mentioned. IN addition, Supplementary Table S9 shows a species name for every sample, not only 80 samples as the text suggests. Please clarify and explain how the species determination was carried out. It would be helpful to keep both, Herbarium but al so manuscript ID so that the individuals can be found out without having to consult another Supplementary Table.
Fig. 4: Boxplots for 15 characters are too small to be informative. Do the points in the plots represent the genetically pure individuals? It is unlikely to be characters as the caption for 4a reads. I suggest splitting the Figure into three parts, having the character section together with the illustration as a separate pane (c).
Fig. 5: Font size too small to be comfortable to read.
Line 405: I feel like the sentence starting with "Shared habitats…" does not link well with the previous and next sentences. Please clarify.
Line 435: The mention of polyploidy is completely out of place here. Polyploidy was not included in any of the analyses or presented separately. In the whole manuscript, polyploidy was only mentioned in the Introduction and in a very general way. Thus, I strongly suggest either incorporating polyploidy into the study or rewording any bold statements about the role of polyploidy in the evolution of the study clade. Are there any ploidy data the authors could use? Were the genetic data analysed with respect to different cytotypes? Is there any cytogeography pattern or presence in polyploids in the clades? While the Supplementary Table X provides an overview of the chromosome counts, the Table also clearly shows many aneuploid counts. Given that the chromosome counts are more or less equally distributed among the Clusters, why do authors think polyploidy plays any role in the clade evolution? While allopolyploidy is acknowledged, what about autopolyploidy and polyploid formation in harsher environments? This clade seems to be an ideal candidate for the study of the ratio of unreduced gamete formation in stressful environments.
Line 436: Any note about the economic significance of this lineage is missing in the Introduction, so it is surprising to find it in the Discussion. It feels out of place and context there. Please rewrite.
Line 444: Are there any particular adaptations to aridity? While morphological results were shown, no adaptive traits were mentioned or later discussed. This is a similar case to the polyploidy: stating that the plant is polyploid and adapted without showing how is too strong a statement for a discussion, where it implies it is a result of the presented manuscript. In contrast, studying adaptation or polyploidy was not included in the study, and no details about adaptive mechanisms or ploidy levels are mentioned in the Results, Materials section or Introduction (for the adaptive mechanisms). Please rewrite with respect to the fact that some information about adaptation is provided two paragraphs later (line 463), and polyploidy is briefly discussed in line 483. Regarding polyploidy again, any discussion about unreduced gametes formation under harsh conditions would nicely fit line 514 and further. It should also be mentioned that there are studies comparing the presence of diploids and polyploids in formerly glaciated areas, which may help your discussion about the clade evolution during the glacial cycles.
Line 566: This sentence feels very redundant, given that the work did not acknowledge the existence of polyploids in any of the analyses.
Line 575: Please explain how your findings can suggest anything about the role of polyploidy when polyploidy was not part of the study, and no ploidy patterns were presented. The only mention of polyploidy is that there are overlapping chromosome counts presented in all clusters, which does not show or support any role of polyploidy.
Line 578: The last sentence of the Discussion is in contrast with the previous findings' summary throughout the manuscript. While it may not be untrue, it is a very dense shortcut that should be elaborated and reflect on the actual presented results, e.g. from line 464 (morphological traits driving phenotypic divergence) and line 480 (morphological evolution as a direct adaptive trait to distinct ecological niches and climatic pressures therein). If polyploidy and hybridisation play any role in providing genetic redundancy and buffer against environmental conditions (paraphrasing line 515 here), it should be an aim of future research, together with detecting the genomic basis of Stenopa (likely morphological) adaptation. It would also be worth elaborating on how exactly this study contributes to the broader understanding of climate change effects on plant evolution (line 579).
Line 585: How many populations were used? Is each population represented by one individual?
Line 590: The double digest restriction-site associated DNA genotyping term usually refers to ddRADseq (Peterson et al. 2012), not DArT (Killian et al. 2012). While both methods use restriction enzymes to reduce the DNA representation, the library preparation and data format are very different. The authors should clearly explain the differences between DArT and ddRADseq and why they use the term coined for another method. In addition, since the Killian et al. 2012 protocol is no longer in use, the authors should provide a relevant reference. Note that the Methods description should be self-standing and reproducible. Using a misleading term for the methods together with an outdated reference obviously is not.
Line 605: Was there any size selection? Why or why not? Why were so many PCR cycles, possibly introducing sequencing errors, used? What was the length of the sequenced fragments?
Line 600: What is a DArT PL script? A reference or an explanation why it is not provided is needed. A newer reference for DArT than Killian et al. 2012 should be provided, given the differences between the initial DArT and the method the authors (and the company) used.

Line 612: Even if proprietary, the authors still should refer to the software, server, company, etc.
Line 622: A reference for the DArTsoft14 should be provided.
Line 625: "Using a range of parameters" is a very vague statement. Provide details.
Line 631: Provide the version and a reference for R.
Line 648: Explain the low number of STRUCTURE iterations for burn-in (10,000) and MCMC chains (100,000). It is common to have a burn-in of 100,000 and MCMC of 1,000,000 iterations. I am reluctant to accept conclusions relying on what is a burn-in phase in most other studies.
Line 672: I am not sure "structure" is the right word.
Line 674: What was the ratio of individuals per cluster? Were the clusters equally represented?
Line 713: DArT protocol sequence length should also be mentioned in the sequencing section of the Methods (see my related comment).
Line 724-733: This paragraph should follow after the first mention of the two datasets in the first paragraph of this section, keeping the information about the datasets together.
Line 734: How many populations were used and how many individuals were sampled per population? If you mean clusters by populations (as in line 738), explain it in Line 734, at the first mention of the populations in the manuscript, to avoid confusion and enhance clarity.
Line 738: What is the difference between the first two models from the three models mentioned in this line and the two models referred to in Line 734?
Line 751: Does this mean that the genetic dataset contains duplicates and erroneous entries?
Line 754: If only 143 individuals were used for the modelling, how many of the remaining (373-143 =) 230 individuals were removed due to spatial thinning and how many were removed during the cleaning? How were the genetically admixed samples handled if the modelling was cluster-based and there were only 52 genetically pure individuals that could be unambiguously assigned to each cluster?
Line 759: Keep shortcuts consistent with the main text; use either Kya or ka throughout the text.
Line 760, 762: Refer to the databases consistently, including the references.
Supplementary Table S2: The table lists the same analyses with a different number of individuals without providing any details, which makes it rather useless. For example, there are STRUCTURE (Cluster III) analyses with either 113, 18, 12, 9 or 7 individuals. What are we supposed to do with this?
Supplementary Table S4: This table has an unfortunate format. If kept separately, condense it into multiple rows. However, if possible, incorporate the information about ID and Herbarium number into the relevant Supplementary Tables S9 and S11.
Supplementary Table S5 and S6: Please condense into one table.
Supplementary Table S8: Please add full character name – plenty of space there.
Supplementary Table S11: What is Raster ID?
Supplementary Table S15: The format is unfortunate. CCDB is a secondary source with references to the primary source. The primary source always has to be checked due to the way the database was (automatically) assembled. Thus, this column should be incorporated in the reference column. The column before the references misses a headline. Are those chromosome numbers coming from different sources than the ones used in CCDB? What does the section Other represent if the Stenopoa clade is represented by three clusters revealed in this study? Please clarify. While polyploidy refers to whole-genome duplication, chromosome numbers outside the multiplication of the basic chromosome number (7) indicate other processes that should also be discussed in the text. Given the variation in chromosome numbers equally distributed among the clusters, what would be the role of polyploidy (assuming taxa with aneuploid chromosome numbers are polyploids of some kind)? Is polyploidy only a result of a hybridisation (allopolyploids origin from the secondary contact), or is there another possible explanation, such as higher unreduced formation in harsh environments leading to autopolyploids and aneuploids?
Supplementary Figure S7: These are likely not histograms, but a distribution. Clarify the headline.

Reviewer #2

(Remarks to the Author)
Review
Pleistocene adaptation and diversification in cold, arid environments: genomic and
palaeoclimate insights from non-model bluegrasses
This is an interesting analysis of Poa sect. Stenopoa complexes, and fairly novel at that.
The paper seems to be very well written and intelligible. Aside from too many acronyms to keep track of says this blurry eyed reviewer.
The English is clear and natural.
Ligula should be corrected to Ligule
The analytical methods appear to be sound. Cluster analyses and PCA are widely familiar.
This reviewer is not versed in the other statistical methods of analyses employed.
The manuscript covers a lot of ground with other statistics but seems appropriate in length for all of that.
The figures are well done and appropriate.
The discussion of the geography of climate zones, growing season lengths and timing, and ppt patterns and amounts and corresponding morphology is useful. This reviewer doesn't understand how this relates to population sizes (bottlenecks?).
The analysis of gross morphological vegetative measures used in PCA Dimensions is interesting also. And well done. This

reviewer noticed the absence of pubescence characters of lemmas and calluses (often important for taxon separation in Poa), and absence of characters of prickle hairs “p/a”, and their direction (antrorse versus retrorse) below stem nodes which Tzvelev (2009) in particular, considered of high importance for taxon discrimination, and found to be quite useful by the present reviewer. A comment on why these traits were ignored would be appreciated. Basically, because they are qualitative, not quantitative. This study could not have been done without Olonova’s extensive and careful scoring of morphological variations on individual specimens and identifications.
It is interesting that the authors were able to identify a few speciation gaps and peaks corresponding to peak glacial periods (spanning up to seven interglacial periods).
The DNA extraction method would seem to favor Chloroplast DNA over nuclear DNA, since plastid DNA is in many thousands of copies compared to nuclear DNA, beyond nuclear ribosomal repeats. PSTI And HpaII are infrequent restriction site cutters of chloroplast DNA. Was there an attempt to distinguish plastid from nuclear DNA or single copy nuclear DNA from repeat nuclear ribosomal DNA? If they could factor for this, I wonder they could estimate the data contribution from plastid versus nDNA or nrDNA contributions to the cluster analyses, or if chloroplast DNA could be screened for, in or out, in the subsequent analyses for SNPs. Plastid DNA inheritance in Angiosperms is rarely from pollen parents. Were they able to detect ploidy levels in samples?
Some brief background on Poa sect. Stenopoa diversity in the introduction would help readers grasp the scope of the problem of Stenopoa. (Range of estimates of the number of Stenopoa species would also be useful. Brief discussion of species concepts?) Tzvelev & Probatova 2019 included 51 species for Russia! Liu 2003 ca 120 species for China.
Cluster I is only Poa palustris?. More discussion of the selection / identification of “genetically pure individuals” and exclusion of other samples or taxa would be helpful. e.g. What happened to Poa nemoralis? Poa nemoralis is another mesomorphic species that seems to share the identical TLF plastid sequences in multiple samples (5 each), differing from most other Stenopoas a bit, while their nrDNA profiles are diverse and do not unite these two taxa. (24 Stenopoa taxa sampled for plastid sequences by Gillespie and Soreng pers. comm here: more than 30 Stenopoa taxa have been sequenced for ITS nrDNA: many samples contributed by or co-collected by Olonova. You are welcome to cite this pers. comm. if you like and feel it is appropriate).
The other two clusters, II & III, correspond to sets of species.
To this reviewer, the separation of Poa palustris (cluster I) from the other taxa seems to present a rather circular argument that the mesomorphic form corresponds to the mesomorphic habitat.
The vouchering is well done in Suppl. Table 1. Are all the sample vouchers at TK? The distribution of the samples/taxa in clusters I, II, III or overlapping/admixture is made clear in Suppl. Table I. This could also be done in Suppl. Table 9, where mismatches between DNA and morphology are noted among all samples. Some relevant taxonomic implications for Stenopoa taxa would be of interest.
Poa x figertii (compressa x nemoralis) and Poa x intricata (nemoralis x palustris) present in cluster I were published as hybrids. Some comments on their Pure versus Admixture characterizations, and DNA mismatches would be interesting. These could be marked as hybrid formulae in Suppl. Table 1.
After all this work it seems unfortunate to this reviewer that we don’t have publicly deposited sequence data that can be applied to future studies. Can this study be built upon with more samples. Or would it need a whole redo?
Sequence data and analyses for Poa, including section Stenopoa species are available from quite a few more recent papers than cited in the text. This reviewer suggests expanding the references in this regard. Stenopoa taxa DNA sequences tend to show limited variation. This is also true of some other Poa clades including the very speciose (and the largest) clade of Poa in supersection Homalopoa in nrDNA and plastid analyses.

Cabi, E., R. J. Soreng & L. J. Gillespie. 2017. Taxonomy of Poa jubata and a new section of the genus (Poaceae). Turk. J. Bot. 41: 404–415, f. 1–4.
Cabi, E., R. J. Soreng, L. J. Gillespie & N. Amiri. 2016. Poa densa (Poaceae), an overlooked Turkish steppe grass, and the evolution of bulbs in Poa. Willdenowia 46(2): 201–211.
Gillespie, L. J., R. J. Soreng & S. W. L. Jacobs. 2009. Phylogenetic relationships of Australian Poa (Poaceae: Poinae), including molecular evidence for two new genera, Saxipoa and Sylvipoa. Austral. Syst. Bot. 22(6): 413–436, f. 1–3.
Gillespie, L. J., R. J. Soreng, E. Cabi & N. Amiri. 2018. Phylogeny and taxonomic synopsis of Poa subgenus Pseudopoa (including Eremopoa and Lindbergella) (Poaceae, Poeae, Poinae). PhytoKeys 111: 69–101.
Giussani, L. M., L. J. Gillespie, M. A. Scataglini, M. A. Negritto, A. M. Anton & R. J. Soreng. 2016. Breeding system diversification and evolution in American Poa supersect. Homalopoa (Poaceae: Poeae: Poinae). Ann. Bot. (Oxford) 118(2): 281–303.
Refulio-Rodríguez, N. F., J. T. Columbus, L. J. Gillespie, P. M. Peterson & R. J. Soreng. 2012. Molecular phylogeny of Dissanthelium (Poaceae: Pooideae) and its taxonomic implications. Syst. Bot. 37(1): 122–133.
Soreng, R. J. & L. J. Gillespie. 2018. Poa secunda J. Presl (Poaceae): a modern summary of infraspecific taxonomy, chromosome numbers, related species and infrageneric placement based on DNA. PhytoKeys 110: 101–121.
Soreng, R. J., L. J. Gillespie & L. L. Consaul. 2017. Taxonomy of the Poa laxa group, including two new taxa from Arctic Canada and Greenland, and Oregon, and a re-examination of P. sect. Oreinos (Poaceae). Nordic J. Bot. 35: 513–538.
Soreng, R. J., L. J. Gillespie, E. Boudko & E. Cabi. 2022. Biogeography, timing, and life-history traits in the PPAM clade: Coleanthinae (syn. Puccinelliinae), Poinae, Alopecurinae superclade, Miliinae, and Avenulinae and Phleinae (Poaceae, Pooideae, Poeae). J. Syst. Evol. 60(3): 591-620.
Soreng, R. J., L. J. Gillespie, H. Koba, E. Boudko & R. D. Bull. 2015. Molecular and morphological evidence for a new grass genus, Dupontiopsis (Poaceae tribe Poeae subtribe Poinae s.l.), endemic to alpine Japan, and implications for the reticulate origin of Dupontia and Arctophila within Poinae s.l. J. Syst. Evol. 53(2): 138–162.
Soreng, R. J., M. V. Olonova, N. S. Probatova & L. J. Gillespie. 2020. Breeding systems and phylogeny in Poa L., with special attention to northeast Asia: the problem of Poa shumushuensis Ohwi and sect. Nivicolae (Poaceae). J. Syst. Evol. 58(6): 1031–1058.
Soreng, R. J., R. D. Bull & L. J. Gillespie. 2010. Phylogeny and reticulation in Poa based on plastid trnTLF and nrITS

sequences with attention to diploids. 619–644. In O. Seberg, G. Petersen, A. S. Barfod & J. I. Davis (eds.) Diversity, Phylogeny, Evol. Monocot. Aarhus University Press, Aarhus.
Sylvester, S. P., R. J. Soreng & L. J. Gillespie. 2021. Resolving páramo Poa (Poaceae): morphometric and phylogenetic analysis of the 'Cucullata complex' of north-west South America. Bot. J. Linn. Soc. 197: 104-146.

Also include:
For dating of grasses.
Gallaher, T. J., P. M. Peterson, R. J. Soreng, F. O. Zuloaga, D.-Z. Li, L. G. Clark, C. D. Tyrrell, C. A. D. Welker, E. A. Kellogg & J. K. Teisher. 2022. Grasses through space and time: An overview of the biogeographical and macroevolutionary history of Poaceae. J. Syst. Evol. 60(3): 522-569.
And for Stenopoa diversity relating to the controversy over species delimitations.
In Russia
Tzvelev, N. N. & N. S. Probatova. 2019. Zlaki Rossii / Grasses of Russia. In N. N. Tzvelev & N. S. Probatova Zlaki Rossii / Grasses of Russia. KMK Scientific Press, Moscow.
Tzvelev, N. N. 2009. On species of the genus Poa L. section Stenopoa Dumort. (Poaceae) in the eastern Europe. Novosti Sist. Vyssh. Rast. 41: 18–52.
Olonova, M. V. 1990 35 Poa L. Fl. Sibir. 2: 165–189
Olonova, M. V. 1998 The system and check-list of Siberian bluegrasses (Poa L.) Turczaninowia 1(4): 5–19
Olonova, M. V. 2007 Variability of bluegrass (Poa) in Siberia Aliso 23: 435–449
Olonova, M. V. 2008 Towards the study of bluegrass complex Poa versicolor Bess. (Poaceae) in northern China Animadvers. Syst. Herb. Univ. Tomsk. 99: 16–24
Olonova, M. V. 2010 The synopsis of section Stenopoa of Poa (Poaceae) in Siberia Bot. Zhurn. (Moscow & Leningrad) 95(6): 869–881
Olonova, M. V. 2016 Synopsis of blugrasses (Poa L., Poaceae) of the Altai mountain system Sist. Zametki Mater. Gerb. Krylova Tomsk. Gosud. Univ. Kuybysheva 113: 68–88
Olonova, M. V. & E. G. Nikolin 2018 Bluegrasses (Poa L., Poaceae) of Sakha (Yakutia) Republic, an annotated checklist Adansonia, sér. 3, 40(1): 89–102
Olonova, M. V. & S. P. Sylvester 2021 Poa liuliangii and P. kengii (Poaceae), two new species from the Hengduan Mountains of southwest China Taiwania 66(2): 251-257
Olonova, M. V. & V. V. Chepinoga 2022 Poa probatovae (Poaceae), a new species from the Eastern Siberia Turczaninowia 25(1): 140-145
Olonova, M. V. & W.-L. Chen 2010 Systematic revision of the Poa sphondylodes Trin. complex (Poaceae) Candollea 65(1): 135–141
Olonova, M. V., M.E. Barkworth, S. V. Pulkina & W.-L. Chen 2011 Morphological variation in populations of the Poa orinosa complex (Poaceae: Poeae) in northeastern China Nordic J. Bot. 29: 762–772
Olonova, M. V., V. D. Shiposha & R. S. Romanets 2022 Phenotypic plasticity of the stem epidermis in the bluegrasses (Poa L.) of section Stenopoa Dumort. (Poaceae). I. Mesomorphic species Acta Biol. Sibirica 8: 655-672
The Himalaya front ranges
Rajbhandari, K. R. 1991. A revision of the genus Poa L. (Gramineae) in the Himalaya. Bull. Univ. Mus. Univ. Tokyo 34: 169-263, f. 1-29 + maps f. 30-43.
In China
Liu, L. 2003. Poa. Fl. Reipubl. Popularis Sin. 9(2): 91–226.
Liu 2003 listed 246 species of Poa from China, as this reviewer recalls about 120 were attributable to Stenopoa species. Whereas Olonova (in Zhu et al. 2006) only accepted 15 species but many infraspecies. (below -- ! = ok name when homonyms exist, * = illegitimate names, ** = invalid names.)
Poaceae ! Poa botryoides (Trin. ex Griseb.) Kom.
Poaceae Poa chaixii Vill.
Poaceae Poa compressa L.
Poaceae Poa khasiana Stapf
Poaceae Poa malacantha Kom.
Poaceae Poa sichotensis Prob.
Poaceae Poa stepposa (Krylov) Roshev.
Poaceae Poa urssulensis Trin.
Poaceae Poa almasovii Golub
Poaceae Poa altaica Trin.
Poaceae ! Poa arctica R. Br.
Poaceae ! Poa attenuata Trin.
Poaceae Poa badensis Haenke ex Willd.
Poaceae Poa bulbosa subsp. vivipara (Koeler) Arcang.
Poaceae Poa densa Troitsky
Poaceae ! Poa hybrida Gaudin
Poaceae Poa insignis Litv.
Poaceae Poa paucispicula Scribn. & Merr.
Poaceae Poa platyantha Kom.
Poaceae ! Poa pumila Host
Poaceae Poa shumushuensis Ohwi
Poaceae ! Poa sibirica Roshev.
Poaceae ! Poa sylvicola Guss.
Poaceae Poa tolmatchewii Roshev.

Poaceae Poa transbaicalica Roshev.
Poaceae Poa vrangelica Tzvelev
Poaceae Poa ampla Merr.
Poaceae Poa annua var. reptans Hausskn.
Poaceae Poa eminens J. Presl
Poaceae Poa irkutica Roshev.
Poaceae Poa smirnowii Roshev.
Poaceae ! Poa acroleuca Steud.
Poaceae Poa hisauchii Honda
Poaceae Poa nipponica Koidz.
Poaceae Poa radula Franch. & Sav.
Poaceae Poa hayachinensis Koidz.
Poaceae Poa sachalinensis (Koidz.) Honda
Poaceae Poa sphondylodes Trin.
Poaceae Poa viridula Palib.
Poaceae Poa lanata Scribn. & Merr.
Poaceae ! Poa laxa Haenke
Poaceae Poa tibeticola Bor
Poaceae ! Poa remota Forselles
Poaceae ! Poa albertii Regel
Poaceae ! Poa angustiglumis Roshev.
Poaceae Poa bracteosa Kom.
Poaceae Poa bucharica Roshev.
Poaceae Poa bulbosa subsp. nevskii (Roshev. ex Ovcz.) Tzvelev
Poaceae Poa ciliatiflora Roshev.
Poaceae Poa dahurica Trin.
Poaceae Poa densissima Roshev. ex Ovcz.
Poaceae Poa dschungarica Roshev.
Poaceae ! Poa hissarica Roshev. ex Ovcz.
Poaceae * Poa irrigata Lindm.
Poaceae ! Poa karateginensis Roshev. ex Ovcz.
Poaceae Poa kolymensis Tzvelev
Poaceae Poa komarovii Roshev.
Poaceae Poa korshunensis Golosk.
Poaceae Poa krylovii Reverd.
Poaceae ! Poa laudanensis Roshev. ex Ovcz.
Poaceae Poa lipskyi Roshev.
Poaceae Poa lipskyi subsp. dschungarica (Roshev.) Tzvelev
Poaceae ! Poa longifolia Trin.
Poaceae Poa meyeri Trin. ex Roshev.
Poaceae ! Poa nevskii Roshev. ex Ovcz.
Poaceae Poa ochotensis Trin.
Poaceae ! Poa pamirica Roshev. ex Ovcz.
Poaceae Poa phariana Bor
Poaceae ! Poa pruinosa Korotky
Poaceae Poa raduliformis Prob.
Poaceae Poa rangkulensis Ovcz. & Czukav.
Poaceae Poa relaxa Ovcz.
Poaceae ! Poa rhomboidea Roshev.
Poaceae Poa sabulosa (Turcz. ex Roshev.) Turcz. ex Roshev.
Poaceae ! Poa schischkinii Tzvelev
Poaceae Poa sibirica subsp. uralensis Tzvelev
Poaceae Poa subfastigiata Trin.
Poaceae Poa tianschanica (Regel) Hack. ex O. Fedtsch.
Poaceae Poa tristis Trin. ex Regel
Poaceae Poa trivialiformis Kom.
Poaceae Poa versicolor Besser
Poaceae Poa versicolor subsp. relaxa (Ovcz.) Tzvelev
Poaceae Poa versicolor subsp. stepposa (Krylov) Tzvelev
Poaceae Poa vvedenskyi Drobow
Poaceae Poa zaprjagajevii Ovcz.
Poaceae ! Poa trichophylla Heldr. & Sartori ex Boiss.
Poaceae ! Poa ianthina Keng ex Shan Chen
Poaceae ! Poa pachyantha Keng ex S.L. Chen
Poaceae ! Poa patens Keng ex Keng f.
Poaceae ! Poa paucifolia Keng ex Shan Chen
Poaceae ! Poa perennis Keng ex Keng f.
Poaceae ** Poa plurinodis Keng ex Keng f.
Poaceae Poa mairei Hack.

Poaceae Poa arnoldii Melderis
Poaceae Poa bomiensis C. Ling
Poaceae Poa borealitibetica C. Ling
Poaceae ! Poa crymophila Keng ex C. Ling
Poaceae * Poa digena Melderis
Poaceae Poa langtangensis Melderis
Poaceae ! Poa macrolepis Keng ex C. Ling
Poaceae ! Poa malaca Keng
Poaceae ! Poa micrandra Keng
Poaceae Poa nigropurpurea C. Ling
Poaceae Poa nimuana C. Ling
Poaceae ! Poa oligophylla Keng
Poaceae ! Poa orinosa Keng
Poaceae ! Poa tunicata Keng ex C. Ling
Poaceae ! Poa vaginans Keng
Poaceae Poa zhongbaensis C. Ling
Poaceae ! Poa megalothyrsa Keng ex Tzvelev
Poaceae Poa roemeri Bor
Poaceae Poa lhasaensis Bor
Poaceae Poa afghanica Bor
Poaceae Poa alta Hitchc.
Poaceae Poa arjinsanensis D.F. Cui
Poaceae Poa florida N.R. Cui
Poaceae Poa macroanthera D.F. Cui
Poaceae Poa major D.F. Cui
Poaceae Poa membranigluma D.F. Cui
Poaceae Poa nemoralis var. parca N.R. Cui
Poaceae Poa parvissima Cui ex D. F. Cui
Poaceae ! Poa poophagorum Bor
Poaceae * Poa scabristemmed N.R. Cui
Poaceae Poa kelungensis Ohwi
Poaceae Poa imperialis Bor
Poaceae ** Poa mongolica (Rendle) Keng
Poaceae Poa asperifolia Bor
Poaceae Poa himalayana Nees
Poaceae Poa polyneuron Bor
Poaceae Poa spontanea Bor
Poaceae Poa burmanica Bor
Poaceae Poa eleanorae Bor
Poaceae Poa gamblei Bor
Poaceae Poa jaunsarensis Bor
Poaceae Poa kanboensis Ohwi
Poaceae Poa nephelophila Bor
Poaceae Poa nitidespiculata Bor
Poaceae Poa rhadina Bor
Poaceae Poa sinoglauca Ohwi
Poaceae Poa wardiana Bor
Poaceae Poa grandis Hand.-Mazz.
Poaceae Poa rossbergiana K.S. Hao
Poaceae Poa taiwanicola Ohwi
Poaceae Poa debilior Hitchc.
Poaceae ! Poa argunensis Roshev.
Poaceae ! Poa dshilgensis Roshev.
Poaceae Poa formosae Ohwi
Poaceae Poa nankoensis Ohwi
Poaceae ! Poa reverdattoi Roshev.
Poaceae Poa takasagomontana Ohwi
Poaceae Poa tenuicula Ohwi
Poaceae Poa diversifolia (Boiss. & Balansa) Hack. ex Boiss.
Poaceae Poa hirtiglumis Hook. f.
Poaceae Poa prolixior Rendle
Poaceae Poa shansiensis Hitchc.
Poaceae Poa tangii Hitchc.
Poaceae Poa faberi Rendle
Poaceae Poa pilipes Keng ex Shan Chen
Poaceae Poa granitica Braun-Blanq.
Poaceae Poa acmocalyx Keng ex L. Liu
Poaceae Poa binodis Keng ex L. Liu
Poaceae ! Poa breviligula Keng ex L. Liu

Poaceae Poa chalarantha Keng ex L. Liu
Poaceae ! Poa declinata Keng ex L. Liu
Poaceae * Poa elanata Keng ex L. Liu
Poaceae Poa eragrostioides L. Liu
Poaceae Poa fascinata Keng ex L. Liu
Poaceae Poa flavida Keng ex L. Liu
Poaceae Poa gracilior Keng ex L. Liu
Poaceae Poa grandispica Keng ex L. Liu
Poaceae ! Poa incerta Keng ex L. Liu
Poaceae Poa hengshanica Keng ex L. Liu
Poaceae Poa lepta Keng ex L. Liu
Poaceae ** Poa levipes (Keng) Keng ex L. Liu
Poaceae Poa lithophila Keng ex L. Liu
Poaceae Poa maerkangica L. Liu
Poaceae ! Poa nubigena Keng ex L. Liu
Poaceae Poa pubicalyx Keng ex L. Liu
Poaceae Poa schoenites Keng ex L. Liu
Poaceae Poa tetrantha Keng ex L. Liu
Poaceae Poa stereophylla Keng ex L. Liu
Poaceae Poa varia Keng ex L. Liu
Poaceae Poa yakiangensis L. Liu
Poaceae Poa zhongdianensis L. Liu
Poaceae Poa pratensis var. anceps (Gaudin) Griseb.
Poaceae Poa platyglumis (L. Liu) L. Liu
Poaceae Poa nemoralis subsp. nemoralis
Poaceae Poa bulbosa var. bulbosa
Poaceae * Poa media Schur
Poaceae Poa annua var. annua
Poaceae Poa pseudamoena Bor
Poaceae ! Poa alpigena Lindm.
Poaceae Poa skvortzovii Prob.
Poaceae ** Poa pseudopalustris Keng
Poaceae Poa ludens R.R. Stewart
Poaceae Poa szechuensis Rendle
Poaceae Poa ligulata Boiss.
Poaceae ** Poa spiciformis D.F. Cui
Poaceae ** Poa dolichachyra Keng ex L. Liu
Poaceae * Poa indattenuata Keng ex L. Liu
Poaceae * Poa pseudopalustris Keng ex L. Liu
Poaceae * Poa psilolepis Keng ex L. Liu
Poaceae * Poa sinattenuata Keng ex L. Liu
Poaceae ** Poa stenachyra Keng ex L. Liu
Poaceae Poa parafestuca L. Liu
Poaceae Poa timoleontis Heldr. ex Boiss.
Poaceae Poa masenderana Freyn & Sint.
Poaceae ! Poa pratensis subsp. alpigena (Lindm.) Hiitonen
Poaceae ! Poa abbreviata R. Br.
Poaceae Poa sikkimensis (Stapf) Bor
Poaceae Poa supina Schrad.
Poaceae Poa stewartiana Bor
Poaceae Poa bactriana subsp. glabriflora (Roshev.) Tzvelev
Poaceae Poa sinaica Steud.
Poaceae ! Poa glabriflora (Roshev.) Roshev. ex Ovcz.
Poaceae Poa koelzii Bor
Poaceae ! Poa nepalensis (Wall. ex Griseb.) Duthie
Poaceae ! Poa lahulensis Bor
Poaceae ! Poa pagophila Bor
Poaceae Poa setulosa Bor
Poaceae Poa stapfiana Bor
Poaceae ! Poa litvinoviana Ovcz.
Poaceae ! Poa alpina L.
Poaceae ! Poa angustifolia L.
Poaceae ! Poa nemoralis L.
Poaceae Poa araratica Trautv.
Poaceae Poa palustris L.
Poaceae Poa sterilis M. Bieb.
Poaceae Poa infirma Kunth
Poaceae Poa falconeri Hook. f.
Poaceae Poa aitchisonii Boiss.

Poaceae Poa bactriana Roshev.
Poaceae ! Poa calliopsis Litv. ex Ovcz.
Poaceae Poa tibetica Munro ex Stapf
Poaceae Poa polycolea Stapf
Poaceae Poa arctica subsp. caespitans Simmons ex Nannf.
Poaceae Poa macrocalyx Trautv. & C.A. Mey.
Poaceae ! Poa glauca Vahl
Poaceae !! Poa pratensis L.
Poaceae ! Poa annua L.
Poaceae Poa bulbosa var. vivipara Koeler
Poaceae ! Poa bulbosa L.
Poaceae Poa trivialis L.
Poaceae Poa triglumis Keng f. ex L. Liu
Poaceae ! Poa plurifolia Keng
Poaceae Poa longiglumis Keng ex L. Liu

Reviewer #3

(Remarks to the Author)
The present manuscript is an ambitious and integrative study that combines different approaches from genomics to morphology and paleoclimate niche construction analysis to understand diversification dynamics in one of interesting yet taxonomically very complex plant lineages characterized by whole genome duplication. I am impressed by how the authors rigorously conducted the study and incorporated sound analytical approaches to investigate the evolutionary dynamics of a non-model polyploid taxa and improve our understanding of Pleistocene diversification in grass. However, I have some critical methodological issues that, if addressed, may improve the current version of the manuscript:
• Line 483: As already mentioned in the paper, polyploidy is the central focus of the study system and the key driver of diversification and adaptation. However, it is surprising not to see even a single attempt to do a direct chromosome count or ploidy estimation in the present paper. Instead, the authors decided to rely on previously published and incomplete chromosome number data and state that chromosome counting is beyond the scope of the study. Given that the present paper is framed around polyploidy, this statement is completely unacceptable and potentially makes any of the biological interpretations made in the study questionable as one cannot be sure if any of the inferred groups reflect true evolutionary lineage or are influenced by cytotype variation.
• Line649 & Line126: In the method section (line 649) the authors describe how the optimal K was determined objectively; the way the results are presented (line 126) appears more subjective as the authors claim that the data “can be divided into two or three “clusters. This way of presentation is not acceptable. The authors should clearly describe how they objectively determined the optimal K by reporting and describing the result from the Evannon deltaK method and whether the chains converged across the different runs, including an assessment of cluster stability across replicated runs.
• The use of reduced-representation snp data (DarTseq) is well suited for inferring genetic structuring and recent divergence but to robust testing of fine-scale demographic inference may be challenging, particularly in plant lineages characterized by extensive polyploidy, hybridization, and reticulation. Furthermore, some of the analytical methods assume diploid and unlinked loci. I would therefore expect the authors to justify, in the method section, the suitability of these methods for polyploidy taxa or alternatively to tone down the interpretation of the results and discuss them with some curiosity.

Version 2:

Reviewer comments:

Reviewer #1

(Remarks to the Author)
I am reviewing the manuscript entitled “Pleistocene adaptation and diversification in cold, arid environments: genomic and paleoclimate insights from non-model bluegrasses" by Dr Baiakhmetov and colleagues. This is my second time reviewing (Reviewer #1 in the previous revision).

First of all, I appreciate the work the authors did, especially with presenting polyploidy and cytological variation as a background evolutionary context and not a tested variable. I also like that the authors provided more details about morphological and environmental traits and presented them in a wider context. The added paragraph about taxonomic complexity now provides more context for their discussion while not dwelling on too many taxonomic details and complexity, which seems to be one of the next steps in understanding Stenopoa evolution.

Despite the great improvement of the already very nice manuscript, the study remains mostly Poa-oriented and descriptive. And, if the authors conclude that their study “provides a predictive framework for understanding how ongoing climate change might
shape the future distribution and evolution of cold-adapted polyploid flora”, I would expect them to show such a prediction in their own work.

I still have several comments which the authors may find useful in helping the manuscript clarity and coherence, and some

additional questions about the work and its results.

Line 45: Please rephrase the sentence to accommodate allopolyploidy not being the only mechanism of polyploid formation. I suggest something along the lines “Among the mechanisms of polyploid formation, WGD may follow after hybridisation (allopolyploidy).”
In addition, polyploidy is not driven by hybridisation; it is a mechanism that sometimes follows hybridisation and enables plants to deal with two distinct chromosome sets. Not all hybrids are allopolyploids. Importantly, the fertility restoration due to polyploidy does not always happen (e.g. not all allopolyploids are fertile), so the statement should be softened (e.g., by adding may). Plus, a reference example for fertility restoration should be provided.
This first paragraph still uses WGD, genome doubling and polyploidy without really linking the terms together. I suggest adding polyploidy and genome doubling into the brackets in line 42: (WGD, aka polyploidy or genome doubling). While this is not absolutely accurate, it provides at least some connection between these terms.

Lines 94 and 97: What species concepts are referred to here?
While the biological species concept is widely recognised, it is also widely acknowledged as unsuitable in most plant species because of the presence of, e.g. polyploidy or apomixis. Are there any other species concepts that would be more suitable for the study group? Would a new species concept help to solve the taxonomic complexity, as was done, e.g. in the case of Rubus? I do not dispute the fact that the group is likely a taxonomical nightmare, but I am inclined that it has a lot to do with the morphological variation, phenotypic plasticity and the lack of determining characters (plus polyploidy, apomixis, etc.), rather than the biological species concept only.

Line 100: Apomixis is generally considered a breeding system (a shift from sexual to asexual reproduction). Consider rephrasing, including apomixis, together with decliny as another example of a shift in breeding system.

Line 147: The formulation about selecting the number of clusters is still not precise. The data can be (theoretically) divided into many clusters, which is why additional methods were developed to avoid subjectivity and help to determine the best number of clusters. Consider rewriting, e.g. “According to the clustering methods (UPGMA; unweighted pair … and STRUCTURE; Bayesian clustering method), the dataset can be optimally divided into two clusters (K=2, …). However, we also show results for /or we proceeded with K=3 (K=3, …), due to …. And the clusters are referred to as …”.
Regarding the determination of the correct deltaK for the K=2, was a K=1 runs from STRUCTURE used for the Evanno method? While K=1 gives the same result, it is needed to correctly estimate the deltaK for K=2. Why were only K=1 to K=15 runs run? How did the clusters separate in higher Ks? Were the pure sub-clusters detected in higher Ks? Why not?

Line 185: What is a morphospecies, and why is it used in this context? I am not sure how (and why) the authors got from morphospecies to divisions supported by a multispecies coalescent model.

Line 196: I appreciate the authors' effort to rewrite these paragraphs following my previous comments. However, from the present text, I am not sure how to deal with two descriptions of the cluster geographic distribution - one general (line 155) and one derived from the sub-clusters. Please consider additional rewriting with highlighting the potential differences or similarities between the distributions.

Line 202: I am generally confused by this paragraph. What taxa does “these” refer to? What is a “stable genetic group”? Do the authors mean sites by populations in line 204 (population in this context implies mixed-species populations/sites, which remains unknown given the sampling)?

Line 211: Please consider the wording here; STRUCTURE analysis does not identify the models or numbers of clusters; it shows the level of genetic admixture depending on the K number of clusters.

Line 221: What is a pure genetic cluster? Do you mean an individual/individuals without admixture merged together? What about “between the genetically pure individuals from each cluster”?

Line 225: Please consider the same rewording here to avoid confusion from the overusage of the term cluster.

Figure 3: Why are only some species names shown on the map, and what were the selection criteria? The sub-clusters 6, 14 or 18 also have multiple accessions; all sub-clusters have 2 or more individuals, while there is only one dot shown on the map - is that due to the geographical proximity and potential graphical overlap?

Line 230: Please elaborate on BBP.

Line 235: Please clarify the sentence suggesting you used SNAPP to establish genetic groups.

Line 248: Is phenotypic plasticity really a result of past gene flow? Phenotypic plasticity enables plants of the same genotype to quickly adapt to various or changing environments, without relying on the mutation/selection processes or a suitable inherited genetic combination. Consider, if you are not referring to morphological variation instead. Although these two are often difficult to tell apart without experimental cultivation, I think that the latter term would be more suited for a situation involving gene flow.

Line 264: I cannot open the interactive images here, although the links at the end of the manuscript worked. It does not mean anything other than my poor computer skills. I believe the link was tested by several of the authors. I am mainly pointing it our

because in this case, I am not able to see why the reference to the original Figure 4 is missing and whether the figure is different from the same link in line 240 (the link there did not work for me either but as long as other people can open it, I appreciate the effort of the authors to make the Figures more easy to handle, at least in the digital version).

Line 272: Please clarify what the authors mean by pre-defined genetic clusters. While discriminant analyses work with pre-defined groups, are you still referring to the same 52 genetically pure individuals that were used for the PCA analysis? I am also not sure whether the wording is accurate in this paragraph. The authors are basically testing the ability of selected (pre-selected key) traits to distinguish the genetic clusters, not identifying the best characters by testing all available traits. I do not dispute the method, but I assume the authors are testing whether the selected traits are enough for clade differentiation. I think it should be clarified. Are the selected key traits common traits used in determination keys or taxonomic literature? Or are those traits the ones with the highest contribution to PCA axes?

Line 302: I am not sure phenotypic is the right word here, since the analyses employed are primarily intended to test morphological variation, not phenotypic. Even the selection of only genetically pure individuals does not mean all individuals from the same cluster are genetically uniform, and the tested phenotype would be stable/variable under different climatic conditions.

Line 303: I miss a context for the reassigned 80 individuals. Do the authors mean that integration of genetic data and identified/verified morphological traits enabled the further determination of an additional 80 individuals? Where were the 80 individuals reassigned? To the genetic clusters or to some species that were well differentiated within the clusters? Or did the authors try to determine the genetic cluster based on the morphological characters only and then double-check its genetic assignment?
Supplementary Table 9 shows no P. transbaicalica in the “Species name assigned by morphology” column. However, all samples assigned to P. stepposa were shown to belong to P. transbaicalica. How is that possible? How do the authors know after having genetic data that the previous assignment shall differ? What is the morphological difference between these two species, and why was it not spotted before obtaining the genetic data? Very similar is the situation for P. attenuata - how were the individuals with a known genetic background assigned to a species that was not determined in the “morphology column”? I understand if a single individual with a certain morphology falls into a uniform genetic group representing different species (also recognised in the morphology column. What I do not understand is how a group of individuals assigned by morphology to P. attenuata becomes P. transbaicalica in the genetic column without P. transbaicalica being recognised by morphology at all.

Line 324: Please provide references for GADMA2 and Starway Plot 2.

Line 388: Please consider replacing kya with Kya to make it consistent with the remaining text. Same for the dating in the top pane of the Figure.

Line 395: Please provide references for the MaxEnt modelling, AUC, and TSS.

Line 409: What are the unique adaptations of Cluster II to the monsoonal climate? If they are morphological, did the traits contribute to the clusters’ differentiation in morphological analyses?

Line 425: Please consider preplacing shrank by shranks to align the verb with the singular
subject, Cluster II.

Line 444: Should the word “Cluster” precede “the initial II-III ancestral line” in this sentence?

Line 482: I suggest replacing the word lineage " with " clade/section, if applicable. Using lineage implies that the authors focused on a polyploid lineage within the Stenopoa section, which I believe is not the case.

Line 487: I am wondering if mentioning the monsoonal preference/occurrence for Cluster II would also be of interest in this paragraph. I am also wondering if species is a proper word to use, given that several taxa were found polyphyletic, admixed, or there was a discrepancy between the initial and later assignment.

Line 495: Please consider calling the data morphological instead of phenotypic, mainly due to the unknown character stability in different conditions.

Line 496: Note that the selection of 3 clusters was a subjective decision of the authors, however well justified by geography. The Evanno test for the STRUCTURE analysis showed that K=2 is the best number of clusters. From this (objective/statistical) view, the genetic data confirmed the morphological ones.

Line 500: Please consider including morphological variation as well. It should not be omitted, especially if the phenotypic plasticity was not tested or documented in this group.

Line 538: I appreciate the authors’ efforts in changing the manuscript approach to polyploidy. I am wondering, why did the authors not consider autopolyploidy as another mechanism of polyploid formation? In a loose sense, autopolyploidy is not a WGD only within a population but also within a lineage/species. If there are more chromosome counts within the P. palustris lineage, the higher ones may as well arise from autopolyploidisation. Telling these two mechanisms (auto- vs. allo-polyploidy) apart is generally difficult and may be extremely hard in such a complex group, such as Stenopoa.

Line 566: Please consider rephrasing the exceptional ploidy levels by replacing them or adding the exceptional variation in chromosome numbers, which should better reflect the frequent aneuploidy and dysploidy.

Line 593: Please consider replacing "identifies" with "identified" - most of the text is in the past tense, and I do not see a reason why it should be different here.

Line 597: Since the paragraph in the Introduction nicely showed that the number of species largely depends on the taxonomic approach, can you somehow estimate the proportion these two species represent in the Stenopoa section or in your dataset? The literature listing the chromosome counts should mention the source populations used for the chromosome counting - is it possible to trace the species or the diploid populations, and if so, are the populations (or at least the species) still persisting in the regions the authors identified as the possible regions of Stenopoa origin? If so, it would be a nice confirmation of the presented hypothesis.

Line 624: Is there any study on Poa or Stenopoa actually testing for phenotypic plasticity? It was not acknowledged in the text, and as I mentioned in my previous comments, morphological variation and phenotypic plasticity are often difficult to tell apart without experimental work.

Line 640: Where in the phylogeny is the closest reliable fossil? What closest group has a time-calibrated phylogeny, and could those species be included in future projects as outgroups to obtain better calibration, e.g. with molecular clock/ mutation rate methods that do not rely on primary calibration points, i.e. fossils?

Lines 688 - 692 nicely illustrate why I asked for more details about the protocol and called the Kilian et al (2012) an outdated reference - while it may describe the principle of the method (and is used in this context in the references kindly provided by the authors in their Rebuttal letter), the protocol the company uses is very much different from the one described in the paper and these modifications should have been acknowledged. Given the stark difference in details given for the “simple step”, such as DNA extraction and the “complex step”, such as the library preparation, I suggest adding (line 689) the library preparation “done by Diversity Arrays … following their internal protocols” included …

Line 720: The SNP abbreviation should be elaborated when first mentioned in the text, not here.

Line 737: I am not sure why this is a new paragraph - method details for UPGMA are given in the previous one, while PCA and NeighbourNet, together with STRUCTURE, are given in this one.

Line 746: Please consider a new paragraph for the new methods/analyses.

Line 755: Please consider clarification of what the pure clusters mean, e.g., “pure individuals within each cluster”, and replacing “individually” with “separately”.

Line 808: Please check for possibly missing word(s) after coalescent.

Lines 819-825: Unlike other paragraphs describing the parameters used for a particular method, this one describes the method itself. Please consider unifying this paragraph with the others.

Line 834: Please replace these populations with the genetic clusters here and throughout the paragraph where appropriate (everywhere except population size dynamics in line 842).

Line 834: Does the trajectory here refer to the dynamics in the previous sentence? If so, please consider using the same term for better clarity. If not, please elaborate on trajectories in the first model.

Line 849: It seems like the authors (Grower et al) refer to it as Demes. Please do the same.

Line 856: Were the spatial duplicates from the same genetic cluster or from different ones? Similarly, were the records within the 1km2 grid cell from the same cluster? If not, I wonder if keeping both records would not yield a more accurate paleodistribution reconstruction/prediction.

Line 870: Additionally to what?

Line 875: The list of climatic variables would better fit into the result section of the manuscript.

Line 887: From the context, I assume the terms taxa and species used here actually refer to the three genetic clusters. Please clarify. If it refers to the species, are those taxa or species the species assigned to the pure sub-clusters identified in Supplementary material 1? Similarly, do the species in line 888 refer to certain Stenopoa species or to the three clusters mentioned at the beginning of this methods section? How were the species’ known ecological preferences and environmental conditions determined? From floristic literature? Can the authors provide a list of references somewhere? Do they correspond to the reconstructed ecological preferences and environmental conditions of the clusters?

Line 896: Please clarify what the Statistical significance of the cluster term means. Should it be nugget instead?

Line 908: I appreciate the work the authors did on this Table.

Reviewer #2

(Remarks to the Author)
The ms is siginifcantly improved
I wonder why this Tzvelev article is not commented on?
Tzvelev, N. N. 2009. On species of the genus Poa L. section Stenopoa Dumort. (Poaceae) in the eastern Europe. Novosti Sist. Vyssh. Rast. 41: 18–52.
It would seem to deserve a comment:

Tzvelev recognize many Stenopoa species in Eastern Europe former USSR region.
Poaceae Poa hypanica Prokudin Accepted By
Poaceae ! Poa nemoralis L. Accepted By
Poaceae Poa lapponica Prokudin Accepted By
Poaceae Poa palustris L. Accepted By
Poaceae Poa volhynensis Klokov Accepted By
Poaceae Poa urssulensis Trin. Accepted By
Poaceae ! Poa tanfiljewii Roshev. Accepted By
Poaceae Poa podolica (Błocki ex Asch. & Graebn.) Błocki ex Zapał. Accepted By
Poaceae Poa versicolor Besser Accepted By
Poaceae ! Poa polonica Błocki Accepted By
Poaceae Poa sterilis M. Bieb. Accepted By
Poaceae ! Poa biebersteinii H.N. Pojark. Accepted By
Poaceae Poa erythropoda Klokov Accepted By
Poaceae Poa transbaicalica Roshev. Accepted By
Poaceae ! Poa glauca Vahl Accepted By
Poaceae Poa bryophila Trin. Accepted By
Poaceae Poa balfourii Parn. Accepted By
Poaceae Poa janczewskii Zapał. Accepted By
Poaceae Poa compressa L. Accepted By
Poaceae ** Poa taurica H.N. Pojark. Accepted By
Poaceae Poa rehmannii (Asch. & Graebn.) Woł. Accepted By
Poaceae Poa austrouralensis Tzvelev Published In
Poaceae Poa nemoralis var. popovii Tzvelev Published In
Poaceae Poa nemoralis var. zhitomirica Tzvelev Published In
Poaceae * Poa orientalis Tzvelev Published In
Poaceae Poa kulikovii Tzvelev Published In
Poaceae Poa lapponica var. turiensis Tzvelev Published In
Poaceae Poa egorovae Tzvelev Published In
Poaceae Poa klokovii Tzvelev Published In
Poaceae Poa palustris var. halophila Tzvelev Published In
Poaceae Poa sterilis var. bodrakense Tzvelev Published In
Poaceae Poa sterilis var. juzepczukii Tzvelev Published In
Poaceae Poa paczoskii Tzvelev Published In
Poaceae Poa saksonovii Tzvelev Published In
Poaceae Poa korshinskii Tzvelev Published In
Poaceae Poa korshinskii var. birskensis Tzvelev Published In
Poaceae Poa korshinskii var. capitolinae Tzvelev Published In
Poaceae Poa igoshinae Tzvelev Published In

Reviewer #3

(Remarks to the Author)
I appreciate the efforts made by the authors to thoroughly address the issues raised, including both my comments and those of the other reviewers. I find the response satisfactory and have no further concerns.

Version 3:

Reviewer comments:

Reviewer #1

(Remarks to the Author)
This is my third review of the manuscript “Pleistocene adaptation and diversification in cold, arid environments: genomic and paleoclimate insights from non-model bluegrasses" by Dr. Baiakhmetov and colleagues (Reviewer #1 in the previous

revisions).

I greatly enjoyed reading the presented revised manuscript. The great amount of work the authors did in the first version of the manuscript is now presented in a more complex synthesis, focusing on showing a clear story about how the interplay of climatic changes, morphological variation and possible phenotypic plasticity together with polyploidy and hybridization shaped demographic history and diversification of a taxonomically complicated group. The expanded discussion section about possible evolution and distribution under future changing climate adds to the manuscript significance and further elevates the already interesting results presented in the previous versions.

The authors found a good balance between providing enough details about the complex group and the difficulties it poses without overloading the text with too many taxonomical details. Thus, while the text clearly admits the limitations and sets directions for future studies, it it a very good standalone work of great interest for anyone interested in ecological and morphological adaptation, Pleistocene flora evolution, hybridization and also their joint effect on diversification and evolution.

I appreciate the efforts the authors made to thoroughly address my comments. I still have a few suggestions the authors may consider to review, most of them being more about editing the text.

Most part of the first paragraph is polyploidy-oriented, which I believe is a residue from the first version of the manuscript which stressed polyploidy. In this version, I feel like it draws more focus on polyploidy than is actually needed and I was wondering if a paragraph focusing more on the interplay between e.g., morphology and environment or climatic changes driving the flora evolution or migrations in the study area would be more appropriate. On the other hand, the paragraph is nice and clear and except for possibly steering the expectations of the readers from the actual aims toward polyploidy, there is nothing “wrong” with the paragraph if the authors decide to keep it.

I was wondering if the result section about Genetic structure and molecular dating could after the first sentence (lines 149-150) continue with lines 189-191 or 192, that briefly present the methodological framework before jumping directly into the STRUCTURE results?

Lines 159-167 describe the geographic distribution of Clusters I and III, however, it is not immediately clear that Cluster II distribution is actually already provided in line 155. Please consider moving the details about Cluster II distribution into the following paragraph.

Line 215: Why the two species mentioned here do not have the author ’s name while the two other species mentioned in this paragraph do?

Line 717: Please consider deleting “years” after 2022.

Open Access This Peer Review File is licensed under a Creative Commons Attribution 4.0 International License, which permits use, sharing, adaptation, distribution and reproduction in any medium or format, as long as you give appropriate credit to the original author(s) and the source, provide a link to the Creative Commons license, and indicate if changes were made.

In cases where reviewers are anonymous, credit should be given to 'Anonymous Referee' and the source.
The images or other third party material in this Peer Review File are included in the article’s Creative Commons license, unless indicated otherwise in a credit line to the material. If material is not included in the article’s Creative Commons license and your intended use is not permitted by statutory regulation or exceeds the permitted use, you will need to obtain permission directly from the copyright holder.
To view a copy of this license, visit https://creativecommons.org/licenses/by/4.0/

##

Reviewers' comments:

##

Referee #1: Plant systematics and ecology

Reviewer #1 (Remarks to the Author):

##

I am reviewing a manuscript entitled: “Pleistocene adaptation and diversification in cold, arid environments: genomic and paleoclimate insights from non-model bluegrasses" by Dr Baiakhmetov and colleagues. The manuscript is a nice, comprehensive work describing the current distribution and within-clade genetic and morphological differentiation of the Stenopoa clade of Poa in the light of its reconstructed past areas.

• While the work provides a solid foundation for future studies, some statements are overly bold, given the limited support presented in the work. For example, any discussion on adaptive mechanisms is very, very brief and insufficient, given the attention drawn to it. Similarly, polyploidy is not acknowledged, presented, analysed or discussed to the extent that could justify the emphasis on it in the abstract. In addition, the processes shaping the clade's paleoclimatic evolution are not discussed enough to provide any understanding of the effect of climate change on plant evolution.

Reply: We agree that the original manuscript placed disproportionate emphasis on polyploidy as a tested mechanism without sufficient primary data presentation, and that the discussion of adaptation was too brief. We have performed a comprehensive revision to address these points:

- To ensure polyploidy is properly "acknowledged and presented", we have added a "Cytological data compilation" section to the Methods (**Lines 904–909**) and a "Cytological variation" section to the Results (**Lines 462–472**), incorporating the data from Supplementary Table 15. This provides the necessary foundation for the Discussion.
- We have revised the Abstract and the Main text to frame polyploidy as the evolutionary context rather than the primary variable being tested.
- We have expanded the Discussion to move beyond general statements. We now link our results with the broader colonisation patterns observed in plants in formerly glaciated landscapes (e.g., **Lines 569–572**). Furthermore, we highlight how climatic fluctuations (aridisation and fragmentation) acted as a universal catalyst for *Poa* diversification, mirroring the evolutionary history of lineages in the Southern Hemisphere (**Lines 579–583**).
- We discuss morphological traits as functional trade-offs (e.g., competitive ability vs. stress tolerance) to explain adaptive mechanisms in the Results (**Lines 297–300**) and Discussion (**Lines 491–492**).
- We pay more attention to the Limitations of the study, emphasising that incorporating precise cytological data (distinguishing diploids, polyploids, and aneuploids) is essential to fully disentangle the specific influence of genome duplication and dysploidy on evolutionary responses to climate change (**Lines 637–639** & **646–648**).

• While the study is too descriptive to fulfil the expectations from the abstract and far from demonstrating the statements in the conclusion, it has the potential to deliver a bigger picture than is presented. While the polyploidy and adaptation related parts of the manuscript should be revised in a way to provide at least some information (or in a way of removing the emphasis on them), the genetic, environmental and morphological part can be presented in a wider context of regional flora and possibly also fauna evolution during the studied glacial cycles, thus providing a valuable insight into past processes shaping observed variation. It can also be better reflected on the climate change impacts – past and future.

Reply: We appreciate the reviewer's encouragement to elevate the study beyond a descriptive case study. We have revised the manuscript to contextualise our findings within global evolutionary trends:

- Following the reviewer's suggestion, we now clarify that allopolyploidisation is not the sole mechanism driving diversity. We explicitly highlight aneuploidy, dysploidy, and the formation of unreduced gametes as critical drivers of the observed cytotypic complexity (the Discussion; **Lines 539–545**).
- We link our results with broader colonisation patterns observed in plants in formerly glaciated landscapes (**Lines 569–572**) and identify climatic fluctuations (specifically aridisation and fragmentation) as a universal driver of diversification across the genus, drawing parallels with lineages in the Southern Hemisphere (**Lines 579–583**).
- We have completely rewritten the Conclusion (**Lines 646–655**) to focus on the broader implications of the study. We now frame our results regarding historical habitat fragmentation and refugia as a framework for understanding how cold-adapted polyploid flora may respond to future climatic shifts.

• The manuscript Reference format is confusing – in the text, references are provided by the author's name and year; however, in the Reference section, articles are not ordered alphabetically but by numbers and appearance. Looking for a particular reference is thus not very comfortable.

Reply: We apologise for this inconsistency. The reference list has now been corrected and ordered alphabetically.

• Line 45: Please connect polyploidy to WGD and explain how polyploidy can be facilitated by hybridisation. Polyploidy is often viewed as an effective hybridisation barrier.

Reply: We have revised the Introduction (**Lines 45–46**) to explicitly link WGD to polyploidy and to specify that hybridisation drives allopolyploidy by restoring fertility in sterile hybrids.

• Line 47: Please explain what genetic rearrangements are meant by "These"

Reply: We have revised the preceding sentence (**Lines 46–47**) to explicitly mention "structural rearrangements" and "genetic redundancy" resulting from WGD and hybridisation. This ensures that the phrase "These genomic rearrangements" has a clear antecedent, referring to the structural changes and doubling events described immediately prior.

• Line 60: The sentence suggests that the reticulate histories and blurred species boundaries are a result of the two metabolic types, C3 and C4. Please clarify.

Reply: We agree that the original sentence inadvertently implied that metabolic types (C3/C4) directly cause reticulate histories. We have revised the Introduction (**Lines 61–63**) to clarify that

while ecological adaptability allows grasses to flourish, the taxonomic complexity arises specifically from the evolutionary mechanisms (hybridisation and polyploidy) that often underpin this success.

• **Fig. 1: While nicely done, I found the presentation of the morphotype height misleading, given that both meso-xeromorphic and mostly xeromorphic types range is 5 to 60 cm. It is also difficult to read the numbers on the elevation slopes.**

Reply: We appreciate the reviewer's feedback on Figure 1. We have increased the font size of the elevation numbers to ensure they are legible.

Regarding the height of the plants, the illustrations are intended to depict the representative morphotypes corresponding to the three genetic clusters identified in this specific study. As shown in our morphometric analysis (Fig. 4, HP boxplot), there is a statistically significant trend in plant height among our samples (Cluster I > Cluster II > Cluster III). Crucially, this distinctness reflects our methodological strategy. As clarified in the Methods (**Lines 753–759**), we focused on identifying cohesive, "pure" genetic groups to avoid the blurring of morphological traits caused by putative transgressive segregation in hybrids. Thus, the figure represents the data-driven trends of these core groups. However, this natural phenotypic plasticity causes significant overlap across the section's global range. We have ensured the Figure 1 caption explicitly describes these overlapping height ranges to prevent misleading interpretations (**Lines 126–128**).

• **Line 124: How many populations? Was population-based sampling conducted? Please explain why not. How did the authors handle populations in the STRUCTURE program?**

Reply: As detailed in the updated "Plant material and sampling strategy" section (**Lines 662–666**), our study prioritised maximum geographic coverage across Eurasia using historical herbarium vouchers. Consequently, sampling was not population-based in the traditional sense; most localities are represented by single individuals.

Regarding the STRUCTURE analysis, because we lacked population-level sampling for most sites, the analysis was performed without using sampling origin as a prior (i.e., the USEPOPINFO model was turned off by setting popflag = False in the StrAuto parameters). This ensures that the resulting clusters were defined solely by the genetic signal, independent of geographic sampling labels. We have updated the Methods (**Lines 737–739**) to explicitly state these parameters.

• **Line 161: No species were mentioned before. How many are there in the section, and how many were worked with? See Line 163, which is a bit uncomfortable. How was the determination made when it was not an aim of the study, and the authors claim that more work needs to be done? From the Supplementary material, which is largely species-based, it seems that there is no need for additional study.**

Reply: Regarding the question of "how many species", we have addressed this by adding a dedicated paragraph to the Introduction (**Lines 92–103**) detailing the taxonomic controversy in *Stenopoa*, where estimates range from ~15 to >100 species depending on the authority. Our study included 37 morphologically defined taxa, representing the section across Eurasia.

The species determinations in the Supplementary material were initial morphological identifications performed by Prof. M. Olonova. These names served as the hypotheses to be tested against the genomic data.

We respectfully disagree with the suggestion that the Supplementary material implies "no need for additional study". As detailed in Supplementary Table 9, we found significant incongruence between these morphological names and the genetic clusters: (1) 80 out of 373 individuals were reassigned based on molecular evidence; (2) we identified only 21 robust genetic units compared to the 37 morphospecies; and (3) one taxon, *P. litvinoviana*, appeared to be polyphyletic. Consequently, the taxonomic status of these morphospecies remains unresolved. A formal revision (synonymising names or validating species) will require analysis of type specimens, detailed cytogenetics, and higher-density SNP data. We have updated the Results (**Lines 185–188**; **303**) and Discussion (**Lines 617–624**) to explicitly address these discrepancies and the need for future taxonomic work.

• Line 167: There were 373 individuals in Line 124. It is unclear how that became 52 in line 164 and is used for n in line 167. Please clarify. Note that the manuscript has the M&M section at the back, so the text should reflect that.

Reply: We agree that the transition from the total dataset (n = 373) to the subset used for dating (n = 52) was abrupt in the original text. To clarify:

- 373 is the total number of genotyped individuals used for the global population structure (Clusters I, II, and III).
- 52 is the strict subset of "genetically pure" individuals identified via our hierarchical clustering strategy (Methods; **Lines 753–758**). This subset was selected to minimise violations of model assumptions (e.g., gene flow mimicking incomplete lineage sorting) specifically for the SNAPP time-tree analysis and the morphometric analysis.
- n = 13, 10, 29 refers to the number of pure individuals within each Cluster (totalling 52) used as the input for SNAPP.

We have revised the Results section (**Lines 185–188**) to explicitly describe this filtering step before introducing the dating results.

• Line 173: It is not clear whether the groups 18-21 belong to Cluster I. Same for groups 12 and 14. Maybe switching the lines 174-179 with details about geographic distribution, with lines 170-173 containing some taxonomic details, would help.

Reply: We agree that the original ordering caused confusion regarding the cluster assignment of specific groups (e.g., 18–21). We have adopted the reviewer's recommendation to switch the order (**Lines 196–206**).

• Fig. 2: The figure is nice and illustrative, but so condensed that the text is very difficult to read. The bootstrap, Population codes and PCA pie charts became readable in 6x Zoom, the x-axis in UPGMA in 12x Zoom, and the tip numbers and map pie charts in 3x Zoom, which makes them all hard to find and redundant. I wonder if there is a way to enlarge the overall figure by putting, e.g. the PCA with the Fst inset on the side. It is not scaled with the map anyway. In addition, I wonder if it is possible to mark the admixed samples in the Neighbor Net pane in some shade of violet/pink/purple, similarly to the PCA plot.

Reply: We agree that the high information density makes the text difficult to read at standard page size. However, given the large sample size (n = 373), enlarging individual labels directly in this composite figure would result in significant overlapping, compromising the clarity of the overall genetic structure. We emphasise that Figure 2 is intended as a global overview of the dataset structure, whereas Figure 3 provides a focused and legible view of the specific genetic groups and taxonomic labels. Changes made:

- As suggested, we have modified the Neighbor-Net pane to colour-code the admixed samples using shades consistent with the PCA plot.
- We have provided high-resolution vector figures via Figshare (Data Availability, **Line 914**) to allow readers to zoom in without loss of quality.

• Line 267: While the conclusion about the two most powerful discrimination traits is tempting, the wording suggests that the success of the adaptation does not depend on fitness but on trait size. In contrast to this, plants with short panicles may be well adapted to their environments if they have greater survival and fitness, and it may not depend on panicle length. Please clarify.

Reply: We agree that "adaptation" should not be equated with "maximisation" of a trait size, but rather with the optimisation of that trait for specific environmental constraints to maximise fitness. For example, as the reviewer notes, the reduced panicle size and stature in Cluster III likely represent a fitness advantage (stress tolerance/survival) in arid, high-altitude environments, whereas the larger traits in Cluster I reflect an adaptation to competitive, resource-rich environments. We have revised the text (**Lines 297–300**) to clarify that these traits reflect divergent adaptive strategies and functional trade-offs, rather than implying a linear relationship between trait size and success.

• Line 272: Despite the taxonomic revision not being an aim of the study, it would be worth noting how many species are in the clade and how many species were successfully or unsuccessfully assigned. In addition, while gene flow is acknowledged between the clades, the possibility of some species being of a hybrid origin should also be mentioned. IN addition, Supplementary Table 9 shows a species name for every sample, not only 80 samples as the text suggests. Please clarify and explain how the species determination was carried out. It would be helpful to keep both, Herbarium but al so manuscript ID so that the individuals can be found out without having to consult another Supplementary Table.

Reply: We have updated the text:

- To clarify that our study analysed 37 morphospecies, identifying 21 cohesive genetic groups. We explicitly state in the Results that 80 individuals (21.45%) were reassigned based on molecular evidence (**Line 303**).
- We acknowledge the role of hybridisation in the Discussion (**Lines 617–624**). Additionally, Supplementary Tables 1, 9, and 15 now explicitly categorise "Admixed" taxa/individuals to highlight those with putative hybrid genomic signatures.
- Initial morphological determinations were performed by Prof. M. Olonova using standard taxonomic keys. Regarding Supplementary Table 9, we list all samples to provide a complete dataset of morphological vs. molecular identifications, allowing readers to verify which samples matched and which did not (the 80 reassigned).
- To facilitate cross-referencing without consulting multiple tables, we have updated Supplementary Table 4 to include the Species Name alongside the Figure 2 ID and Herbarium ID.

• Fig. 4: Boxplots for 15 characters are too small to be informative. Do the points in the plots represent the genetically pure individuals? It is unlikely to be characters as the caption for 4a reads. I suggest splitting the Figure into three parts, having the character section together with the illustration as a separate pane (c).

Reply: We appreciate the reviewer's feedback on the improvement of the figure. We offer the following clarifications:

- We confirm that the points in the boxplots represent the 52 genetically pure individuals used in the morphometric analysis, as defined in the Results (**Line 238**) and Methods (**Line 774**).
- We carefully considered the suggestion to split the figure into three panes. However, we chose to retain the current layout because the boxplots are explicitly ordered by their contribution to the PCA Dimensions shown in Panel A. Separating the illustrations or boxplots into a third pane would break this logical link between the multivariate analysis and the specific trait contributions.
- To address the visibility concern, we have provided high-resolution vector figures via Figshare (Data Availability, **Line 914**). These allow readers to zoom in on specific boxplots to see distribution details without loss of quality. Additionally, Supplementary Figure 2 provides larger, individual notched boxplots for all studied morphological variables for detailed inspection.

• Fig. 5: Font size too small to be comfortable to read.

Reply: We have increased the font size where spatial constraints allowed without causing overlap. Furthermore, as with Figure 4, the provision of vector-based images ensures that all text remains sharp and legible when zoomed in (Figshare, Data Availability, **Line 914**).

• Line 405: I feel like the sentence starting with "Shared habitats…" does not link well with the previous and next sentences. Please clarify.

Reply: We have revised the text (**Lines 437–439**) to provide a stronger logical link. We now explicitly introduce the finding about shared habitats as a specific example of the integrated insights mentioned in the previous sentence.

• Line 435: The mention of polyploidy is completely out of place here. Polyploidy was not included in any of the analyses or presented separately. In the whole manuscript, polyploidy was only mentioned in the Introduction and in a very general way. Thus, I strongly suggest either incorporating polyploidy into the study or rewording any bold statements about the role of polyploidy in the evolution of the study clade. Are there any ploidy data the authors could use? Were the genetic data analysed with respect to different cytotypes? Is there any cytogeography pattern or presence in polyploids in the clades? While the Supplementary Table X provides an overview of the chromosome counts, the Table also clearly shows many aneuploid counts. Given that the chromosome counts are more or less equally distributed among the Clusters, why do authors think polyploidy plays any role in the clade evolution? While allopolyploidy is acknowledged, what about autopolyploidy and polyploid formation in harsher environments? This clade seems to be an ideal candidate for the study of the ratio of unreduced gamete formation in stressful environments.

Reply: We appreciate the reviewer's detailed critique regarding the presentation of polyploidy. We agree that in the original manuscript, the discussion of polyploidy appeared disconnected from the results. We have revised the manuscript to integrate this aspect more rigorously:

(1) We have added a "Cytological data compilation" section to the Methods (**Lines 904–909**) and a new "Cytological variation" section to the Results (**Lines 462–472**). This explicitly presents the literature-based chromosome counts (summarised in Supplementary Table 15) as part of our dataset.
(2) We have reworded the introductory statement in the Discussion (**Line 482**) to present polyploidy as the evolutionary context of the lineage rather than a variable we directly tested.

(3) We thank the reviewer for highlighting the role of unreduced gametes in harsh environments. We have rewritten the relevant Discussion paragraph (**Lines 531–553**) to explicitly address this.

We acknowledge that the equal distribution of chromosome counts across clusters suggests that polyploidy itself is not the sole driver of the divergence between Clusters I, II, and III. Instead, we argue that the "widespread cytotypic complexity" (including aneuploidy/dysploidy and unreduced gametes, as suggested by the reviewer) provides the genetic redundancy and plasticity required for all these lineages to persist in harsh environments. We now explicitly cite the role of unreduced gametes and facultative apomixis alongside hybridisation as key evolutionary mechanisms in this group.

• Line 436: Any note about the economic significance of this lineage is missing in the Introduction, so it is surprising to find it in the Discussion. It feels out of place and context there. Please rewrite.

Reply: We agree with the reviewer that the mention of economic significance was not established in the Introduction and appeared out of context. We have removed the phrase "and economically" from the Discussion (**Line 483**) to maintain focus on the ecological and evolutionary significance of the lineage, which is the central theme of the manuscript.

• Line 444: Are there any particular adaptations to aridity? While morphological results were shown, no adaptive traits were mentioned or later discussed. This is a similar case to the polyploidy: stating that the plant is polyploid and adapted without showing how is too strong a statement for a discussion, where it implies it is a result of the presented manuscript. In contrast, studying adaptation or polyploidy was not included in the study, and no details about adaptive mechanisms or ploidy levels are mentioned in the Results, Materials section or Introduction (for the adaptive mechanisms). Please rewrite with respect to the fact that some information about adaptation is provided two paragraphs later (line 463), and polyploidy is briefly discussed in line 483. Regarding polyploidy again, any discussion about unreduced gametes formation under harsh conditions would nicely fit line 514 and further. It should also be mentioned that there are studies comparing the presence of diploids and polyploids in formerly glaciated areas, which may help your discussion about the clade evolution during the glacial cycles.

Reply: We agree that the term "adaptations" was used too broadly in this introductory paragraph without immediate reference to the specific traits measured. We have revised the text to be more precise and to incorporate the suggested evolutionary context:

(1) We updated the Discussion (**Lines 491–492**) to replace the vague phrase "adaptations to aridity" with specific morphological traits.
(2) We have incorporated the reviewer's suggestion regarding the distribution of cytotypes. We added a statement (**Lines 569–572**) highlighting that polyploids often exhibit superior colonisation abilities in formerly glaciated landscapes compared to diploids, which supports our demographic inference of post-glacial expansion for the polyploid-rich clusters.
(3) We have explicitly integrated the discussion of unreduced gametes and facultative apomixis into the Polyploidy section of the Discussion (**Lines 539–545** & **550–553**), framing *Stenopoa* as a candidate system for studying these mechanisms in stressful environments.

• Line 566: This sentence feels very redundant, given that the work did not acknowledge the existence of polyploids in any of the analyses.

Reply: We agree that the original phrasing felt redundant and overstated, as we did not explicitly test cytotypes. We have revised the sentence (**Lines 637–639**) to reframe this as a future research priority.

• Line 575: Please explain how your findings can suggest anything about the role of polyploidy when polyploidy was not part of the study, and no ploidy patterns were presented. The only mention of polyploidy is that there are overlapping chromosome counts presented in all clusters, which does not show or support any role of polyploidy.

Reply: Following this and the reviewer's previous comments, we have integrated the available cytological data (presented in Supplementary Table S15) more rigorously into the manuscript structure:

- We added a new section "Cytological data compilation" to the Methods (**Lines 904–909**).
- We added a new section "Cytological variation" to the Results (**Lines 462–472**).

Consequently, we have revised the Discussion (**Lines 531–545** & **550–553**). Instead of implying a direct causal link between polyploidy and diversification that was not explicitly tested, we now discuss the observed cytotypic complexity as a factor that likely provided the necessary genetic redundancy for survival, framing the specific mechanistic role of polyploidy as a hypothesis to be verified in future studies.

• Line 578: The last sentence of the Discussion is in contrast with the previous findings' summary throughout the manuscript. While it may not be untrue, it is a very dense shortcut that should be elaborated and reflect on the actual presented results, e.g. from line 464 (morphological traits driving phenotypic divergence) and line 480 (morphological evolution as a direct adaptive trait to distinct ecological niches and climatic pressures therein). If polyploidy and hybridisation play any role in providing genetic redundancy and buffer against environmental conditions (paraphrasing line 515 here), it should be an aim of future research, together with detecting the genomic basis of Stenopa (likely morphological) adaptation. It would also be worth elaborating on how exactly this study contributes to the broader understanding of climate change effects on plant evolution (line 579).

Reply: We have rewritten the Conclusion (**Lines 646–655**) to reflect the actual results presented in the paper. As suggested, we have shifted the topics of polyploidy buffering and the genomic basis of adaptation from "conclusions" to "critical aims for future research".

• Line 585: How many populations were used? Is each population represented by one individual?

Reply: As this study relied primarily on historical herbarium vouchers collected over 47 years to maximise geographic coverage across Eurasia, we did not employ a traditional population-genetic sampling design (e.g., collecting 20 individuals per site). Instead, most specific localities are represented by single individuals. Consequently, for the demographic and genetic analyses, "populations" were not defined *a priori* by collection site. Instead, we defined populations *a posteriori* based on the robust genetic Clusters (I, II, and III) consistently recovered by the joint application of STRUCTURE, PCA, and Neighbor-Net analyses. This approach allows for the analysis of evolutionary units without the bias of arbitrary geographic grouping. We have updated the Methods section to explicitly state this strategy (**Lines 662–666**).

• Line 590: The double digest restriction-site associated DNA genotyping term usually refers to ddRADseq (Peterson et al. 2012), not DArT (Killian et al. 2012). While both methods use restriction enzymes to reduce the DNA representation, the library preparation and data format are very different. The authors should clearly explain the differences between DArT and ddRADseq and why they use the term coined for another method. In addition, since the Killian et al. 2012 protocol is no longer in use, the authors should provide a relevant reference. Note that the Methods description should be self-standing and reproducible. Using a misleading term for the methods together with an outdated reference obviously is not.

Reply: We apologise for the confusing terminology. DArTseq is indeed similar to ddRAD but has the advantages of accepting lower quantities of DNA, greater tolerance of lower quality DNA and yielding lower allelic dropout rates (Sansaloni et al., 2011; Georges et al., 2018). We have replaced the confusing terminology throughout the manuscript with DArTseq genotyping (Introduction, **Line 118**; Methods, **Line 671**).

Regarding the Kilian et al. (2012) reference, we consulted with the DArT company, who confirmed that this remains the seminal paper describing the underlying principles of the digestion/ligation complexity reduction method. Crucially, this reference is not outdated and remains the standard citation for this platform in high-impact research, as demonstrated by recent studies (e.g., Georges et al., 2018; Alves et al., 2023; Wróbel et al., 2024). Additionally, we explicitly cite Sansaloni et al. (2011; **Line 682**) and Elshire et al. (2011; **Line 685**) in the Methods to reference the specific adapter and barcode designs used in this protocol.

Reference

Alves, F., Banks, S.C., Edworthy, M. et al. Using conservation genetics to prioritise management options for an endangered songbird. *Heredity* **130**, 289–301 (2023). https://doi.org/10.1038/s41437-023-00609-6

Georges, A., Gruber, B., Pauly, G.B. et al. Genomewide SNP markers breathe new life into phylogeography and species delimitation for the problematic short-necked turtles (Chelidae: *Emydura*) of eastern Australia. *Molecular Ecology* **27**, 5195–5213 (2018). https://doi.org/10.1111/mec.14925

Wróbel, A., Klichowska, E. & Nobis, M. Hybrids as mirrors of the past: genomic footprints reveal spatio-temporal dynamics and extinction risk of alpine extremophytes in the mountains of Central Asia. *Frontiers in Plant Science* **15**, 1369732 (2024). https://doi.org/10.3389/fpls.2024.1369732

• Line 605: Was there any size selection? Why or why not? Why were so many PCR cycles, possibly introducing sequencing errors, used? What was the length of the sequenced fragments?

Reply: We have consulted with the DArT company to confirm the specific parameters used for this project:

(1) Yes, size selection was performed. The library preparation targeted fragments in the 100–300 bp range. As noted in our response to Reviewer #2, this aids in the physical exclusion of large, uncut chloroplast DNA fragments and optimises cluster density on the Illumina flow cell.

(2) Regarding PCR cycles, while high, this is the standard DArT workflow for projects involving historical herbarium specimens, which often yield low-concentration or degraded DNA. The DArTseq analysis pipeline relies on clustering identical reads; random PCR errors typically occur at low frequencies and fail to form sufficiently large clusters to be called as reference

tags. Furthermore, we applied a strict filtering threshold (100% reproducibility) to eliminate non-replicable artifacts (**Line 722**).

(3) Sequencing was performed on Illumina NovaSeq 6000 or NovaSeq X platforms (single-end). While raw read lengths varied by machine batch (83 or 138 bp), sequences were trimmed to approximately 69 bp to ensure consistent marker calling across the.

We have updated the Methods section (**688–692**; **697–698**) to include these specific parameters, ensuring the description is self-standing and reproducible.

• Line 600: What is a DArT PL script? A reference or an explanation why it is not provided is needed. A newer reference for DArT than Killian et al. 2012 should be provided, given the differences between the initial DArT and the method the authors (and the company) used.

Reply: The phrase "DArT PL script" referred to an internal automation protocol used by the DArT Company (Diversity Arrays Technology Pty Ltd, DArT PL). To avoid confusion with common programming extensions (e.g., .pl for Perl), we have removed this specific phrasing and replaced it with a clear description of the procedure: "performed on the T100 using a 96-tip head and automated liquid handling steps" (**Lines 680–681**).

Regarding the Kilian et al. (2012) reference, as noted in our response above, this remains the most detailed "methods" paper the DArT team produced. Crucially, this reference is not outdated in terms of current usage. As demonstrated by recent studies high-impact journals (e.g., Georges et al., 2018; Alves et al., 2023; Wróbel et al., 2024), Kilian et al. (2012) remains the standard citation for this platform.

References

Alves, F., Banks, S.C., Edworthy, M. et al. Using conservation genetics to prioritise management options for an endangered songbird. *Heredity* **130**, 289–301 (2023). https://doi.org/10.1038/s41437-023-00609-6

Georges, A., Gruber, B., Pauly, G.B. et al. Genomewide SNP markers breathe new life into phylogeography and species delimitation for the problematic short-necked turtles (Chelidae: *Emydura*) of eastern Australia. *Molecular Ecology* **27**, 5195–5213 (2018). https://doi.org/10.1111/mec.14925

Wróbel, A., Klichowska, E. & Nobis, M. Hybrids as mirrors of the past: genomic footprints reveal spatio-temporal dynamics and extinction risk of alpine extremophytes in the mountains of Central Asia. *Frontiers in Plant Science* **15**, 1369732 (2024). https://doi.org/10.3389/fpls.2024.1369732

• Line 612: Even if proprietary, the authors still should refer to the software, server, company, etc.

Reply: We have updated the Methods (**Line 697**) to specify that the processing was performed using pipelines developed by Diversity Arrays Technology Pty Ltd (Canberra, Australia).

• Line 622: A reference for the DArTsoft14 should be provided.

Reply: We consulted with the DArT company regarding a specific citation for DArTsoft14. They confirmed that this software remains a proprietary technology and has not been published in detail to protect intellectual property; therefore, no bibliographic reference exists. To address the reviewer's request for proper attribution, we have updated the text to cite Diversity Arrays Technology Pty Ltd (Canberra, Australia) as the source of the software (**Line 708**).

• **Line 625: "Using a range of parameters" is a very vague statement. Provide details.**

Reply: We have consulted with the DArT company to obtain the specific parameter values used in the DArTsoft14 pipeline. We have updated the Methods to explicitly state that clustering was performed at a threshold distance of 3 (corresponding to approximately 95% sequence identity) and SNPs were called using a minimum allelic balance of 5 (**Lines 710–711**).

• **Line 631: Provide the version and a reference for R.**

Reply: Revised (**Line 720**).

• **Line 648: Explain the low number of STRUCTURE iterations for burn-in (10,000) and MCMC chains (100,000). It is common to have a burn-in of 100,000 and MCMC of 1,000,000 iterations. I am reluctant to accept conclusions relying on what is a burn-in phase in most other studies.**

Reply: We understand the reviewer's concern, as longer run lengths (e.g., 1,000,000 iterations) are often used as a default precaution in population genetics. However, the critical criterion for ensuring the validity of Bayesian clustering is not the raw number of iterations, but the convergence of the MCMC chains to a stationary distribution. For our dataset, the genetic structure between the clusters was sufficiently distinct to allow for rapid convergence. We assessed this by visually inspecting the trace plots of key parameters (such as Alpha) across independent runs. These diagnostics confirm that the chains reached a stable plateau (stationarity) well within our burn-in phase and remained stable throughout the sampling phase. We have provided interactive diagnostics (HTML format) for all STRUCTURE runs in Supplementary Data 1 (folder STRUCTURE; available via Figshare in the Data Availability section, **Line 914**). For the reviewer's convenience, we present the convergence plot for the main analysis (K = 3) below (Figure 1), which demonstrates that stationarity was consistently achieved. Additionally, please see our reply to Reviewer#3 below.

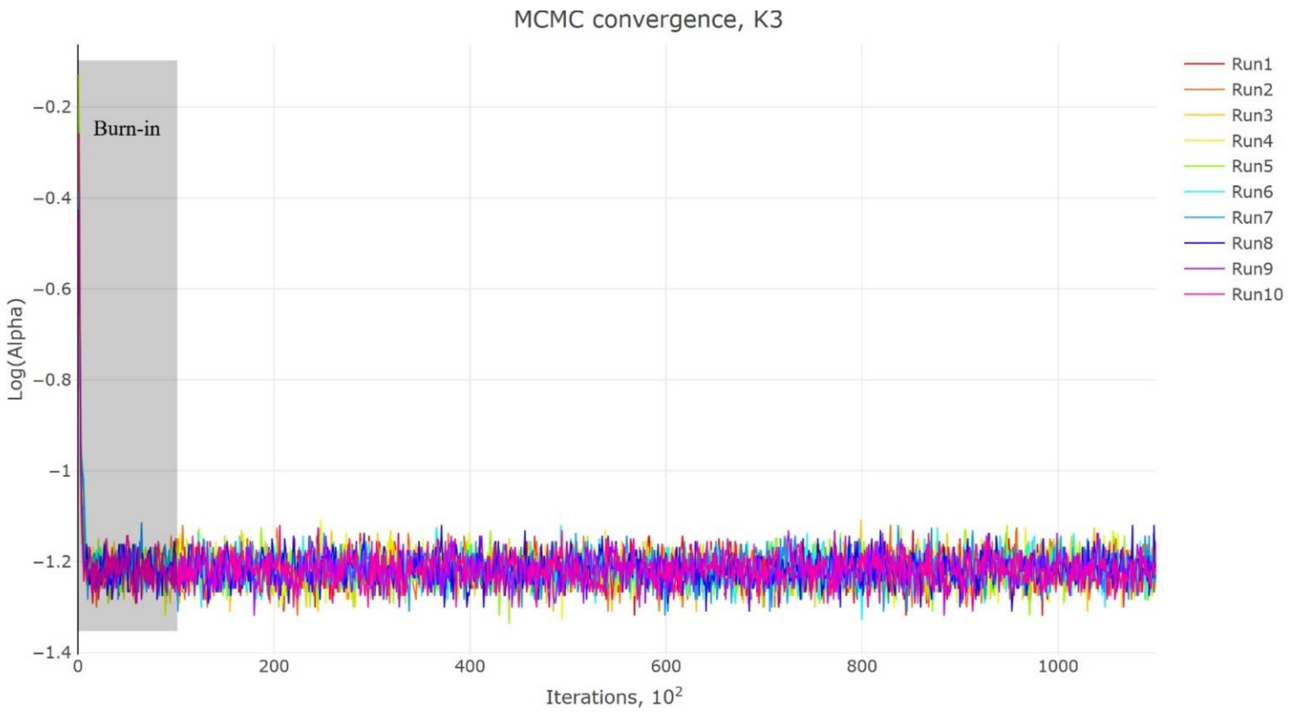

Figure 1. MCMC convergence diagnostics for STRUCTURE at K=3. The X-axis represents the number of MCMC iterations, while the Y-axis represents the log-transformed Alpha (α) parameter. Visual inspection of the ten independent runs (coloured lines) confirms that the chains reached a stable plateau/convergence.

• Line 672: I am not sure "structure" is the right word.

Reply: We agree that "structure" was too vague in this context. We have replaced it with the more precise term "Morphometric analysis" in the revised manuscript (**Lines 236 & 772**).

• Line 674: What was the ratio of individuals per cluster? Were the clusters equally represented?

Reply: The morphological dataset was restricted to the subset of individuals confirmed as "genetically pure" ($Q \geq 0.99$) by STRUCTURE. This resulted in the following distribution: Cluster I ($n = 13$), Cluster II ($n = 10$), and Cluster III ($n = 29$). We have updated the Morphometric analysis section (**Line 774**) to explicitly state these sample sizes.

• Line 713: DArT protocol sequence length should also be mentioned in the sequencing section of the Methods (see my related comment).

Reply: We have updated the Methods (**Line 691**) to explicitly state that sequencing was performed using single-end reads of 83 bp or 138 bp (depending on the batch), which were subsequently trimmed to 69 bp for marker calling (**Line 698**). This ensures the description is consistent with the parameters used for demographic inference (**Line 817**).

• Line 724-733: This paragraph should follow after the first mention of the two datasets in the first paragraph of this section, keeping the information about the datasets together.

Reply: We agree that the organisation of the section was disjointed. We have restructured this section to consolidate the information about the datasets:
(1) We have combined the description of the two datasets (70% and 95% call rate) into the first paragraph, immediately following the mention of the software (**Lines 810–813**).
(2) While the Stairway Plot 2 analysis remains first, we have clarified the specific rationale for the dual-dataset approach to the GADMA-specific paragraph (**Lines 834–849**). This change ensures that the text accurately reflects that AIC-based model selection is a component of the GADMA workflow rather than the Stairway Plot analysis.

• Line 734: How many populations were used and how many individuals were sampled per population? If you mean clusters by populations (as in line 738), explain it in Line 734, at the first mention of the populations in the manuscript, to avoid confusion and enhance clarity.

Reply: We have revised the text to explicitly state that the "populations" used in the study correspond to the three genetic clusters (I, II, and III). We now define this terminology earlier in the "Plant material and sampling strategy" section (**Lines 662–666**) and reiterate it at the start of the demographic inference section (**Line 810**) to ensure clarity. Additionally, we refer to Supplementary Table 2 for the exact number of individuals per cluster for each dataset version.

• Line 738: What is the difference between the first two models from the three models mentioned in this line and the two models referred to in Line 734?

Reply: We have revised this section to clarify the distinction between the pairwise and joint analyses (**Lines 834–846**). Specifically:
(1) The pairwise models were used to determine the best-supported mode of gene flow (continuous "isolation with migration" vs. episodic "secondary contact") for each cluster pair separately.
(2) The three-population models subsequently integrated these results into three joint scenarios: (A) continuous gene flow between all population pairs; (B) combination of isolation with

migration and secondary contact; (C) admixture model designed to explicitly test the formation of Cluster III via an admixture event between Clusters I and II.

- **Line 751: Does this mean that the genetic dataset contains duplicates and erroneous entries?**

  Reply: The term "records" in this context refers specifically to the geographic occurrence coordinates used for MaxEnt modelling, not the SNP data itself. The "cleaning" described involved removing spatial duplicates (multiple samples collected from the same or very close geographic coordinates). To address this and minimise spatial autocorrelation, we applied spatial thinning to reduce redundant records within a 1 km² grid. To avoid further ambiguity, we have removed the word "erroneous" and revised the sentence (**Line 856**).

- **Line 754: If only 143 individuals were used for the modelling, how many of the remaining (373-143 =) 230 individuals were removed due to spatial thinning and how many were removed during the cleaning? How were the genetically admixed samples handled if the modelling was cluster-based and there were only 52 genetically pure individuals that could be unambiguously assigned to each cluster?**

  Reply: We apologise for the confusion regarding the sample sizes. To clarify the filtering workflow:

  (1) From the total 373 genotyped individuals, 174 were excluded because they were genetically admixed according to our global molecular analyses. This left a total of 199 "pure" individuals available for ecological modelling (Supplementary Table 1). The 52 "pure" individuals mentioned elsewhere refer to a strict subset of these 199, selected via our stepwise strategy to identify stable genetic groups for the SNAPP phylogeny (as we now define in the Methods, **Lines 753–759**). However, for SDM, we utilised the full set of 199 pure individuals to maximise geographic coverage.

  (2) From these 199 pure occurrences, 56 records were removed during the data cleaning (removing exact duplicates) and spatial thinning (1 km² grid) steps to minimise spatial autocorrelation.

  (3) The final dataset contained 143 spatially unique occurrences (Supplementary Table 11) used for the MaxEnt modelling.

  We have revised the first paragraph of this section to clarify our strategy (**Lines 853–860**).

- **Line 759: Keep shortcuts consistent with the main text; use either Kya or ka throughout the text.**

  Reply: Corrected. The revised **Lines 865 & 867.**

- **Line 760, 762: Refer to the databases consistently, including the references.**

  Reply: We have revised the text to ensure consistent citation of database versions (WorldClim v.2.1, WorldClim v.1.4, and PaleoClim v.1.0) and their associated primary references (**Lines 866–867**).

- **Supplementary Table 2: The table lists the same analyses with a different number of individuals without providing any details, which makes it rather useless. For example, there are STRUCTURE (Cluster III) analyses with either 113, 18, 12, 9 or 7 individuals. What are we supposed to do with this?**

  Reply: We apologise for the lack of explanatory details in this table. These rows document the hierarchical, stepwise analysis we performed to define the final genetically pure groups

(visualised in Supplementary Figure 1), as well as the associated downstream analyses (Stairway Plot 2, GADMA2, and SNAPP). The variation in individual numbers represents different levels of this hierarchy:

(1) The initial analyses performed on all samples (n=373)
(2) Sub-structured analyses within the three main clusters (Cluster I, n=55; Cluster II, n=31; Cluster III, n=113). For example, the analysis of 113 individuals represents the investigation of the entire Cluster III
(3) The analyses of smaller subsets (e.g., 18, 12, 9, or 7 individuals) represent specific sub-lineages within Cluster III. We analysed these groups individually to test for further internal substructure and to confirm they constituted stable evolutionary units before including them in the SNAPP analysis

We have updated Supplementary Table 2 by adding an "Analysis Stage" column that links these subsets to the specific steps of the hierarchical analysis and the final genetic groups (1–21) identified in Figure 3. We have also expanded the table caption to explain this workflow.

• **Supplementary Table 4: This table has an unfortunate format. If kept separately, condense it into multiple rows. However, if possible, incorporate the information about ID and Herbarium number into the relevant Supplementary Tables S9 and S11.**

Reply: While we considered merging this information into Table 9 or S11, we found that doing so overcrowded the master table. Therefore, we have opted to retain and improve Supplementary Table 4:

(1) We have reformatted the table into multiple columns to significantly reduce its length, addressing the formatting concern
(2) We have incorporated the final molecular species assignments (from Table 9) into this table. This allows readers to quickly identify the taxon associated with every number in Figure 2 without needing to cross-reference multiple tables

• **Supplementary Table 5 and S6: Please condense into one table.**

Reply: We carefully considered the suggestion to condense Supplementary Tables S5 and S6. However, we have chosen to keep them separate to maximise readability and utility for the reader. Table 5 serves as an alphabetical glossary of the morphological characters and their abbreviations, allowing for quick reference. Table 6 presents statistical results (PCA contributions) and is sorted by the magnitude of contribution to highlight the most discriminatory traits (top contributors). Merging these tables would require compromising on the sorting order (either making definitions hard to find or scrambling the rank order of PCA contributions). Therefore, we believe maintaining them as distinct tables is the most reader-friendly approach.

• **Supplementary Table 8: Please add full character name – plenty of space there.**

Reply: Corrected.

• **Supplementary Table 11: What is Raster ID?**

Reply: "Raster ID" represents the unique numeric index of the 2.5 arc-minute (ca. 5 km) grid cell within the standardised environmental raster layers (WorldClim/PaleoClim). It identifies the specific environmental pixel corresponding to each sample's coordinates. This ID was utilised during the data preparation to ensure that only one occurrence record per environmental grid cell was retained for the MaxEnt modelling, thereby minimising spatial autocorrelation. We have added a footnote to Supplementary Table 11 to clarify this definition for the reader.

• Supplementary Table 15: The format is unfortunate. CCDB is a secondary source with references to the primary source. The primary source always has to be checked due to the way the database was (automatically) assembled. Thus, this column should be incorporated in the reference column. The column before the references misses a headline. Are those chromosome numbers coming from different sources than the ones used in CCDB? What does the section Other represent if the Stenopoa clade is represented by three clusters revealed in this study? Please clarify. While polyploidy refers to whole-genome duplication, chromosome numbers outside the multiplication of the basic chromosome number (7) indicate other processes that should also be discussed in the text. Given the variation in chromosome numbers equally distributed among the clusters, what would be the role of polyploidy (assuming taxa with aneuploid chromosome numbers are polyploids of some kind)? Is polyploidy only a result of a hybridisation (allopolyploids origin from the secondary contact), or is there another possible explanation, such as higher unreduced formation in harsh environments leading to autopolyploids and aneuploids?

Reply: We agree that the original format was confusing and have revised Supplementary Table 15 and the associated text to address these points:

(1) We have restructured the table by removing the "CCDB" column. We traced the primary literature for these counts and cited the original authors in the reference column.
(2) We clarified that the column previously missing a header represents the 2n chromosome count.
(3) We double-checked the References, removed redundant literature, and formatted them according to the Main text style.
(4) We renamed the "Other" section to "Admixed taxa" to maintain consistency with Supplementary Tables S1 and S9. These entries represent species (e.g., *P. compressa*) found to be genetically admixed in our global STRUCTURE analysis (Main text, Fig. 2), distinguishing them from the taxa forming the three primary genetic clusters.

Additionally, we appreciate the reviewer's insight regarding the non-euploid counts (numbers outside multiples of x = 7). We agree that these counts indicate processes beyond simple WGD. We now address this point in the Discussion (**Lines 539–545)**.

• Supplementary Figure S7: These are likely not histograms, but a distribution. Clarify the headline.

Reply: Corrected.

##

Referee #2: Evolution and breeding system variation in genus Poa

Reviewer #2 (Remarks to the Author):

###

• Pleistocene adaptation and diversification in cold, arid environments: genomic and palaeoclimate insights from non-model bluegrasses

• This is an interesting analysis of Poa sect. Stenopoa complexes, and fairly novel at that.

• The paper seems to be very well written and intelligible. Aside from too many acronyms to keep track of says this blurry eyed reviewer.

> **Reply:** Thank you for your positive feedback. We are delighted to hear that you found the paper well-written and intelligible. Regarding the number of morphological terms, we recognise that the text is acronym-heavy; however, we provide the full form of all abbreviations both upon their first use in the text and in Supplementary Table 5. We hope this makes the paper easier to follow.

• The English is clear and natural.

• Ligula should be corrected to Ligule

> **Corrected.** Revised (**Lines 266** & **313**; Supplementary Table 5).

• The analytical methods appear to be sound. Cluster analyses and PCA are widely familiar.

• This reviewer is not versed in the other statistical methods of analyses employed.

• The manuscript covers a lot of ground with other statistics but seems appropriate in length for all of that.

• The figures are well done and appropriate.

• The discussion of the geography of climate zones, growing season lengths and timing, and ppt patterns and amounts and corresponding morphology is useful. This reviewer doesn't understand how this relates to population sizes (bottlenecks?).

> **Reply:** We appreciate the reviewer's positive assessment of the morphological and climatic analyses. To clarify the relationship to population sizes: we interpret the climatic variables (precipitation patterns, temperature, and seasonality) as the primary determinants of the spatial extent of suitable habitat. Our data suggests that while unfavourable climates (e.g., LGM; 21 Kya; Fig. 6c) caused severe spatial range contraction and fragmentation, the specific geography of the climate zones, specifically the stable refugia in the Russian Far East and Tibetan Plateau, buffered the species against severe demographic bottlenecks. This explains how lineages persisted (maintained effective population sizes) despite the glacial conditions. We address this connection in the Discussion (**Lines 563–566**), where we note that these regions facilitated "persistence during glacial maxima", and in **Lines 577–585**, where we discuss how the spatial fragmentation drove isolation rather than extinction.

• The analysis of gross morphological vegetative measures used in PCA Dimensions is interesting also. And well done. This reviewer noticed the absence of pubescence characters of lemmas and calluses (often important for taxon separation in Poa), and absence of characters of prickle hairs "p/a", and their direction (antrorse versus retrorse) below stem nodes which Tzvelev (2009) in particular, considered of high importance for taxon discrimination, and found to be quite useful by the present reviewer. A comment on why these traits were ignored would be appreciated. Basically, because they are qualitative, not quantitative. This study could not have been done without Olonova's extensive and careful scoring of morphological variations on individual specimens and identifications.

> **Reply:** We appreciate the reviewer's recognition of the taxonomic effort involved. Prof. M.V. Olonova indeed assessed these qualitative traits (rachilla pubescence, lemma callus pubescence, and pubescence between veins of lemmas). However, our initial assessment indicated that these traits were highly variable and did not consistently distinguish the broad genetic clusters identified in this study. Therefore, as the reviewer correctly surmised, we retained only quantitative traits for the final statistical analyses.

• It is interesting that the authors were able to identify a few speciation gaps and peaks corresponding to peak glacial periods (spanning up to seven interglacial periods).

• The DNA extraction method would seem to favor Chloroplast DNA over nuclear DNA, since plastid DNA is in many thousands of copies compared to nuclear DNA, beyond nuclear ribosomal repeats. PSTI And HpaII are infrequent restriction site cutters of chloroplast DNA. Was there an attempt to distinguish plastid from nuclear DNA or single copy nuclear DNA from repeat nuclear ribosomal DNA? If they could factor for this, I wonder they could estimate the data contribution from plastid versus nDNA or nrDNA contributions to the cluster analyses, or if chloroplast DNA could be screened for, in or out, in the subsequent analyses for SNPs. Plastid DNA inheritance in Angiosperms is rarely from pollen parents. Were they able to detect ploidy levels in samples?

> **Reply:** We have contacted the DArT company to clarify this. *PstI* and *HpaII* are indeed infrequent cutters of chloroplast DNA and the most effective enzyme combination for enriching genic regions in large grass genomes. In the DArTseq protocol, this specificity is combined with a strict size selection step targeting 100–300 bp fragments (we have updated the Methods, **Lines 688–690**). Consequently, plastid DNA is physically excluded because the resulting fragments are too long to be amplified and clustered on the flow cell. Furthermore, the *PstI-HpaII* combination targets the unmethylated gene space, which effectively filters out the highly methylated nuclear repetitive fraction (including nrDNA). Therefore, the exclusion was achieved through the specific restriction chemistry and size selection, ensuring the dataset represents the low-copy nuclear genome; estimating a data contribution from plastid versus nDNA/ nrDNA is not applicable as plastid sequences are effectively absent.
>
> Regarding ploidy detection, as detailed in our response to Reviewer #3, we were unable to reliably detect ploidy levels in these samples. The DArTseq read depth (typically <30x for most markers) is insufficient for the dose-calling required to estimate ploidy *in silico*, and the nature of the herbarium material precluded flow cytometry. However, we treated the data as "mosaic diploids", a robust approach for phylogenetic inference in mixed-ploidy systems (the Methods, **Lines 716–719**).

• Some brief background on Poa sect. Stenopoa diversity in the introduction would help readers grasp the scope of the problem of Stenopoa. (Range of estimates of the number of Stenopoa species would also be useful. Brief discussion of species concepts?) Tzvelev & Probatova 2019 included 51 species for Russia! Liu 2003 ca 120 species for China.

Reply: We agree that providing context on the taxonomic complexity and diversity estimates for the section will help readers appreciate the scope of the challenges involved.

Thus, we have added a new paragraph to the Introduction (**Lines 92–103**) that:

(1) Outlines the profound disagreements in species delimitation within the section and presents the wide range of diversity estimates.
(2) Briefly discusses the underlying reasons for this instability, including apomixis, polyploidy, hybridisation, and shifts in breeding systems; and notes the difficulties of applying the biological species concept to these lineages, which often form continua of microspecies.

• Cluster I is only Poa palustris?. More discussion of the selection / identification of "genetically pure individuals" and exclusion of other samples or taxa would be helpful. e.g. What happened to Poa nemoralis? Poa nemoralis is another mesomorphic species that seems to share the identical TLF plastid sequences in multiple samples (5 each), differing from most other Stenopoas a bit, while their nrDNA profiles are diverse and do not unite these two taxa. (24 Stenopoa taxa sampled for plastid sequences by Gillespie and Soreng pers. comm here: more than 30 Stenopoa taxa have been sequenced for ITS nrDNA: many samples contributed by or co-collected by Olonova. You are welcome to cite this pers. comm. if you like and feel it is appropriate).

The other two clusters, II & III, correspond to sets of species.

Reply: Cluster I is not limited to *P. palustris*. As detailed in Supplementary Table 1, this genetic cluster also contains distinct individuals morphologically identified as *P. nemoralis* (ID2145), *P. sichotensis*, *P. intricata*, and *P. figertii*. The representation in Figure 3 reflects our rigorous two-step filtering strategy aimed at defining robust units for the SNAPP time-tree and morphological analysis: (1) first, we identified broad genetic clusters (I, II, III) using STRUCTURE (K = 3; Main text, Fig. 1). At this stage, *P. nemoralis* (ID 2145) was indeed assigned to Cluster I with high probability ($Q \geq 0.99$); (2) second, we performed a fine-scale investigation of genomic structure within each cluster (Supplementary Figure 1) to identify cohesive genetic groups (sub-clusters). The goal was to retain only "pure" units to avoid the "blurring" of morphological traits caused by transgressive segregation or introgression. We have modified our Methods (**Lines 753–759**) to explicitly explain this strategy.

Additionally, we appreciate the reviewer sharing the personal communication regarding plastid vs. nrDNA conflicts in *P. nemoralis*. Our nuclear DArTseq results align with this complexity. While one *P. nemoralis* individual grouped firmly within Cluster I, many others were identified as admixed (e.g., between Cluster I and III). This supports the reviewer's observation that while plastid markers might unite them, nuclear genomic data reveal a more reticulate history. Further taxonomic resolution (e.g., within *P. nemoralis* specifically) would likely require a higher density of SNPs to fine-tune delimitation and test for introgression at the genome level.

• To this reviewer, the separation of Poa palustris (cluster I) from the other taxa seems to present a rather circular argument that the mesomorphic form corresponds to the mesomorphic habitat.

Reply: We assure the reviewer that the definition of Cluster I was not circular. Our primary clustering analyses (STRUCTURE, PCA, and Neighbor-Net) were performed exclusively using genomic data (SNPs), without any input regarding morphology, taxonomy, or habitat. The fact that this genetically defined cluster corresponds so strongly to the mesomorphic phenotype and mesic habitat is, therefore, a biological result, not an artifact. It indicates that genetic divergence in this section is tightly coupled with ecological adaptation and morphological differentiation. The separation of *P. palustris* (and related taxa in Cluster I) is driven by their distinct genomic profile, which happens to map onto the mesomorphic "adaptive peak". We have updated the Methods (**Lines 730–731**) to explicitly state that genetic clustering was performed blindly with respect to phenotype to avoid any confusion regarding circularity.

• The vouchering is well done in Suppl. Table 1. Are all the sample vouchers at TK? The distribution of the samples/taxa in clusters I, II, III or overlapping/admixture is made clear in Suppl. Table I. This could also be done in Suppl. Table 9, where mismatches between DNA and morphology are noted among all samples. Some relevant taxonomic implications for Stenopoa taxa would be of interest.

Reply: We confirm that all sample vouchers are deposited in the Herbarium of Tomsk State University (TK), as stated in the Materials and Methods section (**Lines 666–667**).

Furthermore, we have updated Supplementary Table 9 to explicitly include the genetic cluster assignment (I, II, III, or Admixed) for each sample, making the mismatches between morphological identification and genetic background immediately apparent (similar to Supplementary Table 1).

Regarding the implications of these mismatches, our results suggest that phenotypic plasticity and reticulate evolution (hybridisation) have blurred the boundaries of traditional morphological species. For instance, the polyphyletic placement of *P. litvinoviana* (found in multiple unrelated genetic groups) implies that this taxon may represent a morphological form (ecotype) convergent across different lineages rather than a single evolutionary unit. Similarly, the grouping of *P. nemoralis* and *P. intricata* within the *P. palustris*-dominated Cluster I suggests these taxa share a cohesive genomic background despite subtle morphological differences. We have added a brief discussion of these specific taxonomic implications to the Discussion (**Lines 617–624**), noting that while our data exposes these inconsistencies, a formal taxonomic revision would require further cytogenetic work and analysis of type specimens (**Lines 637–639**).

• Poa x figertii (compressa x nemoralis) and Poa x intricata (nemoralis x palustris) present in cluster I were published as hybrids. Some comments on their Pure versus Admixture characterizations, and DNA mismatches would be interesting. These could be marked as hybrid formulae in Suppl. Table 1.

Reply: We appreciate the reviewer's detailed knowledge of these taxa. Regarding their characterisation as "pure" versus "admixed" in our molecular analysis, we observe a complex pattern that highlights the difficulty of applying these discrete names to dynamic genomic entities.

These taxa do not form uniform genetic groups in our dataset. For example, among individuals morphologically identified as *P. x figertii*, only two (ID15955, ID2203) clustered within "pure"

Cluster I, while the other four were admixed between Clusters I and III. Similarly, *P. x intricata* showed a split distribution: 12 individuals fell within "pure" Cluster I, while 10 were admixed.

Importantly, as the reviewer previously noted and our analysis confirmed, *P. nemoralis* itself is rarely "pure" in this dataset (only one individual, ID2145, fell into "pure" Cluster I, while most were admixed). This high variability suggests that specimens identified as *P. x figertii* or *P. x intricata* likely represent a continuum of introgression rather than discrete, stable F1 entities. The fact that the putative parent (*P. nemoralis*) is also largely admixed further blurs these boundaries.

Thus, we have addressed this by:

(1) Updating Supplementary Table 1 to include the putative hybrid formulae for *P. x figertii* and *P. x intricata* as suggested.
(2) Explicitly stating that this study was not designed for a comprehensive taxonomic revision in the Results (**Lines 303–305**) and addressing this limitation in the Discussion (**Lines 617–625**).

• After all this work it seems unfortunate to this reviewer that we don't have publicly deposited sequence data that can be applied to future studies. Can this study be built upon with more samples. Or would it need a whole redo?

Reply: We share the reviewer's commitment to open science and data reusability. To facilitate future studies, we have publicly deposited our SNP dataset (genlight format) on Figshare (see the Data Availability section, **Line 914**). Regarding the integration of new samples, the DArTseq platform allows for dataset expansion by co-analysing new samples against the existing reference catalogue of markers generated in this project. Future researchers can sequence new *Stenopoa* samples using the same protocol and request an analysis merged with our specific Project IDs (DPoa23-8026, DPoa23-7953, DPoa23-7951, and DPoa21-6177). This ensures that the exact same genomic loci are targeted and called without requiring a "whole redo". We have updated the Data Availability section to specify our Project IDs (**Lines 914–916**).

• Sequence data and analyses for Poa, including section Stenopoa species are available from quite a few more recent papers than cited in the text. This reviewer suggests expanding the references in this regard. Stenopoa taxa DNA sequences tend to show limited variation. This is also true of some other Poa clades including the very speciose (and the largest) clade of Poa in supersection Homalopoa in nrDNA and plastid analyses.

Cabi, E., R. J. Soreng & L. J. Gillespie. 2017. Taxonomy of Poa jubata and a new section of the genus (Poaceae). Turk. J. Bot. 41: 404–415, f. 1–4.
Cabi, E., R. J. Soreng, L. J. Gillespie & N. Amiri. 2016. Poa densa (Poaceae), an overlooked Turkish steppe grass, and the evolution of bulbs in Poa. Willdenowia 46(2): 201–211.
Gillespie, L. J., R. J. Soreng & S. W. L. Jacobs. 2009. Phylogenetic relationships of Australian Poa (Poaceae: Poinae), including molecular evidence for two new genera, Saxipoa and Sylvipoa. Austral. Syst. Bot. 22(6): 413–436, f. 1–3.
Gillespie, L. J., R. J. Soreng, E. Cabi & N. Amiri. 2018. Phylogeny and taxonomic synopsis of Poa subgenus Pseudopoa (including Eremopoa and Lindbergella) (Poaceae, Poeae, Poinae). PhytoKeys 111: 69–101.
Giussani, L. M., L. J. Gillespie, M. A. Scataglini, M. A. Negritto, A. M. Anton & R. J. Soreng. 2016. Breeding system diversification and evolution in American Poa supersect. Homalopoa (Poaceae: Poeae: Poinae). Ann. Bot. (Oxford) 118(2): 281–303.

Refulio-Rodríguez, N. F., J. T. Columbus, L. J. Gillespie, P. M. Peterson & R. J. Soreng. 2012. Molecular phylogeny of Dissanthelium (Poaceae: Pooideae) and its taxonomic implications. Syst. Bot. 37(1): 122–133.
Soreng, R. J. & L. J. Gillespie. 2018. Poa secunda J. Presl (Poaceae): a modern summary of infraspecific taxonomy, chromosome numbers, related species and infrageneric placement based on DNA. PhytoKeys 110: 101–121.
Soreng, R. J., L. J. Gillespie & L. L. Consaul. 2017. Taxonomy of the Poa laxa group, including two new taxa from Arctic Canada and Greenland, and Oregon, and a re-examination of P. sect. Oreinos (Poaceae). Nordic J. Bot. 35: 513–538.
Soreng, R. J., L. J. Gillespie, E. Boudko & E. Cabi. 2022. Biogeography, timing, and life-history traits in the PPAM clade: Coleanthinae (syn. Puccinelliinae), Poinae, Alopecurinae superclade, Miliinae, and Avenulinae and Phleinae (Poaceae, Pooideae, Poeae). J. Syst. Evol. 60(3): 591-620.
Soreng, R. J., L. J. Gillespie, H. Koba, E. Boudko & R. D. Bull. 2015. Molecular and morphological evidence for a new grass genus, Dupontiopsis (Poaceae tribe Poeae subtribe Poinae s.l.), endemic to alpine Japan, and implications for the reticulate origin of Dupontia and Arctophila within Poinae s.l. J. Syst. Evol. 53(2): 138–162.
Soreng, R. J., M. V. Olonova, N. S. Probatova & L. J. Gillespie. 2020. Breeding systems and phylogeny in Poa L., with special attention to northeast Asia: the problem of Poa shumushuensis Ohwi and sect. Nivicolae (Poaceae). J. Syst. Evol. 58(6): 1031–1058.
Soreng, R. J., R. D. Bull & L. J. Gillespie. 2010. Phylogeny and reticulation in Poa based on plastid trnTLF and nrITS sequences with attention to diploids. 619–644. In O. Seberg, G. Petersen, A. S. Barfod & J. I. Davis (eds.) Diversity, Phylogeny, Evol. Monocot. Aarhus University Press, Aarhus.
Sylvester, S. P., R. J. Soreng & L. J. Gillespie. 2021. Resolving páramo Poa (Poaceae): morphometric and phylogenetic analysis of the 'Cucullata complex' of north-west South America. Bot. J. Linn. Soc. 197: 104-146.

> **Reply:** We are very grateful for this comprehensive list of resources. While we could not incorporate every reference due to space constraints and our specific focus on the section *Stenopoa*, we have selected the most relevant papers (Soreng et al., 2010; Giussani et al., 2016; Soreng et al., 2020) to supplement our Introduction (**Lines 73**, **78–79** & **101**) and expand the Discussion (**Lines 544–548** and **561-562**).

• Also include:
For dating of grasses.
Gallaher, T. J., P. M. Peterson, R. J. Soreng, F. O. Zuloaga, D.-Z. Li, L. G. Clark, C. D. Tyrrell, C. A. D. Welker, E. A. Kellogg & J. K. Teisher. 2022. Grasses through space and time: An overview of the biogeographical and macroevolutionary history of Poaceae. J. Syst. Evol. 60(3): 522-569.

> **Reply:** We now cite this study in the Introduction (**Line 76**).

• And for Stenopoa diversity relating to the controversy over species delimitations.
In Russia
Tzvelev, N. N. & N. S. Probatova. 2019. Zlaki Rossii / Grasses of Russia. In N. N. Tzvelev & N. S. Probatova Zlaki Rossii / Grasses of Russia. KMK Scientific Press, Moscow.
Tzvelev, N. N. 2009. On species of the genus Poa L. section Stenopoa Dumort. (Poaceae) in the eastern Europe. Novosti Sist. Vyssh. Rast. 41: 18–52.
Olonova, M. V. 1990 35 Poa L. Fl. Sibir. 2: 165–189
Olonova, M. V. 1998 The system and check-list of Siberian bluegrasses (Poa L.) Turczaninowia 1(4): 5–19

Olonova, M. V. 2007 Variability of bluegrass (Poa) in Siberia Aliso 23: 435–449
Olonova, M. V. 2008 Towards the study of bluegrass complex Poa versicolor Bess. (Poaceae) in northern China Animadvers. Syst. Herb. Univ. Tomsk. 99: 16–24
Olonova, M. V. 2010 The synopsis of section Stenopoa of Poa (Poaceae) in Siberia Bot. Zhurn. (Moscow & Leningrad) 95(6): 869–881
Olonova, M. V. 2016 Synopsis of blugrasses (Poa L., Poaceae) of the Altai mountain system Sist. Zametki Mater. Gerb. Krylova Tomsk. Gosud. Univ. Kuybysheva 113: 68–88
Olonova, M. V. & E. G. Nikolin 2018 Bluegrasses (Poa L., Poaceae) of Sakha (Yakutia) Republic, an annotated checklist Adansonia, sér. 3, 40(1): 89–102
Olonova, M. V. & S. P. Sylvester 2021 Poa liuliangii and P. kengii (Poaceae), two new species from the Hengduan Mountains of southwest China Taiwania 66(2): 251-257
Olonova, M. V. & V. V. Chepinoga 2022 Poa probatovae (Poaceae), a new species from the Eastern Siberia Turczaninowia 25(1): 140-145
Olonova, M. V. & W.-L. Chen 2010 Systematic revision of the Poa sphondylodes Trin. complex (Poaceae) Candollea 65(1): 135–141
Olonova, M. V., M.E. Barkworth, S. V. Pulkina & W.-L. Chen 2011 Morphological variation in populations of the Poa orinosa complex (Poaceae: Poeae) in northeastern China Nordic J. Bot. 29: 762–772
Olonova, M. V., V. D. Shiposha & R. S. Romanets 2022 Phenotypic plasticity of the stem epidermis in the bluegrasses (Poa L.) of section Stenopoa Dumort. (Poaceae). I. Mesomorphic species Acta Biol. Sibirica 8: 655-672
The Himalaya front ranges
Rajbhandari, K. R. 1991. A revision of the genus Poa L. (Gramineae) in the Himalaya. Bull. Univ. Mus. Univ. Tokyo 34: 169-263, f. 1-29 + maps f. 30-43.
In China
Liu, L. 2003. Poa. Fl. Reipubl. Popularis Sin. 9(2): 91–226.

> **Reply:** We are aware of the controversy over species delimitations in *Stenopoa*. Following the recommendation above, we now added a new paragraph (**Lines 92–103**). Nonetheless, as we tried to avoid focusing on species decimation as it narrows down the study to only specialists in the field and requires a separate article, we limit citing taxonomical articles to minimum as possible.

• Liu 2003 listed 246 species of Poa from China, as this reviewer recalls about 120 were attributable to Stenopoa species. Whereas Olonova (in Zhu et al. 2006) only accepted 15 species but many infraspecies. (below -- ! = ok name when homonyms exist, * = illegitimate names, ** = invalid names.)
Poaceae ! Poa botryoides (Trin. ex Griseb.) Kom.
Poaceae Poa chaixii Vill.
Poaceae Poa compressa L.
Poaceae Poa khasiana Stapf
Poaceae Poa malacantha Kom.
Poaceae Poa sichotensis Prob.
Poaceae Poa stepposa (Krylov) Roshev.
Poaceae Poa urssulensis Trin.
Poaceae Poa almasovii Golub
Poaceae Poa altaica Trin.
Poaceae ! Poa arctica R. Br.
Poaceae ! Poa attenuata Trin.

Poaceae Poa badensis Haenke ex Willd.
Poaceae Poa bulbosa subsp. vivipara (Koeler) Arcang.
Poaceae Poa densa Troitsky
Poaceae ! Poa hybrida Gaudin
Poaceae Poa insignis Litv.
Poaceae Poa paucispicula Scribn. & Merr.
Poaceae Poa platyantha Kom.
Poaceae ! Poa pumila Host
Poaceae Poa shumushuensis Ohwi
Poaceae ! Poa sibirica Roshev.
Poaceae ! Poa sylvicola Guss.
Poaceae Poa tolmatchewii Roshev.
Poaceae Poa transbaicalica Roshev.
Poaceae Poa vrangelica Tzvelev
Poaceae Poa ampla Merr.
Poaceae Poa annua var. reptans Hausskn.
Poaceae Poa eminens J. Presl
Poaceae Poa irkutica Roshev.
Poaceae Poa smirnowii Roshev.
Poaceae ! Poa acroleuca Steud.
Poaceae Poa hisauchii Honda
Poaceae Poa nipponica Koidz.
Poaceae Poa radula Franch. & Sav.
Poaceae Poa hayachinensis Koidz.
Poaceae Poa sachalinensis (Koidz.) Honda
Poaceae Poa sphondylodes Trin.
Poaceae Poa viridula Palib.
Poaceae Poa lanata Scribn. & Merr.
Poaceae ! Poa laxa Haenke
Poaceae Poa tibeticola Bor
Poaceae ! Poa remota Forselles
Poaceae ! Poa albertii Regel
Poaceae ! Poa angustiglumis Roshev.
Poaceae Poa bracteosa Kom.
Poaceae Poa bucharica Roshev.
Poaceae Poa bulbosa subsp. nevskii (Roshev. ex Ovcz.) Tzvelev
Poaceae Poa ciliatiflora Roshev.
Poaceae Poa dahurica Trin.
Poaceae Poa densissima Roshev. ex Ovcz.
Poaceae Poa dschungarica Roshev.
Poaceae ! Poa hissarica Roshev. ex Ovcz.
Poaceae * Poa irrigata Lindm.
Poaceae ! Poa karateginensis Roshev. ex Ovcz.
Poaceae Poa kolymensis Tzvelev
Poaceae Poa komarovii Roshev.
Poaceae Poa korshunensis Golosk.
Poaceae Poa krylovii Reverd.
Poaceae ! Poa laudanensis Roshev. ex Ovcz.

Poaceae Poa lipskyi Roshev.
Poaceae Poa lipskyi subsp. dschungarica (Roshev.) Tzvelev
Poaceae ! Poa longifolia Trin.
Poaceae Poa meyeri Trin. ex Roshev.
Poaceae ! Poa nevskii Roshev. ex Ovcz.
Poaceae Poa ochotensis Trin.
Poaceae ! Poa pamirica Roshev. ex Ovcz.
Poaceae Poa phariana Bor
Poaceae ! Poa pruinosa Korotky
Poaceae Poa raduliformis Prob.
Poaceae Poa rangkulensis Ovcz. & Czukav.
Poaceae Poa relaxa Ovcz.
Poaceae ! Poa rhomboidea Roshev.
Poaceae Poa sabulosa (Turcz. ex Roshev.) Turcz. ex Roshev.
Poaceae ! Poa schischkinii Tzvelev
Poaceae Poa sibirica subsp. uralensis Tzvelev
Poaceae Poa subfastigiata Trin.
Poaceae Poa tianschanica (Regel) Hack. ex O. Fedtsch.
Poaceae Poa tristis Trin. ex Regel
Poaceae Poa trivialiformis Kom.
Poaceae Poa versicolor Besser
Poaceae Poa versicolor subsp. relaxa (Ovcz.) Tzvelev
Poaceae Poa versicolor subsp. stepposa (Krylov) Tzvelev
Poaceae Poa vvedenskyi Drobow
Poaceae Poa zaprjagajevii Ovcz.
Poaceae ! Poa trichophylla Heldr. & Sartori ex Boiss.
Poaceae ! Poa ianthina Keng ex Shan Chen
Poaceae ! Poa pachyantha Keng ex S.L. Chen
Poaceae ! Poa patens Keng ex Keng f.
Poaceae ! Poa paucifolia Keng ex Shan Chen
Poaceae ! Poa perennis Keng ex Keng f.
Poaceae ** Poa plurinodis Keng ex Keng f.
Poaceae Poa mairei Hack.
Poaceae Poa arnoldii Melderis
Poaceae Poa bomiensis C. Ling
Poaceae Poa borealitibetica C. Ling
Poaceae ! Poa crymophila Keng ex C. Ling
Poaceae * Poa digena Melderis
Poaceae Poa langtangensis Melderis
Poaceae ! Poa macrolepis Keng ex C. Ling
Poaceae ! Poa malaca Keng
Poaceae ! Poa micrandra Keng
Poaceae Poa nigropurpurea C. Ling
Poaceae Poa nimuana C. Ling
Poaceae ! Poa oligophylla Keng
Poaceae ! Poa orinosa Keng
Poaceae ! Poa tunicata Keng ex C. Ling
Poaceae ! Poa vaginans Keng

Poaceae Poa zhongbaensis C. Ling
Poaceae ! Poa megalothyrsa Keng ex Tzvelev
Poaceae Poa roemeri Bor
Poaceae Poa lhasaensis Bor
Poaceae Poa afghanica Bor
Poaceae Poa alta Hitchc.
Poaceae Poa arjinsanensis D.F. Cui
Poaceae Poa florida N.R. Cui
Poaceae Poa macroanthera D.F. Cui
Poaceae Poa major D.F. Cui
Poaceae Poa membranigluma D.F. Cui
Poaceae Poa nemoralis var. parca N.R. Cui
Poaceae Poa parvissima Cui ex D. F. Cui
Poaceae ! Poa poophagorum Bor
Poaceae * Poa scabristemmed N.R. Cui
Poaceae Poa kelungensis Ohwi
Poaceae Poa imperialis Bor
Poaceae ** Poa mongolica (Rendle) Keng
Poaceae Poa asperifolia Bor
Poaceae Poa himalayana Nees
Poaceae Poa polyneuron Bor
Poaceae Poa spontanea Bor
Poaceae Poa burmanica Bor
Poaceae Poa eleanorae Bor
Poaceae Poa gamblei Bor
Poaceae Poa jaunsarensis Bor
Poaceae Poa kanboensis Ohwi
Poaceae Poa nephelophila Bor
Poaceae Poa nitidespiculata Bor
Poaceae Poa rhadina Bor
Poaceae Poa sinoglauca Ohwi
Poaceae Poa wardiana Bor
Poaceae Poa grandis Hand.-Mazz.
Poaceae Poa rossbergiana K.S. Hao
Poaceae Poa taiwanicola Ohwi
Poaceae Poa debilior Hitchc.
Poaceae ! Poa argunensis Roshev.
Poaceae ! Poa dshilgensis Roshev.
Poaceae Poa formosae Ohwi
Poaceae Poa nankoensis Ohwi
Poaceae ! Poa reverdattoi Roshev.
Poaceae Poa takasagomontana Ohwi
Poaceae Poa tenuicula Ohwi
Poaceae Poa diversifolia (Boiss. & Balansa) Hack. ex Boiss.
Poaceae Poa hirtiglumis Hook. f.
Poaceae Poa prolixior Rendle
Poaceae Poa shansiensis Hitchc.
Poaceae Poa tangii Hitchc.

Poaceae Poa faberi Rendle
Poaceae Poa pilipes Keng ex Shan Chen
Poaceae Poa granitica Braun-Blanq.
Poaceae Poa acmocalyx Keng ex L. Liu
Poaceae Poa binodis Keng ex L. Liu
Poaceae ! Poa breviligula Keng ex L. Liu
Poaceae Poa chalarantha Keng ex L. Liu
Poaceae ! Poa declinata Keng ex L. Liu
Poaceae * Poa elanata Keng ex L. Liu
Poaceae Poa eragrostioides L. Liu
Poaceae Poa fascinata Keng ex L. Liu
Poaceae Poa flavida Keng ex L. Liu
Poaceae Poa gracilior Keng ex L. Liu
Poaceae Poa grandispica Keng ex L. Liu
Poaceae ! Poa incerta Keng ex L. Liu
Poaceae Poa hengshanica Keng ex L. Liu
Poaceae Poa lepta Keng ex L. Liu
Poaceae ** Poa levipes (Keng) Keng ex L. Liu
Poaceae Poa lithophila Keng ex L. Liu
Poaceae Poa maerkangica L. Liu
Poaceae ! Poa nubigena Keng ex L. Liu
Poaceae Poa pubicalyx Keng ex L. Liu
Poaceae Poa schoenites Keng ex L. Liu
Poaceae Poa tetrantha Keng ex L. Liu
Poaceae Poa stereophylla Keng ex L. Liu
Poaceae Poa varia Keng ex L. Liu
Poaceae Poa yakiangensis L. Liu
Poaceae Poa zhongdianensis L. Liu
Poaceae Poa pratensis var. anceps (Gaudin) Griseb.
Poaceae Poa platyglumis (L. Liu) L. Liu
Poaceae Poa nemoralis subsp. nemoralis
Poaceae Poa bulbosa var. bulbosa
Poaceae * Poa media Schur
Poaceae Poa annua var. annua
Poaceae Poa pseudamoena Bor
Poaceae ! Poa alpigena Lindm.
Poaceae Poa skvortzovii Prob.
Poaceae ** Poa pseudopalustris Keng
Poaceae Poa ludens R.R. Stewart
Poaceae Poa szechuensis Rendle
Poaceae Poa ligulata Boiss.
Poaceae ** Poa spiciformis D.F. Cui
Poaceae ** Poa dolichachyra Keng ex L. Liu
Poaceae * Poa indattenuata Keng ex L. Liu
Poaceae * Poa pseudopalustris Keng ex L. Liu
Poaceae * Poa psilolepis Keng ex L. Liu
Poaceae * Poa sinattenuata Keng ex L. Liu
Poaceae ** Poa stenachyra Keng ex L. Liu

Poaceae Poa parafestuca L. Liu
Poaceae Poa timoleontis Heldr. ex Boiss.
Poaceae Poa masenderana Freyn & Sint.
Poaceae ! Poa pratensis subsp. alpigena (Lindm.) Hiitonen
Poaceae ! Poa abbreviata R. Br.
Poaceae Poa sikkimensis (Stapf) Bor
Poaceae Poa supina Schrad.
Poaceae Poa stewartiana Bor
Poaceae Poa bactriana subsp. glabriflora (Roshev.) Tzvelev
Poaceae Poa sinaica Steud.
Poaceae ! Poa glabriflora (Roshev.) Roshev. ex Ovcz.
Poaceae Poa koelzii Bor
Poaceae ! Poa nepalensis (Wall. ex Griseb.) Duthie
Poaceae ! Poa lahulensis Bor
Poaceae ! Poa pagophila Bor
Poaceae Poa setulosa Bor
Poaceae Poa stapfiana Bor
Poaceae ! Poa litvinoviana Ovcz.
Poaceae ! Poa alpina L.
Poaceae ! Poa angustifolia L.
Poaceae ! Poa nemoralis L.
Poaceae Poa araratica Trautv.
Poaceae Poa palustris L.
Poaceae Poa sterilis M. Bieb.
Poaceae Poa infirma Kunth
Poaceae Poa falconeri Hook. f.
Poaceae Poa aitchisonii Boiss.
Poaceae Poa bactriana Roshev.
Poaceae ! Poa calliopsis Litv. ex Ovcz.
Poaceae Poa tibetica Munro ex Stapf
Poaceae Poa polycolea Stapf
Poaceae Poa arctica subsp. caespitans Simmons ex Nannf.
Poaceae Poa macrocalyx Trautv. & C.A. Mey.
Poaceae ! Poa glauca Vahl
Poaceae !! Poa pratensis L.
Poaceae ! Poa annua L.
Poaceae Poa bulbosa var. vivipara Koeler
Poaceae ! Poa bulbosa L.
Poaceae Poa trivialis L.
Poaceae Poa triglumis Keng f. ex L. Liu
Poaceae ! Poa plurifolia Keng
Poaceae Poa longiglumis Keng ex L. Liu

Reply: We are grateful for providing this detailed compilation of nomenclature and the comparison between the treatments of Liu (2003) and Olonova (Zhu et al., 2006). This contrast, where one treatment accepts ~120 *Stenopoa* species and another only 15, perfectly illustrates the magnitude of the taxonomic challenge in this group. We have updated the Introduction (**Lines 92–103**) to explicitly mention this discrepancy. We now cite these conflicting estimates

to highlight why traditional morphological taxonomy has struggled to resolve the section and why a genomic approach is necessary. We have also ensured that the relevant references (Liu, 2003; Zhu et al., 2006; Tzvelev & Probatova, 2019) are included in the References.

##

Referee #3: Plant evolution and population genomics

Reviewer #3 (Remarks to the Author):

##

The present manuscript is an ambitious and integrative study that combines different approaches from genomics to morphology and paleoclimate niche construction analysis to understand diversification dynamics in one of interesting yet taxonomically very complex plant lineages characterized by whole genome duplication. I am impressed by how the authors rigorously conducted the study and incorporated sound analytical approaches to investigate the evolutionary dynamics of a non-model polyploid taxa and improve our understanding of Pleistocene diversification in grass. However, I have some critical methodological issues that, if addressed, may improve the current version of the manuscript:

• Line 483: As already mentioned in the paper, polyploidy is the central focus of the study system and the key driver of diversification and adaptation. However, it is surprising not to see even a single attempt to do a direct chromosome count or ploidy estimation in the present paper. Instead, the authors decided to rely on previously published and incomplete chromosome number data and state that chromosome counting is beyond the scope of the study. Given that the present paper is framed around polyploidy, this statement is completely unacceptable and potentially makes any of the biological interpretations made in the study questionable as one cannot be sure if any of the inferred groups reflect true evolutionary lineage or are influenced by cytotype variation.

Reply: We appreciate the reviewer's critical insight regarding the framing of polyploidy in this study. We fully agree that polyploidy is a central feature of *Poa* sect. *Stenopoa*. However, obtaining new, direct ploidy data for the individuals in this study was unfeasible for several reasons:

(1) The primary constraint was our reliance on historical herbarium material, collected across a vast geographic range over a 47-year period (1975–2022). We attempted flow cytometry on a subset of these samples, but nuclei degradation in the dried tissue compromised the data, preventing ploidy estimation.
(2) Generating living material from these vouchers is practically impossible. Due to the extremely short vegetation season in the alpine zone, specimens are almost exclusively collected during the flowering stage to ensure taxonomic identification; consequently, seeds on the vouchers are typically immature. Furthermore, even in rare cases where seeds might be present, these species lose viability rapidly in herbarium storage, making successful germination rates negligible. Additionally, the cytology of this section (with chromosome numbers ranging from 2n=14 to 2n=ca.100) is technically demanding and necessitates a separate, methodologically focused investigation.
(3) We consulted with our genotyping provider (Diversity Arrays Technology) regarding *in silico* ploidy estimation based on read depth (dose calling). They confirmed that standard DArTseq read depths (<30x for most markers) are insufficient for reliable dose calling in high-ploidy species. In practice, this method requires both a minimum depth of >60x and reference samples of known ploidy (e.g., verified by flow cytometry) for each expected ploidy level to calibrate the analysis. This makes the approach particularly challenging for our system, where ploidy varies widely and such a comprehensively validated reference set is unavailable. **Importantly, the**

genome complexity reduction inherent to DArTseq effectively treats high-ploidy samples as "mosaic diploids" during marker calling. Therefore, our final biological conclusions based on this "mosaic" data are robust and would not differ from an analysis that explicitly accounted for ploidy. For instance, recent research by Wróbel et al. (2024) successfully applied the DArTseq platform in the mixed-ploidy non-model system *Puccinellia*.

To conclude, while we could not generate new cytological data, we have addressed the reviewer's concern that the paper is "framed around polyploidy" without sufficient data by revising the manuscript structure:

- We have modified the Abstract (**Lines 34–35**) to present polyploidy as the evolutionary context of the lineage rather than the primary variable being tested.
- We have added a specific aim: "synthesising available cytological data to contextualise our findings" (**Lines 122–123**).
- We have added a new section, "Cytological variation" / "Cytological data compilation", explicitly explaining why we relied on a literature review rather than direct estimation (**Lines 462–472 & 904–909**).
- We have emphasised that the identified clusters likely represent evolutionary lineages rather than simple cytotypes, supported by our literature review (Supplementary Table 15) which shows that multiple ploidy levels exist within the same taxonomic and genetic groups (the Discussion, **Lines 531–537**).

Reference

Wróbel, A., Klichowska, E. & Nobis, M. Hybrids as mirrors of the past: genomic footprints reveal spatio-temporal dynamics and extinction risk of alpine extremophytes in the mountains of Central Asia. *Frontiers in Plant Science* **15**, 1369732 (2024).
https://doi.org/10.3389/fpls.2024.1369732

• Line649 & Line126: In the method section (line 649) the authors describe how the optimal K was determined objectively; the way the results are presented (line 126) appears more subjective as the authors claim that the data "can be divided into two or three "clusters. This way of presentation is not acceptable. The authors should clearly describe how they objectively determined the optimal K by reporting and describing the result from the Evannon deltaK method and whether the chains converged across the different runs, including an assessment of cluster stability across replicated runs.

Reply: We apologise for the lack of explicit detail in the original manuscript text in the Results regarding the objective determination of the optimal *K*. We confirm that the Evanno method (delta *K*) was used to determine the most likely number of genetic clusters (*K*). As shown in Supplementary Data 1 (folder STRUCTURE; and below in Fig. 2), the analysis revealed a clear primary peak at K = 2 (delta K > 6,000), with a secondary peak at K = 3 (delta $K \approx$ 2,500). While K = 2 represented the statistical optimum, we proceeded with the K = 3 scenario as it better resolves the biologically and geographically distinct Tibetan Plateau lineage as a separate cluster (Cluster II), which was subsumed within the broader groupings at K = 2. We have revised **Line 148** in the Results to add these delta *K* values.

Regarding technical robustness, we have updated the Methods (**Lines 737–742** & **Lines 744–746**) to explicitly describe our validation steps:

(1) All MCMC chains showed strong convergence (example diagnostics for $K = 2$ and $K = 3$ are reproduced in Figs. 3, 4 below; interactive diagnostics in html format available via Figshare, **Line 914**, in Supplementary Data 1, folder STRUCTURE).

(2) We assessed cluster stability by comparing the 10 independent replicate runs for each K using pophelper. This confirmed that individual assignments were consistent across all replicates, as shown in Fig. 5 below.

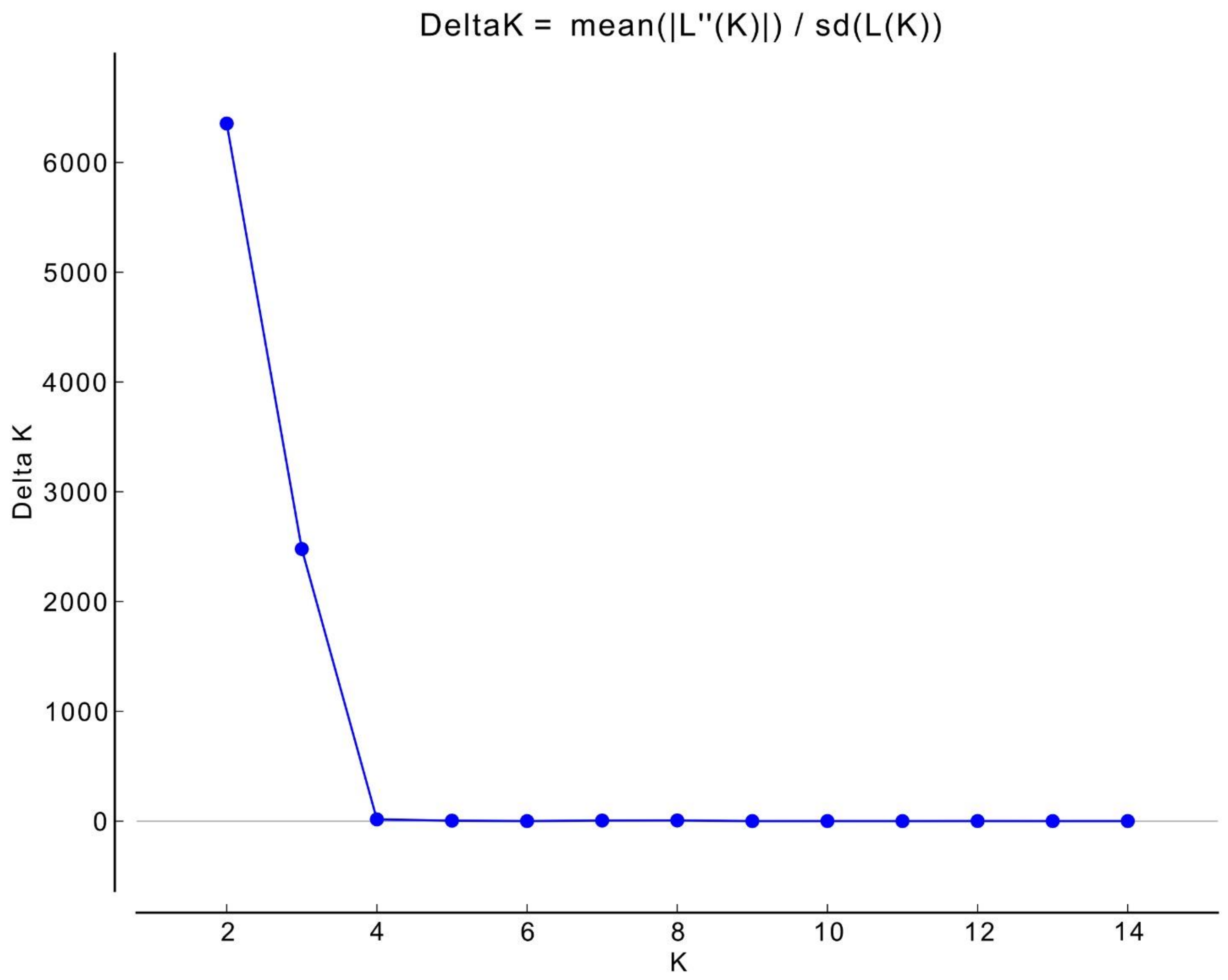

Figure 2. Evanno's Delta K values for the STRUCTURE analysis presented in the main text. The plot shows a primary peak at $K = 2$ (Delta K > 6,000).

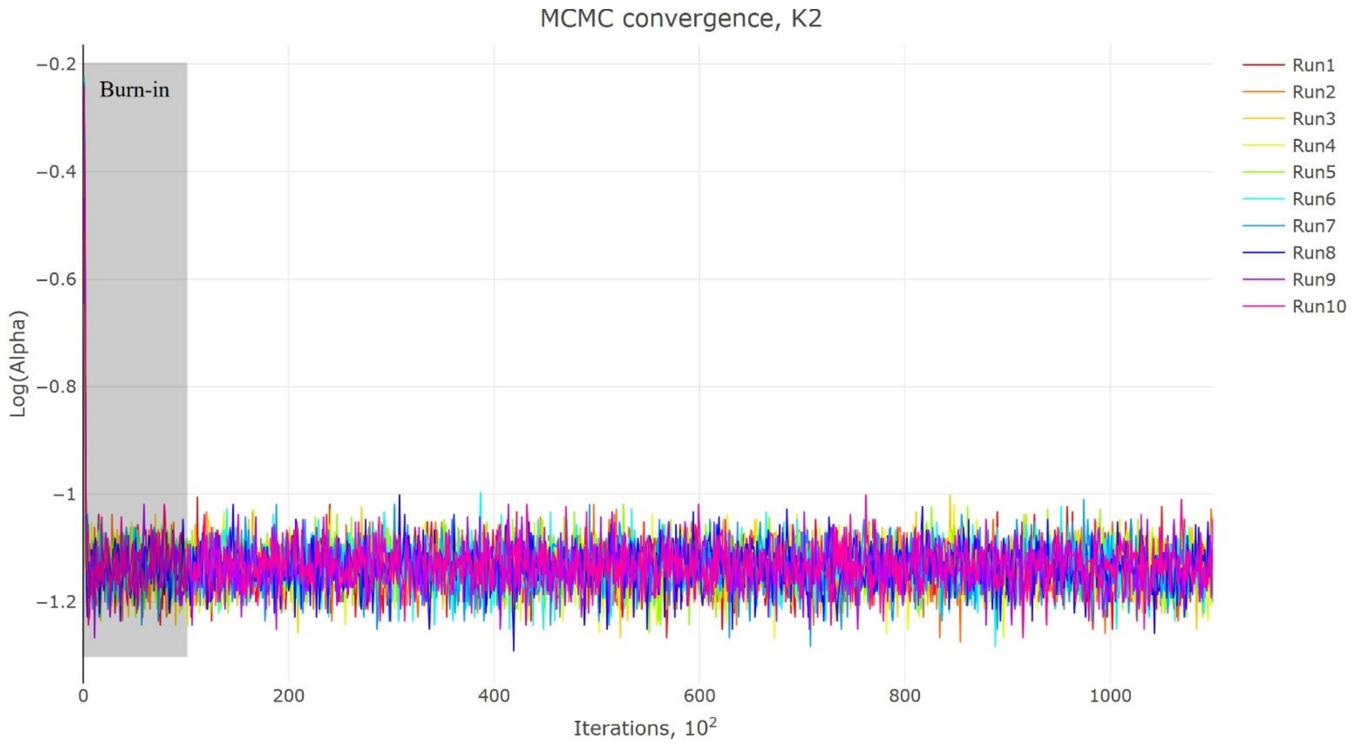

Figure 3. MCMC convergence diagnostics for STRUCTURE at $K = 2$. The X-axis represents the number of MCMC iterations, while the Y-axis represents the log-transformed Alpha (α) parameter. Visual inspection of the ten independent runs (coloured lines) confirms that the chains reached a stable plateau/convergence.

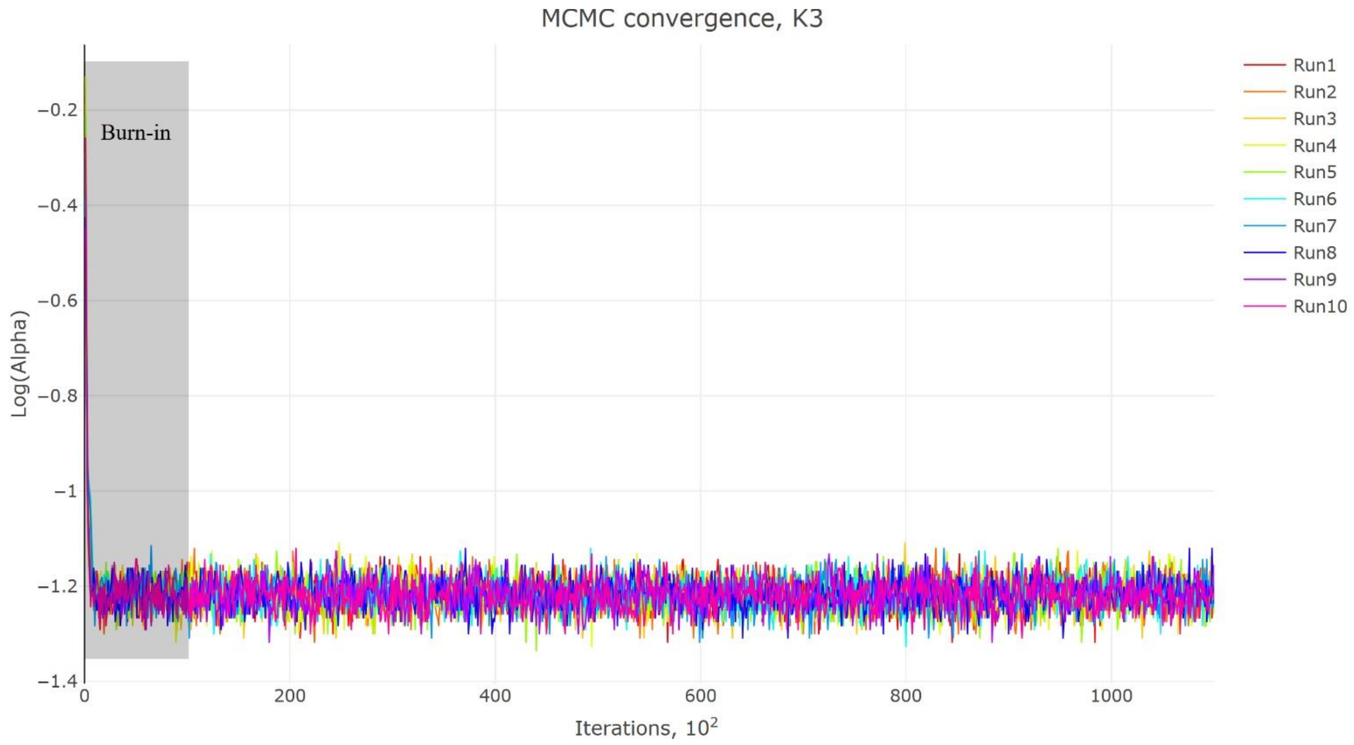

Figure 4. MCMC convergence diagnostics for STRUCTURE at $K = 3$. The X-axis represents the number of MCMC iterations, while the Y-axis represents the log-transformed Alpha (α) parameter. Visual inspection of the ten independent runs (coloured lines) confirms that the chains reached a stable plateau/convergence.

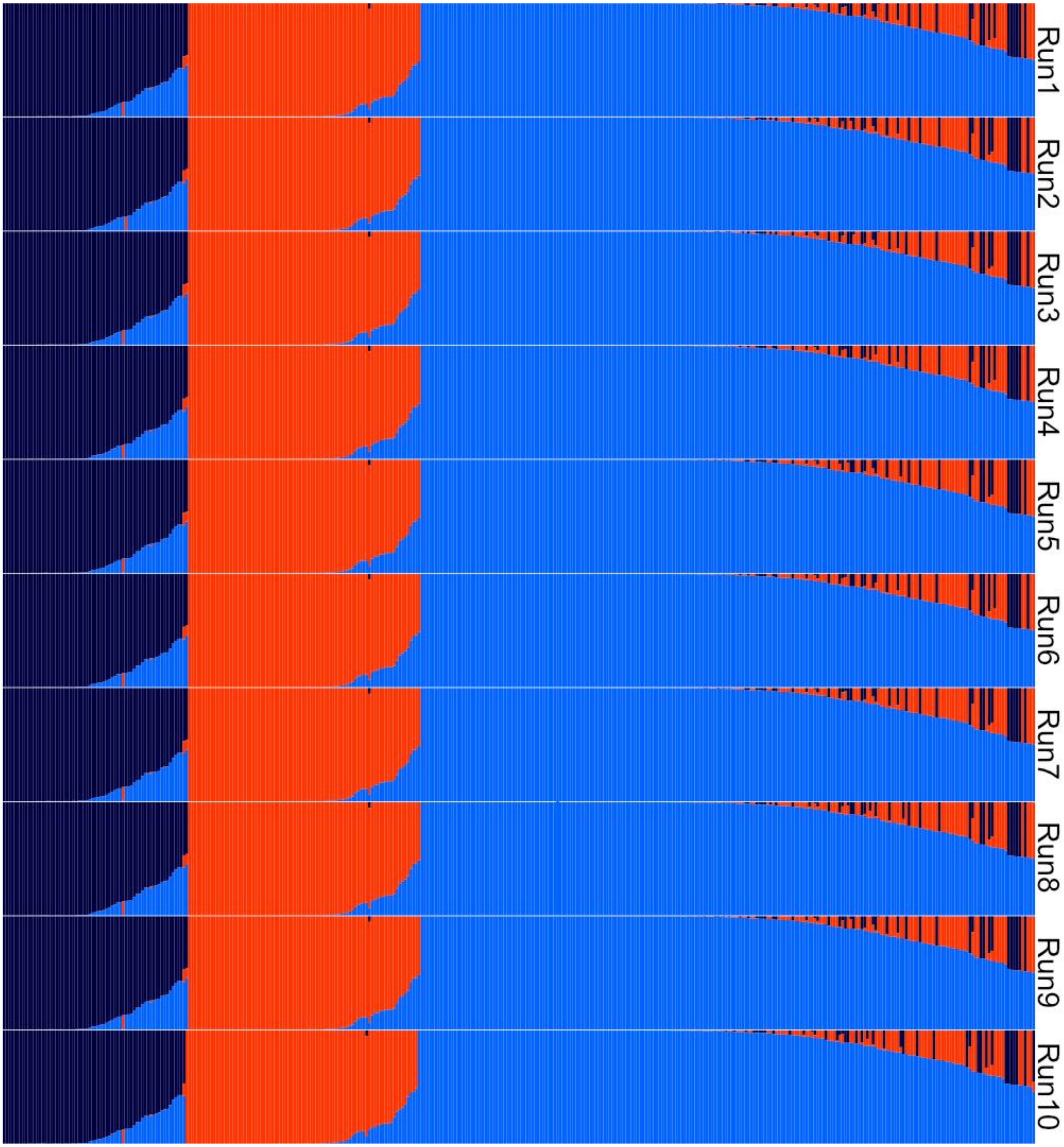

Figure 5. Assessment of cluster stability by comparing 10 independent replicate runs for *K* = 3 (n = 373) using pophelperShiny v.2.1.1. The plot demonstrates consistent individual assignment probabilities across all runs (ordered and aligned within *K*), confirming high stability and the absence of multimodality.

• The use of reduced-representation snp data (DarTseq) is well suited for inferring genetic structuring and recent divergence but to robust testing of fine-scale demographic inference may be challenging, particularly in plant lineages characterized by extensive polyploidy, hybridization, and reticulation. Furthermore, some of the analytical methods assume diploid and unlinked loci. I would therefore expect the authors to justify, in the method section, the suitability of these methods for polyploidy taxa or alternatively to tone down the interpretation of the results and discuss them with some curiosity.

Reply: We agree that demographic inference with reduced-representation data in complex polyploid systems requires careful justification and cautious interpretation. To address the specific concerns raised:

(1) As detailed in our previous response, we treat the DArTseq data from these high-ploidy individuals as a "mosaic diploid" during bioinformatic processing. This is a standard approach for such datasets and mitigates the challenge of undefined ploidy levels. We have added a clear justification for this strategy in the Methods (**Lines 716–719**).
(2) To meet the assumption of unlinked loci, our analysis retained only one random SNP per sequence tag (DArT locus), effectively filtering out secondary, linked SNPs (**Lines 724–726**).

Regarding the interpretation of results, we fully agree with the reviewer that results from such analyses should be presented with appropriate caution. We have updated the Discussion (**Lines 627–632**) highlighting the limitation posed by the relatively low number of SNPs compared to whole-genome studies and advised treating demographic estimates with caution.
##

We would like to thank the reviewers for their time and for their detailed and constructive comments, which have significantly improved our manuscript. For your convenience, the original comments are presented in bold type, and each of our responses is indented below the corresponding comment.

##

Reviewers' comments:

##

Referee #1: Plant systematics and ecology

Reviewer #1 (Remarks to the Author):

##

I am reviewing the manuscript entitled “Pleistocene adaptation and diversification in cold, arid environments: genomic and paleoclimate insights from non-model bluegrasses" by Dr Baiakhmetov and colleagues. This is my second time reviewing (Reviewer #1 in the previous revision).

First of all, I appreciate the work the authors did, especially with presenting polyploidy and cytological variation as a background evolutionary context and not a tested variable. I also like that the authors provided more details about morphological and environmental traits and presented them in a wider context. The added paragraph about taxonomic complexity now provides more context for their discussion while not dwelling on too many taxonomic details and complexity, which seems to be one of the next steps in understanding Stenopoa evolution.

- **Despite the great improvement of the already very nice manuscript, the study remains mostly Poa-oriented and descriptive. And, if the authors conclude that their study “provides a predictive framework for understanding how ongoing climate change might shape the future distribution and evolution of cold-adapted polyploid flora”, I would expect them to show such a prediction in their own work.**

  Reply: We appreciate this constructive challenge. We have extensively rewritten the Conclusion (**Lines 690–710**) to transform our framework into explicit, testable predictions. Specifically, we now project that continued aridification may favour the expansion of the xeromorphic Cluster III, while increasingly unpredictable monsoonal patterns will likely exacerbate the ongoing decline of the highly specialised Cluster II, trajectories consistent with extinction debts documented in other climate-threatened mountain floras (Dullinger et al., 2012; Rumpf et al., 2019; Wróbel et al., 2024). We also explicitly acknowledge the assumptions underlying these predictions (e.g., niche stability) and outline experimental approaches needed to test them. We believe this revision significantly elevates the paper's final takeaway, and we thank the reviewer for pushing us to make these predictions explicit.

I still have several comments which the authors may find useful in helping the manuscript clarity and coherence, and some additional questions about the work and its results.

- **Line 45: Please rephrase the sentence to accommodate allopolyploidy not being the only mechanism of polyploid formation. I suggest something along the lines "Among the mechanisms of polyploid formation, WGD may follow after hybridisation (allopolyploidy)."**

  In addition, polyploidy is not driven by hybridisation; it is a mechanism that sometimes follows hybridisation and enables plants to deal with two distinct chromosome sets. Not all hybrids are allopolyploids. Importantly, the fertility restoration due to polyploidy does not always happen (e.g. not all allopolyploids are fertile), so the statement should be softened (e.g., by adding may). Plus, a reference example for fertility restoration should be provided.

  This first paragraph still uses WGD, genome doubling and polyploidy without really linking the terms together. I suggest adding polyploidy and genome doubling into the brackets in line 42: (WGD, aka polyploidy or genome doubling). While this is not absolutely accurate, it provides at least some connection between these terms.

  Reply: We have revised the first paragraph to explicitly link these terms upon first mention (**Line 42**). We also adopted your suggested phrasing to clarify that WGD is a mechanism that may follow hybridisation, rather than being driven by it. Finally, we softened the statement regarding fertility restoration (adding "may") and included a reference (Comai, 2005) to support this mechanism (**Lines 45–47**).

- **Lines 94 and 97: What species concepts are referred to here?**

  While the biological species concept is widely recognised, it is also widely acknowledged as unsuitable in most plant species because of the presence of, e.g. polyploidy or apomixis. Are there any other species concepts that would be more suitable for the study group? Would a new species concept help to solve the taxonomic complexity, as was done, e.g. in the case of Rubus? I do not dispute the fact that the group is likely a taxonomical nightmare, but I am inclined that it has a lot to do with the morphological variation, phenotypic plasticity and the lack of determining characters (plus polyploidy, apomixis, etc.), rather than the biological species concept only.

  Reply: Interesting point. In the text, we were primarily referring to the conflict between narrow (*sensu stricto*) and broad (*sensu lato*) morphological species concepts historically applied by different systematists. We have updated the text to make this explicit (**Lines 94–95**; **104–107**).

  Regarding your suggestion of proposing a new species concept (as was done in *Rubus*: the biological species concept for sexual taxa and an agamospecies concept for apomictic genotypes), while we agree that such an approach could be the ultimate goal, the current level of study in *Stenopoa* makes this premature. As our taxonomic expert (Prof. Olonova) notes, the operational viability of any species concept depends strictly on the level of available population data. In the poorly studied, hard-to-reach mountain territories of Asia, many *Stenopoa* species were described from single herbarium specimens without any data on population structure, reproductive mode, or modification-based variability. Under such conditions, taxonomists are historically forced to rely on a practical typological concept (assessing the degree of morphological difference from a holotype) simply to document and preserve a record of the existing diversity. Applying a stricter biological concept, or even a robust morphological concept, requires finding a clear biological or physical hiatus, which is impossible without knowing the full ecological and intraspecific variability of these populations.

Because of this, we believe that formally proposing a new, rigid species concept right now would be counterproductive. Based on our taxonomic expertise, we propose that a more pragmatic current solution is to treat these unclear, minor morphotypes within informal "aggregates" of established species. Grouping them into informal aggregates, rather than formally reducing them to subspecies within a polytypic concept, avoids the chaotic and endless renaming cascades required by the International Code of Nomenclature for algae, fungi, and plants. Because bluegrasses are often dominant species in their ecosystems, endless nomenclatural shifts create severe difficulties for geobotanists and ecologists.

Therefore, rather than proposing a formal new species concept in this manuscript, our current study deliberately steps away from strict alpha-taxonomy entirely. We focus instead on uncovering the broad genomic and demographic histories of these complex lineages.

- **Line 100: Apomixis is generally considered a breeding system (a shift from sexual to asexual reproduction). Consider rephrasing, including apomixis, together with decliny as another example of a shift in breeding system.**

  Reply: We have rephrased the sentence (**Lines 102–103**).

- **Line 147: The formulation about selecting the number of clusters is still not precise. The data can be (theoretically) divided into many clusters, which is why additional methods were developed to avoid subjectivity and help to determine the best number of clusters. Consider rewriting, e.g. "According to the clustering methods (UPGMA; unweighted pair … and STRUCTURE; Bayesian clustering method), the dataset can be optimally divided into two clusters (K=2, …). However, we also show results for /or we proceeded with K=3 (K=3, …), due to …. And the clusters are referred to as …".**

  Regarding the determination of the correct deltaK for the K=2, was a K=1 runs from STRUCTURE used for the Evanno method? While K=1 gives the same result, it is needed to correctly estimate the deltaK for K=2. Why were only K=1 to K=15 runs run? How did the clusters separate in higher Ks? Were the pure sub-clusters detected in higher Ks? Why not?

  Reply: We have rewritten this section using your suggested phrasing (**Lines 151–157**). Additionally, we confirm that $K = 1$ was indeed included in our STRUCTURE runs (Methods, **Line 793**).

  We did not investigate global runs at higher K values for two primary reasons:

  (1) Higher values were not supported by Evanno's delta K statistic (Rebuttal Fig. 1).
  (2) We followed the methodological recommendations of Evanno et al. (2005). The authors explicitly state that their method detects **the uppermost hierarchical level of population structure and they advise that subsequent analyses of subsets, defined by the best initial assignment of individuals to groups, are necessary to uncover hidden internal structure**. Following this recommendation, we performed a targeted investigation at a finer scale within each of the three main clusters separately, which identified 21 genetically cohesive subgroups (Supplementary Fig. 1). This hierarchical approach, first identifying primary lineages and then examining the internal structure within them, allowed us to capture meaningful genetic variation without forcing unstable higher K values across the entire dataset.

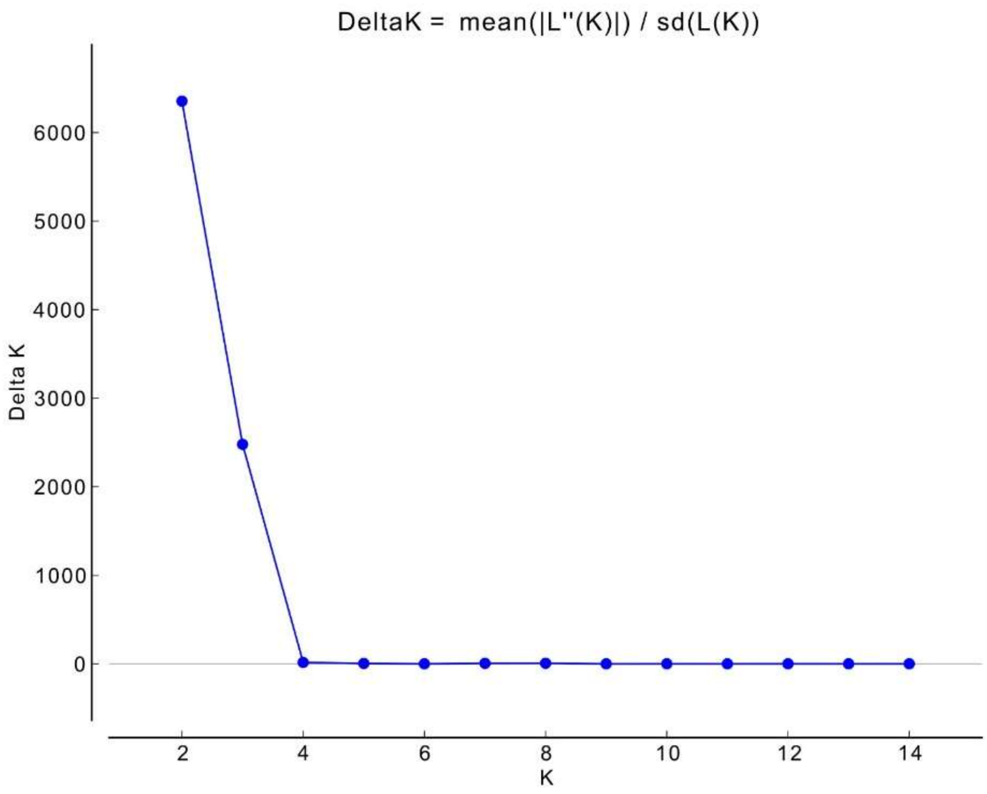

Figure 1. Evanno's delta *K* values for the STRUCTURE analysis presented in the main text. The plot shows two primary peaks at *K* = 2 (delta *K* > 6,000) and *K* = 3 (delta *K* ≈ 2,500).

- **Line 185: What is a morphospecies, and why is it used in this context? I am not sure how (and why) the authors got from morphospecies to divisions supported by a multispecies coalescent model.**

  Reply: By "morphospecies" we simply meant the 37 species that were initially identified by taxonomists based on morphological characters prior to any genetic analysis (i.e., traditional taxonomic determinations). To avoid confusion, we have replaced "morphospecies" with "morphologically defined species" in the revised text (**Line 189**). We then used hierarchical genetic clustering (STRUCTURE, PCA, Neighbour-net) to identify 21 genetically cohesive groups represented by 52 genetically pure individuals (membership coefficient Q ≥ 0.99). These groups were subsequently tested under the multispecies coalescent model (MSC) implemented in SNAPP. Thus, the MSC did not define the groups, it validated the genetic clusters identified through our hierarchical approach.

- **Line 196: I appreciate the authors' effort to rewrite these paragraphs following my previous comments. However, from the present text, I am not sure how to deal with two descriptions of the cluster geographic distribution - one general (line 155) and one derived from the sub-clusters. Please consider additional rewriting with highlighting the potential differences or similarities between the distributions.**

  Reply: Indeed, the connection was missing. We have revised this paragraph (**Lines 200–209**). We now explicitly state that the geographic distribution of the pure subgroups closely mirrors the broad regional patterns of their parent clusters while representing their core, non-hybridised habitats. This provides a clear link between the general cluster ranges and the specific origins of the evolutionary units.

- **Line 202: I am generally confused by this paragraph. What taxa does "these" refer to? What is a "stable genetic group"? Do the authors mean sites by populations in line 204 (population in this context implies mixed-species populations/sites, which remains unknown given the sampling)?**

  Reply: We have rewritten this paragraph (**Lines 211–212; 214–217**) to clarify these points. We now explicitly refer to "these 21 genetic groups" to link back to the previous paragraph, define them as "genetically cohesive groups identified in our phylogenetic analysis". Additionally, we have replaced the ambiguous term "populations" with "multiple geographic locations" to reflect our individual-based sampling. We have also added specific species names (*P. attenuata* and *P. botryoides*) to groups 18–21 for clarity.

- **Line 211: Please consider the wording here; STRUCTURE analysis does not identify the models or numbers of clusters; it shows the level of genetic admixture depending on the K number of clusters.**

  Reply: We have reworded it to "The STRUCTURE ancestry plot shows individual assignments at K = 2 and K = 3" (**Lines 222–225**).

- **Line 221: What is a pure genetic cluster? Do you mean an individual/individuals without admixture merged together? What about "between the genetically pure individuals from each cluster"?**

  Reply: By "pure genetic clusters" we refer to the clusters composed exclusively of individuals with membership coefficient $Q \geq 0.99$, as defined in the figure legend (**Line 226**). We have kept the terminology consistent throughout the legend.

- **Line 225: Please consider the same rewording here to avoid confusion from the overusage of the term cluster.**

  Reply: We have rephrased the title to "Geographic distribution and schematic division of pure genetic clusters into subgroups" to avoid overusing the term "cluster" (**Line 235**).

- **Figure 3: Why are only some species names shown on the map, and what were the selection criteria? The sub-clusters 6, 14 or 18 also have multiple accessions; all sub-clusters have 2 or more individuals, while there is only one dot shown on the map - is that due to the geographical proximity and potential graphical overlap?**

  Reply: You are correct, it was difficult to place all species names and individual IDs on the map without causing graphical overlap. We have updated Figure 3 in the main text and the high-resolution vector figure in Supplementary Data (via Figshare) to add species names for subclusters 5, 6, and 18, as their individuals originate from geographically separate locations. For subcluster 14 (*P. litvinoviana*), as with other subclusters shown as a single circle, the individuals were collected in close geographical proximity, so one label adequately represents them.

- **Line 230: Please elaborate on BBP.**

  Reply: BPP stands for Bayesian Posterior Probabilities, as now clarified in the text (**Line 215**) and figure legend (**Line 240**).

- **Line 235: Please clarify the sentence suggesting you used SNAPP to establish genetic groups.**

  Reply: We have rephrased the sentence (**Line 245**) to clarify that SNAPP was used to validate genetic groups identified by STRUCTURE, not to establish them jointly.

- **Line 248: Is phenotypic plasticity really a result of past gene flow? Phenotypic plasticity enables plants of the same genotype to quickly adapt to various or changing environments, without relying on the mutation/selection processes or a suitable inherited genetic combination. Consider, if you are not referring to morphological variation instead. Although these two are often difficult to tell apart without experimental cultivation, I think that the latter term would be more suited for a situation involving gene flow.**

  Reply: We agree that "morphological variation" will be more appropriate in this context, as our study does not experimentally test plasticity. We have revised the sentence accordingly (**Line 258**).

- **Line 264: I cannot open the interactive images here, although the links at the end of the manuscript worked. It does not mean anything other than my poor computer skills. I believe the link was tested by several of the authors. I am mainly pointing it our because in this case, I am not able to see why the reference to the original Figure 4 is missing and whether the figure is different from the same link in line 240 (the link there did not work for me either but as long as other people can open it, I appreciate the effort of the authors to make the Figures more easy to handle, at least in the digital version).**

  Reply: Well spotted! The Chart Studio platform, which previously allowed seamless deposition of objects produced in R, shut down its server at the end of 2025 (after the first draft of this manuscript was submitted). We have now deposited the interactive plot on GitHub and updated the links accordingly (**Lines 250 & 275;** niwdoog.github.io/Fig4a). While we respectfully disagree with the assessment of your computer skills, we appreciate the irony that helped us identify this issue and make these important corrections.

- **Line 272: Please clarify what the authors mean by pre-defined genetic clusters. While discriminant analyses work with pre-defined groups, are you still referring to the same 52 genetically pure individuals that were used for the PCA analysis? I am also not sure whether the wording is accurate in this paragraph. The authors are basically testing the ability of selected (pre-selected key) traits to distinguish the genetic clusters, not identifying the best characters by testing all available traits. I do not dispute the method, but I assume the authors are testing whether the selected traits are enough for clade differentiation. I think it should be clarified. Are the selected key traits common traits used in determination keys or taxonomic literature? Or are those traits the ones with the highest contribution to PCA axes?**

  Reply: The 21 traits used in the study are the most informative quantitative characters commonly used in taxonomic descriptions of *Poa* (we specify this in the Methods, **Line 829–830**). By "pre-defined genetic clusters" we refer to the same 52 genetically pure individuals from Clusters I–III used in the PCA. For LDA, we used the same traits as for PCA, except L2I (length of the second internode, from the top) and LLPB (length of the longest panicle branch), which were removed to reduce multicollinearity (Methods, **Lines 850–852**), as LDA is sensitive to collinearity.

  Thus, we use LDA to test whether a reduced set of morphological traits can accurately classify individuals into their pre-defined genetic Clusters. We use Recursive Feature Elimination (RFE)

to select seven key traits (Supplementary Tables 7, 8). In other words, this approach tests the predictive power of a parsimonious set of morphological characters to assign individuals to pre-defined groups.

We have revised the sentence to clarify these points (**Lines 282–284**).

- **Line 302: I am not sure phenotypic is the right word here, since the analyses employed are primarily intended to test morphological variation, not phenotypic. Even the selection of only genetically pure individuals does not mean all individuals from the same cluster are genetically uniform, and the tested phenotype would be stable/variable under different climatic conditions.**

  Reply: We agree that "morphological" is more accurate here, as our study does not experimentally test environmental influences on trait expression. We have revised the text accordingly (**Line 313**).

- **Line 303: I miss a context for the reassigned 80 individuals. Do the authors mean that integration of genetic data and identified/verified morphological traits enabled the further determination of an additional 80 individuals? Where were the 80 individuals reassigned? To the genetic clusters or to some species that were well differentiated within the clusters? Or did the authors try to determine the genetic cluster based on the morphological characters only and then double-check its genetic assignment?**

  Reply: By "reassigned" we mean that after comparing the initial morphology-based identifications (which were derived from historical herbarium labels) with our molecular data, we updated the species names of 80 individuals to reflect their genetic affinities.

  These reassignments allocated individuals to the most appropriate species name based on congruence between their genetic cluster membership, the morphological traits of core reference specimens within those clusters, and recent nomenclatural updates. For instance, as detailed further in our response to your next comment, specimens initially identified as the highly variable *P. albertii* that fell securely within genetic Cluster III were reassigned to *P. attenuata* following an expert taxonomic re-evaluation. Conversely, *P. albertii* specimens grouping within Cluster II retained their original classification (Supplementary Table 9).

  This represents a pragmatic solution. We acknowledge that morphological differentiation in this section remains highly cryptic and requires further formal scrutiny. However, as noted in the manuscript, we deliberately avoided a comprehensive taxonomic revision to maintain the focus of the study on broad evolutionary and demographic histories rather than detailed alpha-taxonomy. We have added a brief explanation of this process to the revised text (**Lines 313–319**).

- **Supplementary Table 9 shows no P. transbaicalica in the "Species name assigned by morphology" column. However, all samples assigned to P. stepposa were shown to belong to P. transbaicalica. How is that possible? How do the authors know after having genetic data that the previous assignment shall differ? What is the morphological difference between these two species, and why was it not spotted before obtaining the genetic data? Very similar is the situation for P. attenuata - how were the individuals with a known genetic background assigned to a species that was not determined in the "morphology column"? I understand if a single individual with a certain morphology falls into a uniform genetic group representing different species (also recognised in the morphology column. What I do not understand is how a group of individuals assigned by morphology to P. attenuata becomes P. transbaicalica in the genetic column without P. transbaicalica being recognised by morphology at all.**

  Reply: We sincerely appreciate the reviewer's meticulous examination of Supplementary Table 9. The reassignments you highlighted stem from a combination of recent nomenclatural updates and the high levels of morphological variation inherent to this group:

  - **Regarding *P. stepposa* and *P. transbaicalica*.** The names in the "initial morphology" column reflect the historical determinations written on the original herbarium labels. Historically, *P. stepposa* was widely used to identify these specimens. However, in the most recent and authoritative treatment of Russian grasses (Tzvelev and Probatova, 2019), *P. stepposa* was reduced to a synonym of *P. transbaicalica* based on the rules of nomenclatural priority. Both belong to the highly complex *P. versicolor* aggregate. Nonetheless, our study revealed two distinct genetic groups within Cluster III (group 11 with *P. transbaicalica* and group 13 with *P. stepposa*; Fig. 3 in the main text). Our genomic data strongly suggest that both names can represent true, distinct species; however, in-depth validation of their formal taxonomic boundaries is possible only with a larger sample size in future studies. We have therefore decided to keep Supplementary Table 9 unchanged to accurately reflect these distinct genetic lineages.
  - **Why the differences were not spotted earlier.** High morphological variation in *Stenopoa* completely obscures these boundaries. To illustrate this, Prof. Olonova previously conducted a field transplant observation where twenty dwarf plants were collected from a high alpine steppe and moved to a lowland forest zone. Within a single growing season, the dwarf plants grew into tall forms matching the descriptions of *P. stepposa* (Rebuttal Fig. 2; Olonova et al., 2019). Because such phenotypic variability erases subtle diagnostic traits in wild populations, reliably distinguishing these forms from dried herbarium material is nearly impossible without the context provided by genetic data.
  - **Regarding *P. attenuata* reassignments.** At the boundaries of their ecological ranges, highly variable species such as *P. botryoides* become morphologically indistinguishable from *P. attenuata*. Because hybridisation is also common, historical taxonomists often assigned these intermediate populations to presumed hybridogenic species like *P. albertii*. Our genetic data finally provided the objective framework needed to untangle these convergent physical forms, allowing us to reassign them to their true genetic lineages.

  To ensure this process is clear to the readers, we have updated the main text to explicitly state that our expert re-evaluation accounted for recent nomenclatural synonymy alongside the genetic data (**Lines 313–319**). Additionally, we have added Olonova et al. (2019) to the

Introduction (**Line 90**) to support the claim about phenotypic plasticity in *Stenopoa*, referencing the field transplant observation described here.

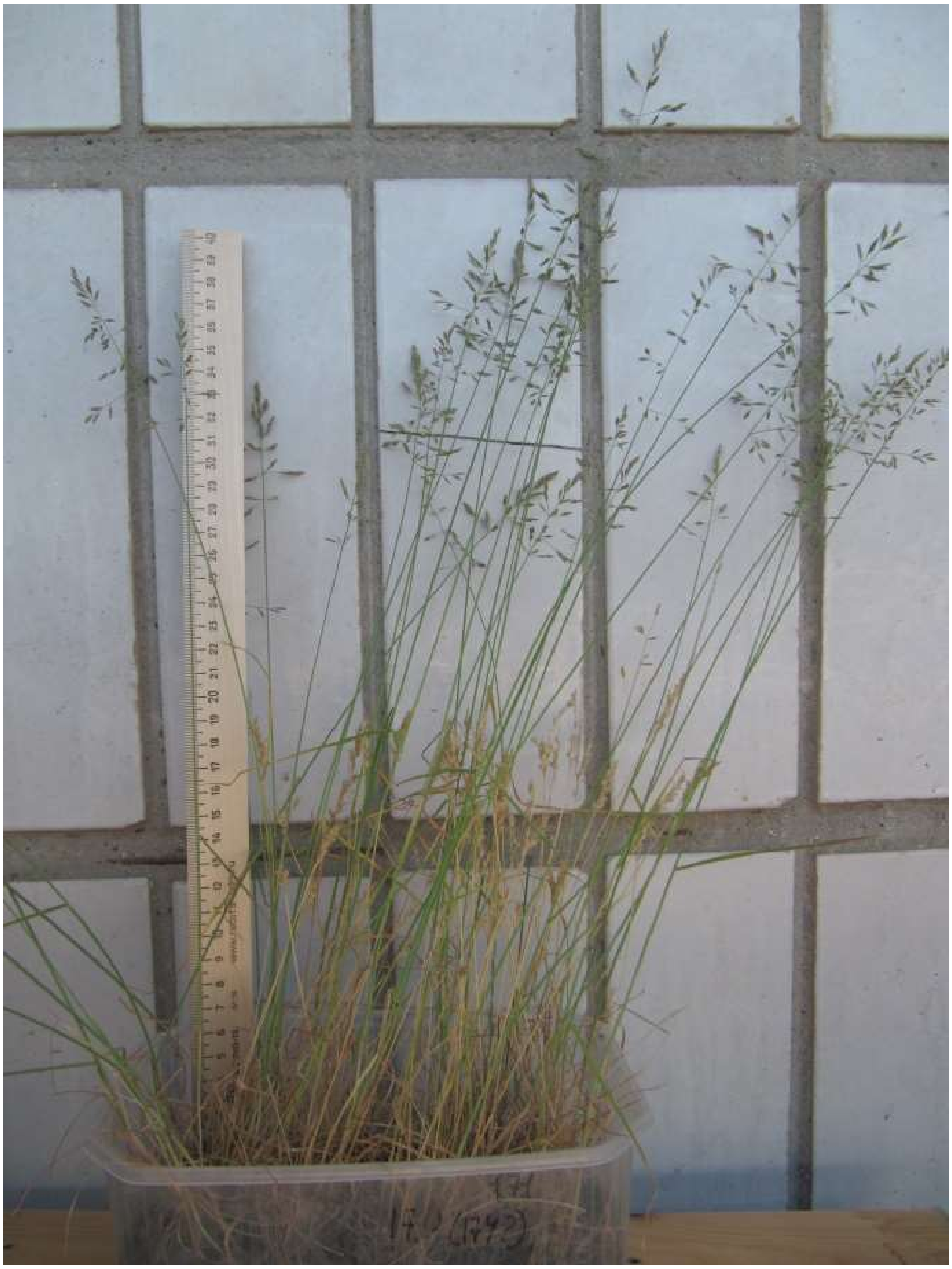

Figure 2. Common garden observation with *P. stepposa* (*P. transbaicalica*). Yellow shoots, which do not exceed 20 cm in length, represent growth from the previous year formed under arid conditions in south-eastern Altai. Green shoots developed from the same tussocks during a single summer season in Tomsk (Russia).

Reference

Olonova, M. V., Selezniova, A. E. & Romanets, R. S. Contribution to the study of dwarf xeromorphic species of *Poa* L. sect. *Stenopoa* Dumort. in the Republic of Altai. *Turczaninowia* **22**, 145–153 (2019).
https://doi.org/10.14258/turczaninowia.22.4.15

- **Line 324: Please provide references for GADMA2 and Starway Plot 2.**

  Reply: We have provided the references (**Line 340**).

- **Line 388: Please consider replacing kya with Kya to make it consistent with the remaining text. Same for the dating in the top pane of the Figure.**

  Reply: Following the reviewer's suggestion, we have standardised "kya" to "Kya" throughout the text, including in the Figure 5 caption (**Line 403**). The main figure and the high-resolution vector figure in Supplementary Data (via Figshare) have been updated accordingly.

- **Line 395: Please provide references for the MaxEnt modelling, AUC, and TSS.**

  Reply: We have added the requested references. For MaxEnt modelling, we now cite Phillips et al. (2020; **Lines 410**); for AUC/ROC, Hanley & McNeil (1982; **Lines 412** & **934**); for TSS, Allouche et al. (2006; **Line 413**).

- **Line 409: What are the unique adaptations of Cluster II to the monsoonal climate? If they are morphological, did the traits contribute to the clusters' differentiation in morphological analyses?**

  Reply: Our original use of the word "trait" was imprecise, as we were referring to the unique climatic parameter (low winter precipitation) of the monsoonal regime itself. We have corrected this in the text (**Lines 427–429**).

  Nonetheless, Cluster II does exhibit morphological traits likely adaptive to its monsoonal environment, and these traits contributed to its differentiation in our analyses. In the LDA (Fig. 4b; Supplementary Table 8), Cluster II is separated by flag leaf blade length (LFLB), panicle length (LP), distance to the uppermost node (DBUN), and plant height (HP). In the PCA and associated boxplots (Fig. 4a), Cluster II is characterised by significantly longer upper glumes (LUG) and flag leaf ligules (LFLL).

  While our original manuscript already highlighted how longer upper glumes may enhance wind dispersal during the short growing season, we have now expanded the Discussion to explicitly link the elongated ligule to these monsoonal conditions. Specifically, we note that the longer ligule likely acts as a physical barrier preventing water accumulation within the leaf sheath during heavy monsoonal rains (**Lines 548–549**).

- **Line 425: Please consider preplacing shrank by shranks to align the verb with the singular subject, Cluster II.**

  Reply: Because the sentence describes a historical process that began 11 Kya, the past tense form "shrank" is the appropriate and correct usage in this context.

- **Line 444: Should the word "Cluster" precede "the initial II-III ancestral line" in this sentence?**

  Reply: We agree that the original phrasing was slightly awkward. To improve clarity and avoid overusing the word "Cluster", we have rephrased the sentence to refer to "the ancestral lineage of Clusters II and III" and smoothed the second half of the sentence to avoid repetition (**Lines 463–464**).

- **Line 482: I suggest replacing the word lineage " with " clade/section, if applicable. Using lineage implies that the authors focused on a polyploid lineage within the Stenopoa section, which I believe is not the case.**

  Reply: To accurately reflect our focus on the entire *Stenopoa* group, we have replaced "lineage" with "clade". We appreciate this precise correction, as it significantly improves the clarity of our concluding thought (**Line 501**).

- **Line 487: I am wondering if mentioning the monsoonal preference/occurrence for Cluster II would also be of interest in this paragraph. I am also wondering if species is a proper word to use, given that several taxa were found polyphyletic, admixed, or there was a discrepancy between the initial and later assignment.**

  Reply: We have replaced the word "species" with "taxa" to better reflect these complex evolutionary units. Additionally, we have incorporated your suggestion to explicitly mention the monsoonal preference of Cluster II in this paragraph, which strengthens the link between our morphological and ecological findings (**Lines 506–507**).

- **Line 495: Please consider calling the data morphological instead of phenotypic, mainly due to the unknown character stability in different conditions.**

  Reply: Implemented (**Line 514**).

- **Line 496: Note that the selection of 3 clusters was a subjective decision of the authors, however well justified by geography. The Evanno test for the STRUCTURE analysis showed that K=2 is the best number of clusters. From this (objective/statistical) view, the genetic data confirmed the morphological ones.**

  Reply: This is a highly constructive note. We agree that from a strict statistical perspective, the primary genetic division supported by Evanno's delta K (K = 2) perfectly corroborates the two distinct morphotypes we identified. This strengthens the overall congruence of our dataset. We have revised this paragraph to explicitly highlight this objective concordance first, before discussing how the subsequent geographic and ecological subdivision (**Lines 515–519**).

- **Line 500: Please consider including morphological variation as well. It should not be omitted, especially if the phenotypic plasticity was not tested or documented in this group.**

  Reply: We have replaced "phenotypic plasticity" with "morphological variation" in the relevant sentence to accurately reflect the scope of our observations (**Line 522**).

- **Line 538: I appreciate the authors' efforts in changing the manuscript approach to polyploidy. I am wondering, why did the authors not consider autopolyploidy as another mechanism of polyploid formation? In a loose sense, autopolyploidy is not a WGD only within a population but also within a lineage/species. If there are more chromosome counts within the P. palustris lineage, the higher ones may as well arise from autopolyploidisation. Telling these two mechanisms (auto- vs. allo-polyploidy) apart is generally difficult and may be extremely hard in such a complex group, such as Stenopoa.**

  Reply: We completely agree that autopolyploidy is highly likely to be another major mechanism of polyploid formation in *Stenopoa*. To address this, we have explicitly incorporated autopolyploidisation into the text as an equally viable mechanism for these high even chromosome numbers, citing *Saccharum* (Garsmeur et al., 2011) as a comparative example.

We have also added a sentence acknowledging the inherent difficulty in distinguishing between allopolyploidy and autopolyploidy in such a complex group (**Lines 561–565**).

- **Line 566: Please consider rephrasing the exceptional ploidy levels by replacing them or adding the exceptional variation in chromosome numbers, which should better reflect the frequent aneuploidy and dysploidy.**

  Reply: We agree that "variation in chromosome numbers" is a much more accurate phrase, as it correctly encompasses the extensive aneuploidy and dysploidy observed in this section alongside WGDs. We have replaced the phrase accordingly (**Line 591**).

- **Line 593: Please consider replacing "identifies" with "identified" - most of the text is in the past tense, and I do not see a reason why it should be different here.**

  Reply: Corrected (**Line 618**).

- **Line 597: Since the paragraph in the Introduction nicely showed that the number of species largely depends on the taxonomic approach, can you somehow estimate the proportion these two species represent in the Stenopoa section or in your dataset? The literature listing the chromosome counts should mention the source populations used for the chromosome counting - is it possible to trace the species or the diploid populations, and if so, are the populations (or at least the species) still persisting in the regions the authors identified as the possible regions of Stenopoa origin? If so, it would be a nice confirmation of the presented hypothesis.**

  Reply: The source populations for the published diploid counts are Gaydary Village (Kharkiv Oblast, Ukraine; elevation approximately 180 m) for *P. palustris* (Cluster I) and Baihua Mountain (Beijing, China; elevation approximately 1,800 m) for *P. orinosa* (Cluster II). Both localities align with suitable habitats projected by our ecological niche models. However, we opted not to include the percentage of these species (2 of 37, ~5%) in the main text because this figure is of limited informational value given the profound taxonomic ambiguity within *Stenopoa*, and it does not substantially contribute to the central narrative of the study.

  More importantly, we wish to emphasise that these published diploid counts originate from regions outside the specific ancestral divergence zones and refugia areas we identified (the Russian Far East and the Tibetan Plateau). As we note in the revised text, ploidy data from these critical refugial areas are currently lacking. We therefore highlight that future work should target these specific regions to search for diploid populations, as locating them there would provide the strongest independent test of our refugial hypothesis (**Lines 619–626**).

- **Line 624: Is there any study on Poa or Stenopoa actually testing for phenotypic plasticity? It was not acknowledged in the text, and as I mentioned in my previous comments, morphological variation and phenotypic plasticity are often difficult to tell apart without experimental work.**

  Reply: To our knowledge, only one preliminary transplant study has been conducted within this section (Olonova et al., 2019, mentioned above). Nonetheless, to maintain a strictly descriptive tone regarding our current non-experimental data, we have followed your suggestion and replaced "phenotypic plasticity" with "morphological variation" where appropriate throughout the manuscript (**Lines 132** & **189**; **653–654**). We have also updated the future research section to explicitly acknowledge this lack of experimental data and to suggest common garden cultivation as a necessary next step (**Lines 665–666**).

- **Line 640: Where in the phylogeny is the closest reliable fossil? What closest group has a time-calibrated phylogeny, and could those species be included in future projects as outgroups to obtain better calibration, e.g. with molecular clock/ mutation rate methods that do not rely on primary calibration points, i.e. fossils?**

  Reply: Unfortunately, the earliest reliable macrofossils specifically assigned to the genus *Poa* is known from western North America and dates back only 20,490 years before present (Soreng & Van Devender, 1989). There are no fossil records for the section *Stenopoa.* As stated by Hoffmann et al. (2013), which we used as a primary reference for our study: "Unambiguous fossils of the *Poa* alliance are unfortunately missing, thus we had to apply a secondary calibration of our data. Absolute ages of the clades were calculated by fixing the split between the outgroup taxa *Phalaris* and *Avena* and the ingroup of 20 and 25 Myr, respectively". Furthermore, a recent comprehensive study by Soreng et al. (2022) summarised several other attempts to estimate divergence dates for *Poa* and performed analyses in a broader context (the PPAM clade). However, that study focused entirely on plastid data, and it is well established that nuclear substitution rates often vary significantly from plastid rates.

  Nonetheless, we agree that incorporating fossil-calibrated phylogenies of close relatives (e.g., within Pooideae) would be a valuable future direction, and we have added a sentence to the limitations section acknowledging this (**Lines 670–675**).

  Reference
  Soreng, R. J., & Van Devender, T. R. Late Quaternary Fossils of *Poa fendleriana* (Muttongrass): Holocene Expansions of Apomicts. *The Southwestern Naturalist* **34**, 35–45 (1989).
  https://doi.org/10.2307/3671807

- **Lines 688 - 692 nicely illustrate why I asked for more details about the protocol and called the Kilian et al (2012) an outdated reference - while it may describe the principle of the method (and is used in this context in the references kindly provided by the authors in their Rebuttal letter), the protocol the company uses is very much different from the one described in the paper and these modifications should have been acknowledged. Given the stark difference in details given for the "simple step", such as DNA extraction and the "complex step", such as the library preparation, I suggest adding (line 689) the library preparation "done by Diversity Arrays … following their internal protocols" included …**

  Reply: As suggested, we have updated the text (**Lines 744–745**) to explicitly state that library preparation was conducted by Diversity Arrays Technology following their internal protocols.

- **Line 720: The SNP abbreviation should be elaborated when first mentioned in the text, not here.**

  Reply: We have now defined the SNP abbreviation at its first occurrence in the manuscript (Results, **Line 149**) and removed the elaboration from the Methods section.

- **Line 737: I am not sure why this is a new paragraph - method details for UPGMA are given in the previous one, while PCA and NeighbourNet, together with STRUCTURE, are given in this one.**

  Reply: We have merged the first two paragraphs so that all primary clustering and network methods (UPGMA, STRUCTURE, Neighbor-Net, and PCA) are described together in one cohesive block (**Line 790**).

- **Line 746: Please consider a new paragraph for the new methods/analyses.**

  Reply: Implemented (**Line 802**).

- **Line 755: Please consider clarification of what the pure clusters mean, e.g., "pure individuals within each cluster", and replacing "individually" with "separately".**

  Reply: Implemented (**Line 810**).

- **Line 808: Please check for possibly missing word(s) after coalescent.**

  Reply: To avoid any ambiguity, we have revised the sentence to explicitly refer to the coalescent model (**Line 862**).

- **Lines 819-825: Unlike other paragraphs describing the parameters used for a particular method, this one describes the method itself. Please consider unifying this paragraph with the others.**

  Reply: We have removed the purely descriptive text and merged the core structural settings (the use of epochs, growth types, and automatic selection parameters) directly into the subsequent paragraph. This unifies the text to focus strictly on the parameters and strategy applied in our GADMA analyses (**Lines 873–875**).

- **Line 834: Please replace these populations with the genetic clusters here and throughout the paragraph where appropriate (everywhere except population size dynamics in line 842).**

  Reply: We agree that referring to "genetic clusters" is more consistent with the terminology used throughout the manuscript. We have updated the text throughout this paragraph (**Lines 884**, **890–892**).

- **Line 834: Does the trajectory here refer to the dynamics in the previous sentence? If so, please consider using the same term for better clarity. If not, please elaborate on trajectories in the first model.**

  Reply: In this context, "trajectories" indeed referred to the population size dynamics inferred in the first model. We have updated the wording to ensure consistency and clarity throughout the paragraph (**Line 887**).

- **Line 849: It seems like the authors (Grower et al) refer to it as Demes. Please do the same.**

  Reply: Implemented. We have updated the terminology to refer to Demes and corrected the misspelling of the author's name from Grower to Gower (**Line 899**).

- **Line 856: Were the spatial duplicates from the same genetic cluster or from different ones? Similarly, were the records within the 1km2 grid cell from the same cluster? If not, I wonder if keeping both records would not yield a more accurate paleodistribution reconstruction/prediction.**

  Reply: We performed the removal of spatial duplicates and the subsequent spatial thinning within each genetic cluster separately, rather than across the pooled dataset. We have updated the Methods section to make this distinction explicit (**Line 906**).

- **Line 870: Additionally to what?**

  Reply: We have rephrased the sentence for the better clarity (**Lines 920**).

- **Line 875: The list of climatic variables would better fit into the result section of the manuscript.**

  Reply: We agree that the explicit list of the six key climatic variables (after VIF and correlation filtering) is better presented in the Results section, where model performance and variable contributions are discussed. We have therefore removed the list from the Methods and inserted it in the Palaeodistribution prediction subsection of the Results (**Lines 416–420**; **925–927**).

- **Line 887: From the context, I assume the terms taxa and species used here actually refer to the three genetic clusters. Please clarify. If it refers to the species, are those taxa or species the species assigned to the pure sub-clusters identified in Supplementary material 1? Similarly, do the species in line 888 refer to certain Stenopoa species or to the three clusters mentioned at the beginning of this methods section? How were the species' known ecological preferences and environmental conditions determined? From floristic literature? Can the authors provide a list of references somewhere? Do they correspond to the reconstructed ecological preferences and environmental conditions of the clusters?**

  Reply: We have clarified the terminology in this section by replacing "taxa" with "genetic clusters" to ensure consistency with our methodology (**Lines 936–937**). The established ecological preferences were determined from major regional floristic works, specifically the Flora of the USSR (Tzvelev, 1976), the Flora of China (Liu, 2003), and previous taxonomic revisions of the section (Olonova, 2016). We can confirm that the environmental requirements and habitats reconstructed by our niche models aligned closely with the independent botanical descriptions provided in these floras (**Lines 938–940**).

- **Line 896: Please clarify what the Statistical significance of the cluster term means. Should it be nugget instead?**

  Reply: The "nugget term" refers to the spatial covariance structure parameter, which is used to correct for spatial autocorrelation and is selected via AIC model comparison. Conversely, the "cluster term" is the fixed effect of genetic cluster itself, whose statistical significance is tested separately with ANOVA on the final model. We have clarified both terms and their distinct roles in the revised text to prevent any future confusion (**Lines 947–951**).

- **Line 908: I appreciate the work the authors did on this Table.**

  Reply: We thank the reviewer for this positive comment. This suggestion in the previous round helped us scrutinise the data more thoroughly and improve the presentation of this table.

##

Referee #2: Evolution and breeding system variation in genus Poa

Reviewer #2 (Remarks to the Author):

##

- **The ms is siginifcantly improved**
  I wonder why this Tzvelev article is not commented on?
  Tzvelev, N. N. 2009. On species of the genus Poa L. section Stenopoa Dumort. (Poaceae) in the eastern Europe. Novosti Sist. Vyssh. Rast. 41: 18–52.
  It would seem to deserve a comment:

  Tzvelev recognize many Stenopoa species in Eastern Europe former USSR region.
  Poaceae Poa hypanica Prokudin Accepted By
  Poaceae ! Poa nemoralis L. Accepted By
  Poaceae Poa lapponica Prokudin Accepted By
  Poaceae Poa palustris L. Accepted By
  Poaceae Poa volhynensis Klokov Accepted By
  Poaceae Poa urssulensis Trin. Accepted By
  Poaceae ! Poa tanfiljewii Roshev. Accepted By
  Poaceae Poa podolica (Błocki ex Asch. & Graebn.) Błocki ex Zapał. Accepted By
  Poaceae Poa versicolor Besser Accepted By
  Poaceae ! Poa polonica Błocki Accepted By
  Poaceae Poa sterilis M. Bieb. Accepted By
  Poaceae ! Poa biebersteinii H.N. Pojark. Accepted By
  Poaceae Poa erythropoda Klokov Accepted By
  Poaceae Poa transbaicalica Roshev. Accepted By
  Poaceae ! Poa glauca Vahl Accepted By
  Poaceae Poa bryophila Trin. Accepted By
  Poaceae Poa balfourii Parn. Accepted By
  Poaceae Poa janczewskii Zapał. Accepted By
  Poaceae Poa compressa L. Accepted By
  Poaceae ** Poa taurica H.N. Pojark. Accepted By
  Poaceae Poa rehmannii (Asch. & Graebn.) Woł. Accepted By
  Poaceae Poa austrouralensis Tzvelev Published In
  Poaceae Poa nemoralis var. popovii Tzvelev Published In
  Poaceae Poa nemoralis var. zhitomirica Tzvelev Published In
  Poaceae * Poa orientalis Tzvelev Published In
  Poaceae Poa kulikovii Tzvelev Published In
  Poaceae Poa lapponica var. turiensis Tzvelev Published In
  Poaceae Poa egorovae Tzvelev Published In
  Poaceae Poa klokovii Tzvelev Published In
  Poaceae Poa palustris var. halophila Tzvelev Published In
  Poaceae Poa sterilis var. bodrakense Tzvelev Published In
  Poaceae Poa sterilis var. juzepczukii Tzvelev Published In
  Poaceae Poa paczoskii Tzvelev Published In
  Poaceae Poa saksonovii Tzvelev Published In
  Poaceae Poa korshinskii Tzvelev Published In

Poaceae Poa korshinskii var. birskensis Tzvelev Published In
Poaceae Poa korshinskii var. capitolinae Tzvelev Published In
Poaceae Poa igoshinae Tzvelev Published In

Reply: We sincerely thank the reviewer for pointing out this highly relevant publication.

We agree that this work provides critical context for the taxonomic complexity of *Stenopoa* in Eastern Europe. We have now incorporated this reference into the Introduction (**Lines 96–97**) and have added it to our reference list. Although the article is occasionally cited as 2009 in some works, the journal official record confirms a publication date of 2010 (https://www.binran.ru/en/publications/novosti-sistematiki-vysshyh-rastenij/2050/15064). Moreover, we have replaced Olonova (2016) with Tzvelev (1976) to cite the original source for the broad morphological species concept in this region (**Line 100**).

Additionally, while we agree that a deeper analysis of this work would provide additional taxonomic context, we have limited our discussion to its role in illustrating the broad range of regional diversity estimates. Delving into the specific nomenclature of the Eastern European taxa would necessitate an equally detailed analysis of other regional treatments, such as those for the Russian Far East (e.g., Probatova, 2015), which would significantly increase the length of the manuscript and distract from the primary focus on evolutionary and genomic dynamics. As the objective of this study was not to provide a critical taxonomic revision, we have reserved that detailed analysis for a separate forthcoming publication that will integrate these genomic results with traditional regional nomenclature.

Reference

Probatova, N. S. The genus *Poa* L. (Poaceae) in the Russian Far East (as part of the "Poaceae of Russia" project). *Komarov Memorial Lectures* **63**, 8–68 (2015).

##

Referee #3: Plant evolution and population genomics

Reviewer #3 (Remarks to the Author):

##

I appreciate the efforts made by the authors to thoroughly address the issues raised, including both my comments and those of the other reviewers. I find the response satisfactory and have no further concerns.

> **Reply:** We are very grateful to the reviewer for their constructive feedback during the previous round and for the positive evaluation of our revised manuscript.

##

We would like to thank all the reviewers for their time and for their feedback on the previous versions of the manuscript. We also thank Reviewer #1 for providing a few new helpful comments, which have polished our final text. For your convenience, the original comments are presented in bold type, and each of our responses is indented below the corresponding comment.

##

Reviewers' comments:

##

Referee #1: Plant systematics and ecology

Reviewer #1 (Remarks to the Author):

##

This is my third review of the manuscript "Pleistocene adaptation and diversification in cold, arid environments: genomic and paleoclimate insights from non-model bluegrasses" by Dr. Baiakhmetov and colleagues (Reviewer #1 in the previous revisions).

I greatly enjoyed reading the presented revised manuscript. The great amount of work the authors did in the first version of the manuscript is now presented in a more complex synthesis, focusing on showing a clear story about how the interplay of climatic changes, morphological variation and possible phenotypic plasticity together with polyploidy and hybridization shaped demographic history and diversification of a taxonomically complicated group. The expanded discussion section about possible evolution and distribution under future changing climate adds to the manuscript significance and further elevates the already interesting results presented in the previous versions.

The authors found a good balance between providing enough details about the complex group and the difficulties it poses without overloading the text with too many taxonomical details. Thus, while the text clearly admits the limitations and sets directions for future studies, it it a very good standalone work of great interest for anyone interested in ecological and morphological adaptation, Pleistocene flora evolution, hybridization and also their joint effect on diversification and evolution.

> **Reply:** We appreciate your feedback and are glad to hear that you found the revised manuscript enjoyable and improved.

I appreciate the efforts the authors made to thoroughly address my comments. I still have a few suggestions the authors may consider to review, most of them being more about editing the text.

- **Most part of the first paragraph is polyploidy-oriented, which I believe is a residue from the first version of the manuscript which stressed polyploidy. In this version, I feel like it draws more focus on polyploidy than is actually needed and I was wondering if a paragraph focusing more on the interplay between e.g., morphology and environment or climatic changes driving the flora evolution or migrations in the study area would be more appropriate. On the other hand, the paragraph is nice and clear and except for possibly steering the expectations of the readers from the actual aims toward polyploidy, there is nothing "wrong" with the paragraph if the authors decide to keep it.**

> **Reply:** While we agree that it places a strong emphasis on polyploidy, we believe this focus remains crucial for setting the foundation of our study. Polyploidy is a fundamental evolutionary mechanism in the genus and directly facilitates the morphological variation and plasticity (a subject for further work), niche expansion, and climatic adaptation that we explore throughout

the manuscript. We feel this narrative provides necessary context for the complexity of *Poa* and section *Stenopoa* in particular. Thus, we have opted to keep the paragraph in its current form, which you kindly noted is acceptable.

- **I was wondering if the result section about Genetic structure and molecular dating could after the first sentence (lines 149-150) continue with lines 189-191 or 192, that briefly present the methodological framework before jumping directly into the STRUCTURE results?**

  Reply: To address this while maintaining the logical flow of the results, we have made the following revisions. First, we now explicitly introduce the 37 morphologically defined species in the very first sentence to provide immediate context (**Line 134**). Second, we have restructured the transition to the hierarchical analysis at the end of the section to clearly explain why the subsequent fine-scale approach was necessary (**Lines 176–182**).

- **Lines 159-167 describe the geographic distribution of Clusters I and III, however, it is not immediately clear that Cluster II distribution is actually already provided in line 155. Please consider moving the details about Cluster II distribution into the following paragraph.**

  Reply: Corrected. We have moved the details regarding the geographic distribution of Cluster II into the subsequent paragraph alongside the descriptions for Clusters I and III to improve clarity (**Line 140**, **Lines 146–148**).

- **Line 215: Why the two species mentioned here do not have the author 's name while the two other species mentioned in this paragraph do?**

  Reply: Corrected. We have added the respective taxonomic authorities to these two species (**Line 204**).

- **Line 717: Please consider deleting "years" after 2022.**

  Reply: Done (**Line 643**).
